# Role of diet in stroke incidence: an umbrella review of meta-analyses of prospective observational studies

**DOI:** 10.1186/s12916-022-02381-6

**Published:** 2022-05-24

**Authors:** Na Guo, Ying Zhu, Dandan Tian, Yating Zhao, Chenguang Zhang, Changqing Mu, Chen Han, Ruixia Zhu, Xu Liu

**Affiliations:** Department of Neurology, First Affiliated Hospital of China Medical University, No. 155 North Nanjing Street, Shenyang, 110001 Liaoning China

**Keywords:** Dietary factor, Stroke, Prospective observational study, Meta-analysis, Umbrella review

## Abstract

**Background:**

Stroke is one of the major challenges for the global healthcare system, which makes it necessary to explore the relationship between various modifiable factors and stroke risk. Recently, numerous meta-analyses of prospective observational studies have reported that dietary factors played a key role in the occurrence of stroke. However, the conclusions of previous studies have remained controversial and unclear. Accordingly, we conducted an umbrella review synthesizing and recalculating available evidence to assess the certainty of the associations between dietary factors and stroke.

**Methods:**

Relevant meta-analyses examining the associations between dietary factors and stroke were searched in PubMed and Embase databases up to September 1, 2021. For each eligible meta-analysis, two independent reviewers appraised the methodologic quality using the AMSTAR 2 criteria and estimated the summary effect size, 95% confidence intervals, 95% prediction intervals, heterogeneity between studies, and small-study effects. Moreover, we further assessed the associations between dietary factors and ischemic stroke as well as hemorrhagic stroke. Lastly, a set of pre-specified criteria was applied to qualitatively evaluate the epidemiological credibility of each dietary factor.

**Results:**

Overall, our umbrella review included 122 qualified meta-analyses for qualitative synthesis, involving 71 dietary factors related to food groups, foods, macronutrients, and micronutrients. Using the AMSTAR 2 criteria, 5 studies were assessed as high quality, 4 studies as moderate quality, and 113 studies as low or critically low quality. We identified 34 dietary factors associated with stroke occurrence, 25 dietary factors related to ischemic stroke, and 11 factors related to hemorrhagic stroke. Among them, high/moderate certainty epidemiological evidence demonstrated an inverse association between intake of fruits (*RR*: 0.90) and vegetables (*RR*: 0.92) and stroke incidence, but a detrimental association between red meat (*RR*: 1.12), especially processed red meat consumption (*RR*:1.17), and stroke incidence. Besides, the evidence of high/moderate certainty suggested that the intake of processed meat, fruits, coffee, tea, magnesium, and dietary fiber was associated with ischemic stroke risk, while consumption of tea, fruits, and vegetables was relevant to hemorrhagic stroke susceptibility.

**Conclusions:**

Our study has reported that several dietary factors have a significant impact on stroke risk and offered a new insight into the relationship between dietary modification and stroke occurrence. Our results may provide an effective strategy for stroke prevention.

**Supplementary Information:**

The online version contains supplementary material available at 10.1186/s12916-022-02381-6.

## Background

Stroke, a global health issue, is the major cause of permanent disability and death worldwide, resulting in a substantial economic burden on individuals, families, and society [1, 2]. With the aging of the global population, the American Heart Association estimates that the incidence of stroke in American adults may reach 4% by 2030, causing stroke-related medical expenses rising to $183 billion [3]. Thus, effective prevention and management strategies of stroke are urgently needed in order to limit the prevalence and cost of stroke. To our knowledge, recent studies have found that many unmodifiable factors were associated with stroke risk, including age, gender, family history, and so on [4]. Moreover, modifiable factors also play a vital role in stroke susceptibility.

Dietary factors, an important part of modifiable factors for stroke occurrence, have attracted intense interest of researchers and clinicians. An increasing number of meta-analyses from prospective observational studies were conducted to examine the effects of dietary factors on the risk of stroke. Nevertheless, findings of previous meta-analyses (including the conclusions, strength of evidence and potential bias, etc.) investigating the association between dietary factors and stroke susceptibility were sometimes discordant and inconclusive. Therefore, it was necessary to conduct a comprehensive evaluation of all published meta-analyses to summarize and clarify the relationship between dietary factors and stroke risk.

An umbrella review, providing a systematic calculation and appraisal of meta-analyses, has been widely applied to evaluate the association between modifiable factors and disease susceptibility including dementia [5], multiple sclerosis [6], and various cancers [7, 8], thereby improving the accuracy and strength of results and revealing the breadth and robustness of associations [9]. Till now, an umbrella review investigating the association between dietary factors and stroke risk has not been conducted; hence, in order to further understand and reassess the association, we carried out a comprehensive umbrella review through collecting all available meta-analyses of prospective observational studies to explore potential strategies for stroke prevention.

## Methods

### Literature retrieval strategy

The search strategy of our umbrella review adhered to the Preferred Reporting Items for Systematic Reviews and Meta-Analyses (PRISMA) guidelines [10]. The following search terms were applied by searching for titles and abstracts in PubMed and Embase databases up to September 1, 2021: (“diet” OR “dietary” OR “intake” OR “consumption”) AND (“stroke” OR “cerebrovascular disease” OR “ischemic stroke” OR “hemorrhagic stroke” OR “cerebral infarction” OR “brain infarction” OR “cerebral hemorrhage”) AND (“meta-analysis” OR “systematic review”). Besides, the reference lists of retrieved articles were carefully screened to search for potentially eligible articles.

### Inclusion and exclusion criteria

Studies that met the following criteria were included in our umbrella review: (1) meta-analyses of prospective observational studies (cohort studies or nested case-control studies) investigating the relationship between dietary factors and stroke risk; (2) eligible dietary factors consisting of two types: one is food groups, foods, and beverages (grains, vegetables, fruits, fish, meat, eggs, legumes, nut, dairy products, chocolate, coffee, tea, and sugar-sweetened beverages), and the other is macronutrients (protein, fat, carbohydrates, and fiber), micronutrients (vitamin, mineral), and flavonoid; (3) considering the incidence of stroke, ischemic stroke, or hemorrhagic stroke as the outcome; (4) providing the specific data to calculate the summary effect size, 95% confidence intervals (CIs), 95% prediction intervals (PIs), heterogeneity between studies, and small-study effects for further analysis; and (5) published in English. In contrast, articles were excluded based on the following criteria: (1) meta-analyses of non-prospective observational studies, including randomized controlled trials, cross-sectional, or non-nested case-control studies; (2) without original data to analyze the summary risk estimate, 95% CIs, 95% PIs, etc. (e.g., systematic reviews without meta-analysis); (3) reviews, letters, editorials, and conference abstracts; and (4) duplicated publications.

### Data extraction and methodological quality assessment

Two authors respectively collected the following data for each eligible article using a standard extraction form, including the first author’s name, publication year, study design, original article retrieval time, dietary factor, outcome of interest (stroke, ischemic stroke, or hemorrhagic stroke), number of included studies, number of participants and cases, comparison of types (high versus low meta-analysis or dose-response meta-analysis), duration of follow-up, assessment tool of the original study, information of funding, and conflict of interest. Moreover, we extracted the most fully adjusted effect estimates and corresponding 95% CIs from original studies. According to the literature, the most important adjustment factors in the investigation between dietary factors and stroke susceptibility included age, gender, body mass index, physical activity, total energy intake, smoking, hypertension, and diabetes mellitus.

The AMSTAR (A MeaSurement Tool to Assess systematic Reviews) 2, a reliable methodological quality assessment tool, was applied to evaluate the quality of each eligible meta-analyses [11]. It was composed of 16 items, which were divided into 7 critical and 9 non-critical domains. According to the quality of each item, including search strategy, study selection, data extraction, study limitations, risk of bias assessment, etc., we further categorized each systematic review into high, moderate, low, or critical low quality.

### Statistical analysis

In this umbrella review, the random and fixed effect models were applied to calculate the summary effect size and 95% CIs to assess the association between dietary factors and stroke risk. We further computed the 95% PIs, which represented the probability range in which the effect estimates from future studies investigating the same association would lie with 95% certainty [12]. Then, the Cochran *Q* test and *I*^*2*^ statistic were also performed to analyze the statistical heterogeneity between original studies, and *P* < 0.10 and *I*^*2*^ > 50% were deemed to be high heterogeneity. Moreover, Egger’s test and funnel plot were applied to evaluate the small-study effect and publication bias for each eligible meta-analysis by using statistical and graphical tests. The results of *P* value < 0.10 were considered to be significant evidence of small-study effects. Lastly, we carried out subgroup evaluation according to stroke subtypes, namely ischemic stroke and hemorrhage stroke. All statistical analyses were conducted using STATA software 12.0. Apart from heterogeneity and small-study effects, all tests were considered to be significant at the level of *P* value < 0.05.

### Credibility of epidemiologic evidence

In accordance with established tools applied in previous umbrella reviews, we appraised the strength of epidemiologic evidence for the relationship between each dietary factor and stroke risk by using the following criteria: (1) precision of the estimate (*P* value < 0.001, a threshold with less false-positive possibility); (2) number of cases > 1000; (3) no significance heterogeneity (*P*_heterogeneity_ > 0.10 and *I*^*2*^ < 50%); and (4) no evidence of small-study effect (*P*_*E*gger_ > 0.10). We quantified the epidemiologic evidence as high credibility (if all the above criteria were met), moderate credibility (if *P* value < 0.001 was found and two of the remaining three criteria were satisfied), weak credibility (all other cases with *P* value < 0.05), and nonsignificant association (*P* value > 0.05) [13].

## Results

### Study identification

**Fig. 1:**
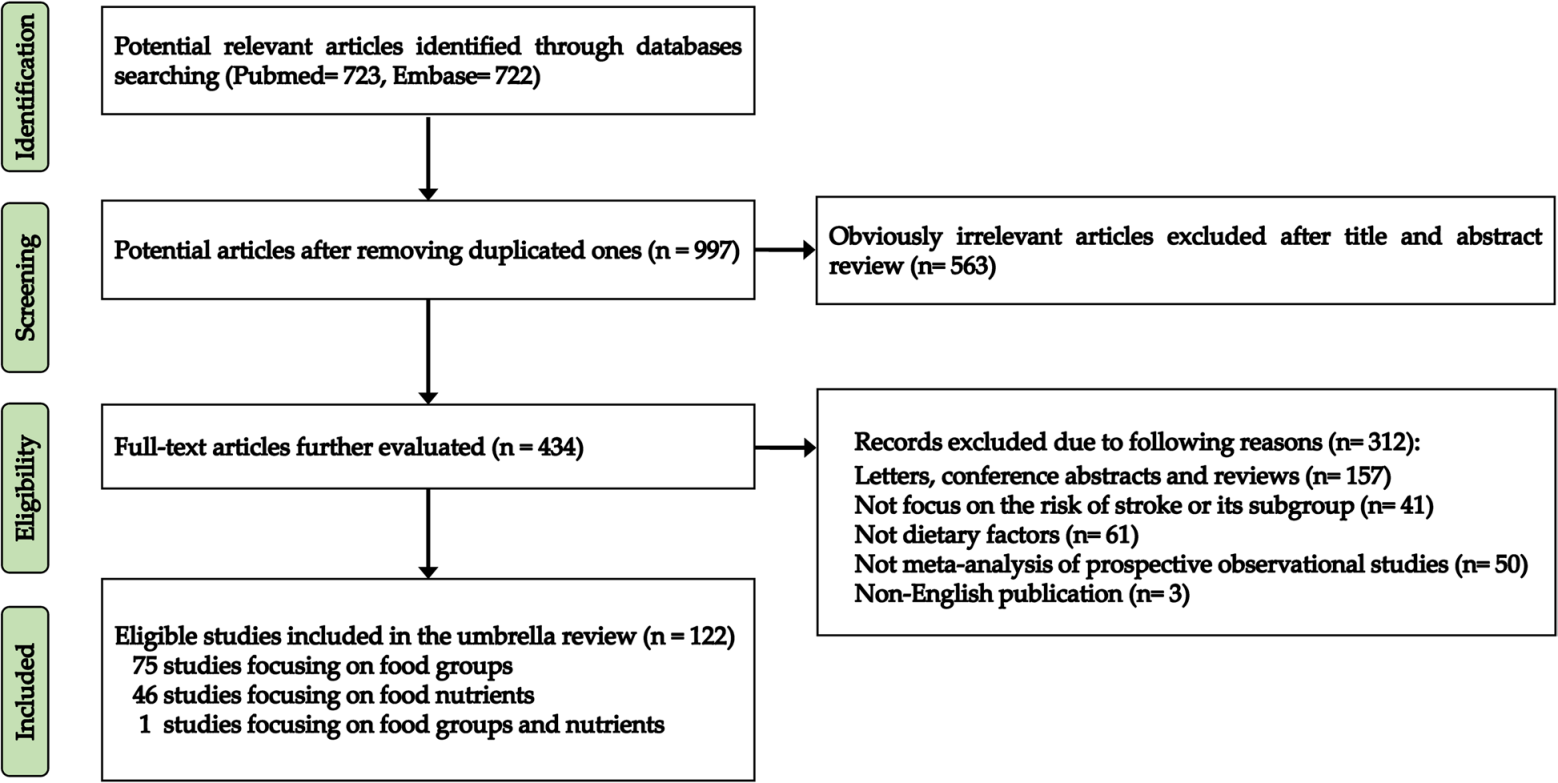
Flow diagram of the literature selection process

Overall, 1445 related articles were initially retrieved from PubMed and Embase databases after the systematic search. First, 448 duplicated publications and 563 irrelevant publications were removed through browsing the title and abstract. Then, after a full-text review, we excluded a total of 312 articles, including 157 conference abstracts, letters, and reviews; 61 not relevant to dietary factors; 41 not focused on stroke risk; 3 not written in English; and 50 meta-analyses involving non-prospective studies. Moreover, all excluded full-text articles are detailed in Additional file 1: Table S1. Finally, 122 qualified meta-analyses were enrolled in our umbrella review, and the associations between foods, food groups, and food nutrients and stroke susceptibility were extracted and listed in Additional file 2: Table S2 and Additional file 3: Table S3 [14–135], respectively. The flow chart of the selection process for eligible meta-analyses is presented in Fig. 1.

### Characteristics of included studies

A total of 228 effect estimates were reported in all eligible meta-analyses examining the relationship between dietary consumption and stroke risk. All eligible articles were published between 2004 and 2021. The median number of included meta-analyses per dietary factor was 3 (range 1–12). Besides, the evidence of each meta-analysis was based on median 7 original studies (interquartile range 4–10, range 2–40), median 253,511 participants (interquartile range 173,274–354,718, range 20,089–4,381,604), and median 6978 stroke cases (interquartile range 4260–10,192, range 299–46,951).

If more than one meta-analysis were available to assess the same dietary factor, the one with dose-response analysis was selected in the main analysis. Then, when more than one published dose-response meta-analysis for the same association, the one with the largest number of participants was preferred. Thus, the main analysis for dietary factors retained 71 risk estimates, including 40 food groups, foods, and beverages and 31 macronutrients and micronutrients. Moreover, of the 71 dietary factors, 41 dose-response relationships were available, among which 31 provided the information of the linearity of the dose-response relationships (e.g., *P* for non-linearity). Five of these 31 dose-response relationships indicated non-linearity, including vegetables, red meat, nut, vitamin E, and magnesium. Additionally, in the main analysis based on stroke subtypes, 44 risk estimates were retained to analyze the association between dietary factors and ischemic stroke, and 30 risk estimates focused on the influence of dietary factors on hemorrhagic stroke.

### Methodological quality assessment of meta-analyses

The meta-analyses included in our umbrella review were assessed for methodological quality, with 5 studies being considered as high (4.10%), 4 studies as moderate (3.28%), and 113 studies as low (43 studies, 35.25%) or critically low (70 studies, 57.38%) (see Additional file 4: Table S4). The common critical flaws in most meta-analyses were the lack of information of registered protocols (110 studies, 90.16%). Thus, we conducted a sensitivity analysis, which did not consider the item of a registered protocol, to re-analyze the methodological quality of eligible studies. The results of sensitivity analysis showed that the AMSTAR 2 rating was re-determined as high in 14 studies (11.48%), moderate in 37 studies (30.33%), and low (35 studies, 28.69%) or critically low (36 studies, 29.51%) in 71 studies (see Additional file 5: Table S5).

### Quantitative analysis on 40 food groups, foods, and beverages

**Fig. 2:**
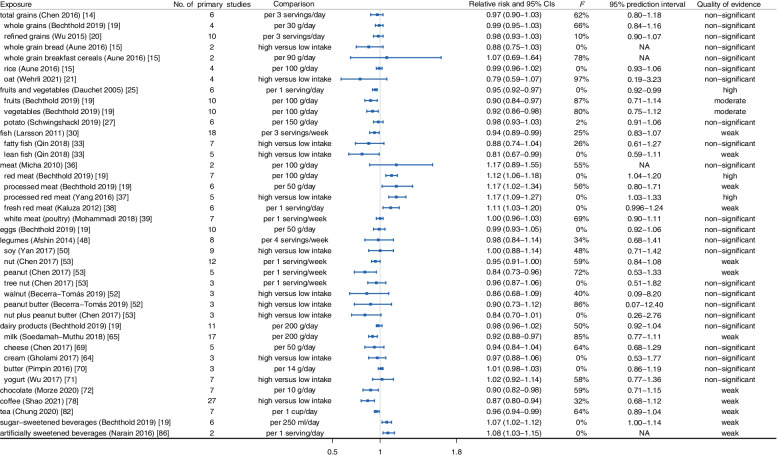
Summary relative risk with 95% CI, 95% PI, *I*^*2*^, and quality of evidence for associations between food groups, foods, and beverages and occurrence of stroke

As shown in Fig. 2, the summary effect size with its corresponding 95% CI was calculated to report the associations between food groups, foods, beverages, and stroke risk. First of all, we observed protective evidence for a dose-response relationship between the consumption of fruits (*RR*: 0.90, 95% *CI*: 0.84–0.97) [19], vegetables (*RR*: 0.92, 95% *CI*: 0.86–0.98) [19], fish (*HR*: 0.94, 95% *CI*: 0.89–0.99) [30], and chocolate (*RR*: 0.90, 95% *CI*: 0.82–0.98) [72] and the risk of stroke. Conversely, the consumption of red meat increased the incidence of stroke with evidence of a non-linear dose-response relationships involving 341,767 participants (*RR*: 1.12, 95% *CI*: 1.06–1.18) [19]. Besides, no clear dose-response associations were shown between the consumption of total grain foods (*RR*: 0.97, 95% *CI*: 0.90–1.03) [16], eggs (*RR*: 0.99, 95% *CI*: 0.93–1.05) [19], legumes (*RR*: 0.98, 95% *CI*: 0.84–1.14) [48], and dairy products (*RR*: 0.98, 95% *CI*: 0.96–1.02) [19] and stroke susceptibility.

**Fig. 3:**
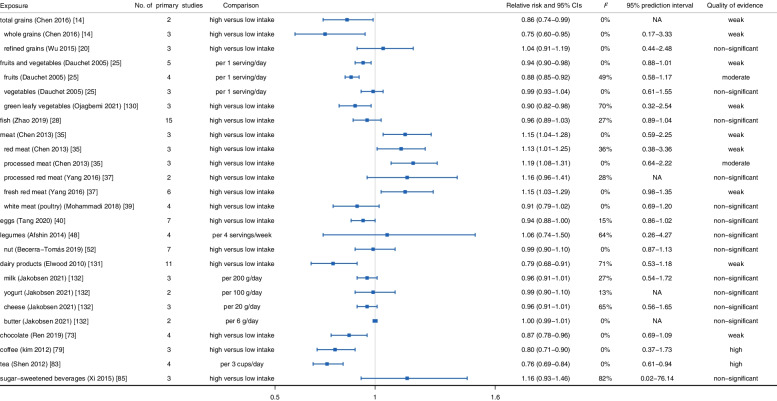
Summary relative risk with 95% CI, 95% PI, *I*^*2*^, and quality of evidence for associations between food groups, foods, and beverages and occurrence of ischemic stroke

**Fig. 4:**
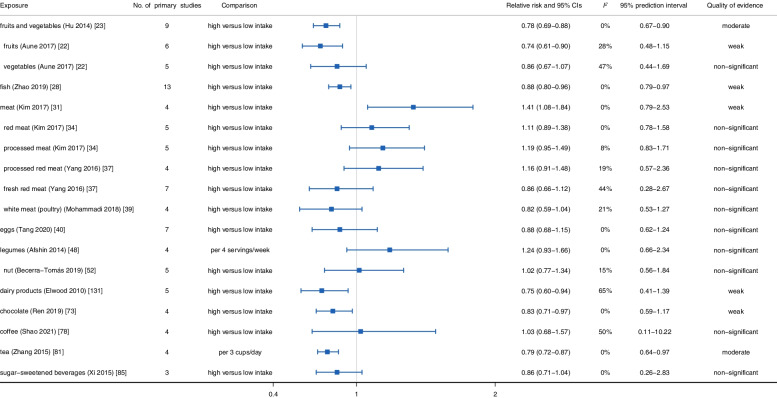
Summary relative risk with 95% CI, 95% PI, *I*^*2*^, and quality of evidence for associations between food groups, foods, and beverages and occurrence of hemorrhagic stroke

Next, we conducted a stratified evaluation according to the type of stroke. For ischemic stroke, the results of meta-analyses indicated that consumption of grain foods (*RR*: 0.86, 95% *CI*: 0.74–0.99) [14], fruits and vegetables (*RR*: 0.94, 95% *CI*: 0.90–0.98) [25], dairy products (*RR*: 0.79, 95% *CI*: 0.68–0.91) [131], and chocolate (*RR*: 0.87, 95% *CI*: 0.78–0.96) had a protective effect on ischemic stroke [73], while the consumption of meat increased the risk of ischemic stroke (*RR*: 1.15, 95% *CI*: 1.04–1.28) [35, 37, 39]. Besides, the consumption of fish (*HR*: 0.96, 95% *CI*: 0.89–1.03) [28], eggs (*RR*: 0.94, 95% *CI*: 0.88–1.00) [40], and legumes (*RR*: 1.06, 95% *CI*: 0.74–1.50) [48] was not related to the risk of ischemic stroke (Fig. 3). Regarding hemorrhagic stroke, the reduction of hemorrhagic stroke risk was related to the consumption of fruits and vegetables (*RR*: 0.78, 95% *CI*: 0.69–0.88) [23], fish (*HR*: 0.88, 95% *CI*: 0.80–0.96) [28], dairy products (*RR*: 0.75, 95% *CI*: 0.60–0.94) [131], and chocolate (*RR*: 0.83, 95% *CI*: 0.71–0.97) [73] and the increased risk of hemorrhagic stroke was associated with meat consumption (*RR*: 1.41, 95% *CI*: 1.08–1.84) [34]. In addition, no associations were observed between eggs (*RR*: 0.88, 95% *CI*: 0.68–1.15) [40] and legumes (*RR*: 1.24, 95% *CI*: 0.93–1.66) [48] and hemorrhagic stroke occurrence (Fig. 4).

Lastly, for beverages, people with high consumption of coffee were protected from subsequent stroke (*RR*: 0.87, 95% *CI*: 0.80–0.94) and ischemic stroke (*RR*: 0.80, 95% *CI*: 0.71–0.90), but not from hemorrhagic stroke (*RR*: 1.03, 95% *CI*: 0.68–1.57) [78, 79]. Additionally, dose-response evidence suggested that tea consumption (per cup per day) protected against stroke, ischemic stroke, and hemorrhagic stroke (stroke: *RR*: 0.96, 95% *CI*: 0.94-0.99; ischemic stroke: *RR*: 0.76, 95% *CI*: 0.69–0.84; hemorrhagic stroke: *RR*: 0.79, 95% *CI*: 0.72–0.87) [81–83]. Conversely, evidence from meta-analyses of prospective observational studies noted that sugar-sweetened beverage consumption increased the risk of stroke (*RR*: 1.07, 95% *CI*: 1.02–1.12), but not ischemic stroke (*RR*: 1.16, 95% *CI*: 0.93–1.46) or hemorrhagic stroke (*RR*: 0.86, 95%*CI*: 0.71–1.04) [19, 85] (Figs. 2, 3, and 4).

### Quantitative analysis on 31 food nutrients

#### Macronutrients

**Fig. 5:**
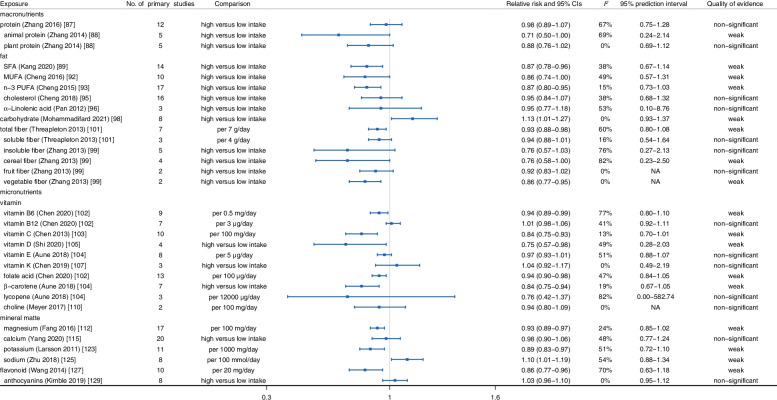
Summary relative risk with 95% CI, 95% PI, *I*^*2*^, and quality of evidence for associations between food nutrients and occurrence of stroke

As shown in Fig. 5, the associations between macronutrients and incidence of stroke were evaluated using summary effect size with its corresponding 95% CI. Among them, long-chain n-3 polyunsaturated fatty acid (n-3 PUFA) (*RR*: 0.87, 95% *CI*: 0.80–0.95) [93], saturated fat (SFA) (*RR*: 0.87, 95% *CI*: 0.78–0.96) [89], monounsaturated fatty acid (MUFA) (*RR*: 0.86, 95% *CI*: 0.74–1.00) [92], and dietary fiber (*RR*: 0.93, 95% *CI*: 0.88–0.98) [101] were associated with decreased incidence of stroke in meta-analyses comparing high versus low intake or dose-response meta-analyses, respectively, while a meta-analysis of 8 cohort studies involving 423,049 participants found high carbohydrate intake increased the risk of stroke (*RR*: 1.13, 95% *CI*: 1.01–1.27) [98]. Moreover, no evidence illustrated that high dietary protein and cholesterol were linked to the susceptibility of stroke (protein: *RR*: 0.98, 95% *CI*: 0.89–1.07; cholesterol: *RR*: 0.95, 95% *CI*: 0.84–1.07) [87, 95].

**Fig. 6:**
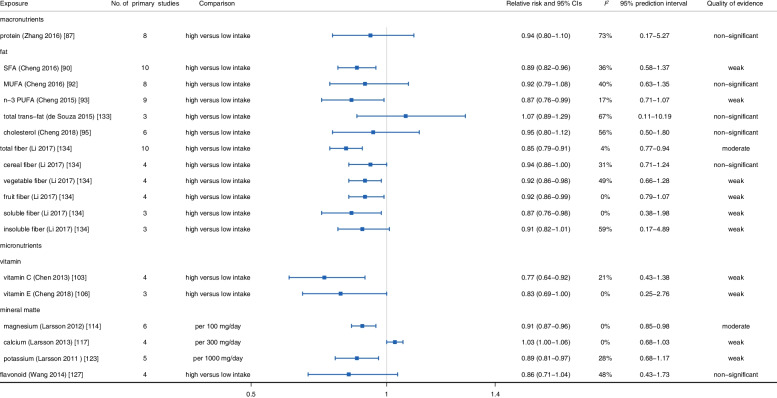
Summary relative risk with 95% CI, 95% PI, *I*^*2*^, and quality of evidence for associations between food nutrients and occurrence of ischemic stroke

**Fig. 7:**
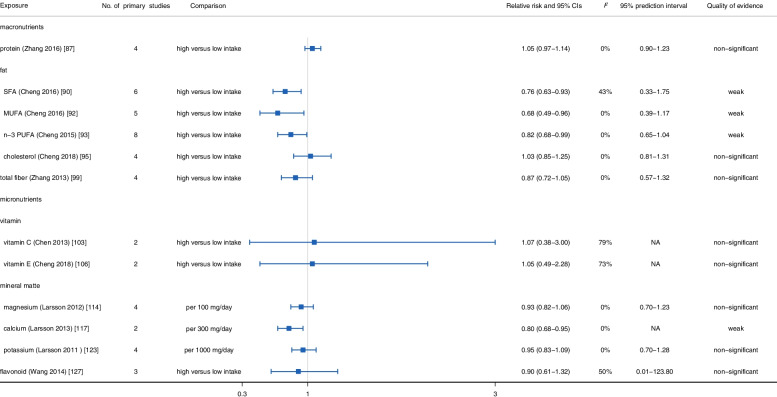
Summary relative risk with 95% CI, 95% PI, *I*^*2*^, and quality of evidence for associations between food nutrients and occurrence of hemorrhagic stroke

In further stratified evaluation based on stroke type (Figs. 6 and 7), we observed that n-3 PUFA and SFA intake could significantly reduce the risk of ischemic stroke (n-3 PUFA: *RR*: 0.87, 95% *CI*: 0.76–0.99; SFA: *RR*: 0.89, 95% *CI*: 0.82–0.96) and hemorrhagic stroke (n-3 PUFA: *RR*: 0.82, 95% *CI*: 0.68–0.99; SFA: *RR*: 0.76, 95% *CI*: 0.63–0.93) [90, 93]. Besides, the intake of dietary fiber had a significant protective effect on ischemic stroke (*RR*: 0.85, 95% *CI*: 0.79–0.91), while failed to reach significance in hemorrhagic stroke (*RR*: 0.87, 95% *CI*: 0.72–1.05) [99, 134]. In addition, no statistically significant evidence was found to indicate the associations between dietary protein and cholesterol intake and ischemic stroke (protein: *RR*: 0.94, 95% *CI*: 0.80–1.10; cholesterol: *RR*: 0.95, 95% *CI*: 0.80–1.12) and hemorrhagic stroke (protein: *RR*: 1.05, 95% *CI*: 0.97–1.14; cholesterol: *RR*: 1.03, 95% *CI*: 0.85–1.25) [87, 95].

#### Micronutrients

According to the meta-analyses of prospective observational studies, several dietary micronutrients, including vitamins, minerals, and flavonoids, were associated with stroke risk. As displayed in Fig. 5, dietary intake of vitamin B6 (*RR*: 0.94, 95% *CI*: 0.89–0.99) [102], folic acid (*RR*: 0.94, 95% *CI*: 0.90–0.98) [102], vitamin C (*RR*: 0.84, 95% *CI*: 0.75–0.93) [103], β-carotene (*RR*: 0.84, 95% *CI*: 0.75–0.94) [104], vitamin D (*RR*: 0.75, 95% *CI*: 0.57–0.98) [105], magnesium (*RR*: 0.93, 95% *CI*: 0.89–0.97) [112], potassium (*RR*: 0.89, 95% *CI*: 0.83–0.97) [123], and flavonoid (*RR*: 0.86, 95% *CI*: 0.77–0.96) [127] had a significant impact on decreasing the occurrence of stroke. Conversely, sodium intake had a significant effect on increasing stroke risk (*RR*: 1.10, 95% *CI*: 1.01–1.19) with evidence of a linear dose-response relationship [125]. Additionally, no clear associations were observed between dietary vitamin B12 (*RR*: 1.01, 95% *CI*: 0.98–1.06) [102], vitamin E (*RR*: 0.97, 95% *CI*: 0.93–1.01) [104], vitamin K (*HR*: 1.04, 95% *CI*: 0.92–1.17) [107], lycopene (*RR*: 0.76, 95% *CI*: 0.42–1.37) [104], choline (*RR*: 0.94, 95%*CI*: 0.80–1.09) [110], and calcium (*RR*: 0.98, 95% *CI*: 0.90–1.06) [115] intake and the incidence of stroke.

With regard to subgroup evaluation, we observed dietary vitamin C (*RR*: 0.77, 95% *CI*: 0.64–0.92) [103], vitamin E (*RR*: 0.83, 95% *CI*: 0.69–1.00) [106], magnesium (*RR*: 0.91, 95% *CI*: 0.87–0.96) [114], and potassium (*RR*: 0.89, 95% *CI*: 0.81–0.97) [123] intake protected against ischemic stroke, but did not reach statistical significance in hemorrhagic stroke (vitamin C: *RR*: 1.07, 95% *CI*: 0.38–3.00; vitamin E: *RR*: 1.05, 95% *CI*: 0.49–2.28; magnesium: *RR*: 0.93, 95% *CI*: 0.82–1.06; potassium: *RR*: 0.95, 95% *CI*: 0.83–1.09). Besides, flavonoid intake was not related to ischemic stroke (*RR*: 0.86, 95% *CI*: 0.71–1.04) and hemorrhagic stroke (*RR*: 0.90, 95% *CI*: 0.61–1.32) [127] (Figs. 6 and 7).

### Heterogeneity between primary studies, 95% prediction intervals, and small-study effects

We reported the assessment of the level of heterogeneity, 95% PI, and the presence of small-study effects. Firstly, our results appraised the heterogeneity between primary studies using the *I*^*2*^ value. Most studies (57.75%, 41/71) had *I*^*2*^*≤*50.00%, implying low heterogeneity between primary studies, while 30 associations (42.25%) showed substantial heterogeneity (*I*^*2*^ *>*50.0%), indicating that the difference of risk estimates between primary studies may not only be due to random error. Next, the 95% PIs of 4 associations excluded the null value—that was the consumption of fruits and vegetables, red meat, processed red meat, and sugar-sweetened beverages. The remaining meta-analyses of dietary factors had 95% PIs which contained the null value, suggesting that, although on average some dietary factors were associated with stroke risk, this may not always be the case in certain settings. Lastly, based on Egger’s test and the funnel plot (see Additional file 6: Fig. S1-S16), the 9 associations (14.06%) showed the presence of small-study effects and potential publication bias (*P*<0.10). Among them, 7 dietary factors were indicated in the dose-response meta-analyses involving legumes, nut, milk, chocolate, dietary fiber, vitamin B6, and flavonoids, and the other two factors, soy and coffee, were indicated in the meta-analyses comparing high versus low consumption.

### Strength of epidemiologic evidence

Our study assessed the strength of epidemiologic evidence for the association between dietary factors and stroke risk. Among them, moderate/high certainty of evidence was found for red meat, especially processed red meat consumption, which was associated with an increased incidence of stroke, as well as for the intake of fruits and vegetables, which showed an association with decreased incidence of stroke. Additionally, 5 other risk factors and 24 protective factors were confirmed as statistically significant, but the strength of the evidence was weak. Lastly, the included studies did not observe a significant effect of other 37 dietary factors on stroke (*P*>0.05).

With regard to stratification of stroke subtypes, 25 dietary factors were found to be significantly associated with ischemic stroke, among which the credibility of 6 dietary factors, including fruits, processed meat, coffee, tea, magnesium, and dietary fiber consumption, was moderate/high, and the other 19 dietary factors were weak. As for hemorrhagic stroke, two protective dietary factors (fruits and vegetables, and tea consumption) showed high/moderate strength of evidence and the remaining 9 dietary factors showed weak evidence.

## Discussion

### Principal findings

In our umbrella review, a total of 122 eligible meta-analyses were included to assess the impact of 71 dietary factors on stroke, including 40 foods, food groups, and beverages and 31 macronutrients and micronutrients. After assessing the credibility of all included meta-analyses using stringent criteria, the evidence strength for fruits, vegetables, and red meat was considered as high/moderate, indicating that they may have an important impact on stroke prevention. Among them, the intake of fruits and vegetables was observed to reduce the risk of stroke, while the consumption of red meat, especially processed red meat, was considered to increase the risk.

### Possible explanations

Our umbrella review indicated that high consumption of fruits and vegetables was beneficial to the general population for preventing stroke. This protective effect can be attributed to the various nutrients contained in fruits and vegetables, including vitamin C, potassium, dietary fiber, and flavonoids [22]. First, vitamin C, a powerful water-soluble antioxidant, has been suggested to inhibit low-density lipoprotein peroxidation and smooth muscle hyperplasia/hypertrophy, thereby retarding the formation of atherosclerosis [103, 136, 137]. Second, potassium has been found to have an impact on the development of stroke. Increased potassium levels would relax blood vessels and inhibit excessive activation of platelets. Moreover, a high-potassium diet could significantly delay the development of vascular damage by restraining the production of reactive oxygen species [122, 138]. Third, the consumption of dietary fiber can slow down gastric emptying, promote satiety, reduce absorption of food, and thus reduce body overweight and blood lipid levels [101, 139, 140]. As secondary metabolites of polyphenols, flavonoids can inhibit LDL oxidation and vascular inflammation and play an important role in protecting endothelial function [141–143].

Additionally, it is also biologically reasonable that high consumption of red meat could increase the risk of stroke. First of all, high red meat intake could increase the circulating levels of LDL-C and triglycerides, which might cause atherosclerotic plaques, interrupt blood flow to the brain, and lead to stroke occurrence [34]. Then, heme iron, mainly derived from red meat, is a redox active substance that could promote the production of oxygen free radicals, leading to LDL-C peroxidation and subsequent vascular inflammation and damage [144–146]. Moreover, processed meat usually contains high levels of sodium and nitrite preservatives [124]. High sodium levels could reduce arterial compliance, cause vascular stiffness, and thus have a negative impact on subsequent high blood pressure and stroke [147, 148]. The cytotoxicity of nitrite preservatives can induce vascular endothelium damage and apoptosis, which is a critical driving factor for endothelial dysfunction [149].

### Subgroup evaluation

Regarding ischemic stroke, the evidence of high/moderate certainty indicated that the intake of coffee, tea, magnesium, fruits, dietary fiber, and processed meat was associated with ischemic stroke risk. From a biological point of view, caffeine, a famous ingredient in coffee, plays a vital role in reducing oxidation stress and inflammatory response and delaying atherosclerosis progression [150]. Moreover, the chlorogenic acid contained in coffee can regulate the body’s glucose and lipid metabolism and inhibit the activation of platelets [78]. As for tea, flavonoids in tea can induce vasodilation and improve cerebral blood perfusion by activating nitric oxide [151, 152]. Meanwhile, tea contains a high concentration of theanine, which can pass through the blood-brain barrier and reduce glutamate-related vascular endothelial damage [84]. Regarding micronutrients, magnesium has been shown to be associated with ischemic stroke, which could be explained by the following reasons. As a natural calcium antagonist, magnesium could inhabit the influx of glutamate and calcium cations and eliminate the cytotoxicity of calcium cations [111]. Moreover, a previous study showed that magnesium deficiency was related to vascular dysfunction and platelet-dependent thrombosis [153]. Besides, magnesium intake also plays a vital role in lowering blood sugar and blood pressure levels [154].

With regard to hemorrhagic stroke, we found more associations for ischemic stroke than hemorrhagic stroke. The possible reasons may be as follows: the etiology of ischemic stroke, including oxidative stress, free radical production, lipid peroxidation, and vascular inflammation and atherosclerosis, is more closely related to nutritional factors [155, 156]. More importantly, the incidence of ischemic stroke is much higher than that of hemorrhagic stroke, so it receives more attention from researchers. Thus, more original studies should be performed to investigate the relationship between dietary factors and hemorrhagic stroke.

### Strengths and limitations

To the best of our knowledge, our umbrella review was the first to systematically collect and evaluate all published meta-analyses and summarize the evidence on the role of dietary factors in preventing stroke. We have included only meta-analyses focusing on prospective observational studies, which collected exposure information before stroke diagnosis and reduced recall bias compared to retrospective studies. Meanwhile, robust criteria were adopted to assess the methodologic quality and evidence strength of eligible meta-analyses. Moreover, we highlighted the dose-response relationship, subgroup evaluation, sensitivity analysis, and biological plausibility to obtain a more comprehensive and accurate conclusion for each dietary factor.

Several limitations of this umbrella review should also be recognized. First, the individual observational study may have a different definition and measurement method for exposure comparison, which makes it impossible to determine the exact comparison for the included meta-analyses. Second, within an observational design, the original studies in the meta-analysis were prone to confounding bias. Thus, some known confounders were adjusted for in most of the original studies. Moreover, we extracted the fully adjusted effect estimates for further analysis. However, regarding the differences in the adjustment models in the original studies, residual confoundings cannot be completely ruled out for some summary effect estimates, thereby distorting true effect sizes. Third, for dietary factors in our umbrella review, we systematically selected 41 dose-response meta-analyses in the main analysis. However, information of linearity was only available for 76% (31/41) of all available dose-response meta-analyses, with 16% (5/31) showing a non-linear dose-response relationship. Thus, further investigation is required to provide the information of linearity and determine the optimal cut-off point to arrive at a recommendation. Lastly, most included meta-analyses were of low quality due to a lack of protocol. Thus, more widespread adoption of reporting guidelines, such as MOOSE (Meta-analysis Of Observational Studies in Epidemiology) and QUORUM (Quality of Reporting of Meta-analyses), may help to improve the quality of future meta-analyses [157].

## Conclusions

In conclusion, we have reported the most comprehensive evaluation of the relationship between dietary factors and stroke risk and found that 34 dietary factors were associated with stroke susceptibility. After using strict criteria to assess the strength of epidemiologic evidence, a series of dietary factors showed high/moderate-strength evidence, including red meat, especially processed red meat, fruits, and vegetables. Our results may provide new insights for implementing the best strategies for stroke prevention.

## Supplementary Information

**Additional file 1: Table S1.** List of excluded full-text articles.

**Additional file 2: Table S2.** Characteristics of included meta-analyses evaluating associations between food groups, foods as well as beverages and stroke risk.

**Additional file 3: Table S3.** Characteristics of included meta-analyses evaluating associations between food nutrients and stroke risk.

**Additional file 4: Table S4.** Quality assessment of included meta-analyses using AMSTAR 2.

**Additional file 5: Table S5.** Quality assessment of included meta-analyses using AMSTAR 2, without considering item 2 (sensitivity analysis).

**Additional file 6: Fig. S1** Funnel plots for the association between A) total grains, B) whole grain, C) refined grain, D) whole grain bread, E) whole grain breakfast cereals, F) rice, G) oat and incidence of stroke. **Fig. S2** Funnel plots for the association between A) fruits and vegetables, B) fruits, C) vegetables, D) potato and incidence of stroke. **Fig. S3** Funnel plots for the association between A) fish, B) fatty fish, C) lean fish and incidence of stroke. **Fig. S4** Funnel plots for the association between A) meat, B) red meat, C) processed meat, D) processed red meat, E) fresh red meat, F) white meat (poultry) and incidence of stroke. **Fig. S5** Funnel plots for the association between eggs and incidence of stroke. **Fig. S6** Funnel plots for the association between A) legumes, B) soy, C) nut, D) peanuts, E) tree nuts, F) walnuts, G) peanut butter, H) nut plus peanut butter and incidence of stroke. **Fig. S7** Funnel plots for the association between A) dairy products, B) milk, C) cheese, D) cream, E) butter, F) yogurt and incidence of stroke. **Fig. S8** Funnel plots for the association between chocolate and incidence of stroke. **Fig. S9** Funnel plots for the association between A) coffee, B) tea, C) sugar-sweetened beverages, D) artificially sweetened beverage and incidence of stroke. **Fig. S10** Funnel plots for the association between A) protein, B) animal protein, C) plant protein and incidence of stroke. **Fig. S11** Funnel plots for the association between A) saturated fat, B) MUFA, C) n-3 PUFA, D) cholesterol, E) α-linolenic acid and incidence of stroke. **Fig. S12** Funnel plots for the association between carbohydrate and incidence of stroke. **Fig. S13** Funnel plots for the association between A) total fiber, B) soluble dietary fiber, C) insoluble dietary fiber, D) cereal fiber, E) fruit fiber, F) vegetable fiber and incidence of stroke. **Fig. S14** Funnel plots for the association between A) vitamin B6, B) vitamin B12, C) vitamin C, D) vitamin D, E) vitamin E, F) vitamin K, G) folate acid, H) β-carotene, I) lycopene, J) dietary choline and incidence of stroke. **Fig. S15** Funnel plots for the association between A) magnesium, B) calcium, C) potassium, D) sodium and incidence of stroke. **Fig. S16** Funnel plots for the association between A) flavonoid, B) anthocyanins and incidence of stroke.

## Data Availability

The datasets used and/or analyzed during the current study are available from the corresponding author on reasonable request.

## Abbreviations

AMSTAR: A MeaSurement Tool to Assess systematic Reviews
CIs: Confidence intervals
LDL: Low-density lipoprotein
MOOSE: Meta-analysis Of Observational Studies in Epidemiology
MUFA: Monounsaturated fatty acid
n-3 PUFA: n-3 polyunsaturated fatty acid
PIs: Prediction intervals
PRISMA: Preferred Reporting Items for Systematic Reviews and Meta-Analyses
QUORUM: Quality of Reporting of Meta-analyses
SFA: Saturated fat
