# Supplementary material for: Role of diet in stroke incidence: an umbrella review of meta-analyses of prospective observational studies
Source: BMC Med. 2022 May 24;20:194. doi: 10.1186/s12916-022-02381-6 (PMC9128224; doi:10.1186/s12916-022-02381-6)
Supplement: Supplementary file 1 — Additional file 1: Table S1. List of excluded full-text articles. [file 12916_2022_2381_MOESM1_ESM.docx]

**Table S1. List of excluded full-text reference.**

| **Reasons for exclusion** | **Number** | **Reference** |
| --- | --- | --- |
| Letters, conference abstracts and reviews | 157 | [1-157] |
| Not focus on the risk of stroke or its subgroup | 41 | [158-198] |
| Not dietary factors | 61 | [199-259] |
| Not meta-analysis of prospective observational studies | 50 | [260-309] |
| Non-English publication | 3 | [310-312] |

**Reference:**

1. Tang H, Cao Y, Yang X, Zhang Y: **Corrigendum: Egg Consumption and Stroke Risk: A Systematic Review and Dose-Response Meta-Analysis of Prospective Studies**. *Frontiers in nutrition* 2020, **7**:618715.

2. Bhupathi V, Mazariegos M, Cruz Rodriguez JB, Deoker A: **Dairy Intake and Risk of Cardiovascular Disease**. *Current cardiology reports* 2020, **22**(3):11.

3. Astrup A, Magkos F, Bier DM, Brenna JT, de Oliveira Otto MC, Hill JO, King JC, Mente A, Ordovas JM, Volek JS *et al*: **Saturated Fats and Health: A Reassessment and Proposal for Food-Based Recommendations: JACC State-of-the-Art Review**. *Journal of the American College of Cardiology* 2020, **76**(7):844-857.

4. Gaesser GA: **Perspective: Refined Grains and Health: Genuine Risk, or Guilt by Association?** *Advances in nutrition (Bethesda, Md)* 2019, **10**(3):361-371.

5. Aune D: **Plant Foods, Antioxidant Biomarkers, and the Risk of Cardiovascular Disease, Cancer, and Mortality: A Review of the Evidence**. *Advances in nutrition (Bethesda, Md)* 2019, **10**(Suppl_4):S404-s421.

6. Yu E, Hu FB: **Dairy Products, Dairy Fatty Acids, and the Prevention of Cardiometabolic Disease: a Review of Recent Evidence**. *Current atherosclerosis reports* 2018, **20**(5):24.

7. Rosique-Esteban N, Guasch-Ferré M, Hernández-Alonso P, Salas-Salvadó J: **Dietary Magnesium and Cardiovascular Disease: A Review with Emphasis in Epidemiological Studies**. *Nutrients* 2018, **10**(2).

8. Nielsen FH: **Dietary Magnesium and Chronic Disease**. *Advances in chronic kidney disease* 2018, **25**(3):230-235.

9. Gille D, Schmid A, Walther B, Vergères G: **Fermented Food and Non-Communicable Chronic Diseases: A Review**. *Nutrients* 2018, **10**(4).

10. Cheng P, Pan J, Xia J, Huang W, Bai S, Zhu X, Shao W, Wang H, Xie P: **Correction: Dietary cholesterol intake and stroke risk: a meta-analysis**. *Oncotarget* 2018, **9**(45):27907.

11. Wolk A: **Potential health hazards of eating red meat**. *Journal of internal medicine* 2017, **281**(2):106-122.

12. Micha R, Peñalvo JL, Cudhea F, Imamura F, Rehm CD, Mozaffarian D: **Association Between Dietary Factors and Mortality From Heart Disease, Stroke, and Type 2 Diabetes in the United States**. *Jama* 2017, **317**(9):912-924.

13. Hurtado-Torres GF: **Comment on: Chocolate Consumption and Risk of Coronary Heart Disease, Stroke, and Diabetes: A Meta-analysis of prospective Studies, Nutrients 2017, 9, 688**. *Nutrients* 2017, **9**(8).

14. Balk EM, Lichtenstein AH: **Omega-3 Fatty Acids and Cardiovascular Disease: Summary of the 2016 Agency of Healthcare Research and Quality Evidence Review**. *Nutrients* 2017, **9**(8).

15. Thorning TK, Raben A, Tholstrup T, Soedamah-Muthu SS, Givens I, Astrup A: **Milk and dairy products: good or bad for human health? An assessment of the totality of scientific evidence**. *Food & nutrition research* 2016, **60**:32527.

16. **Erratum for Adebamowo et al. Association between intakes of magnesium, potassium, and calcium and risk of stroke: 2 cohorts of US women and updated meta-analyses. Am J Clin Nutr 2015;101:1269-77**. *The American journal of clinical nutrition* 2015, **102**(4):981-982.

17. Whelton PK, He J: **Health effects of sodium and potassium in humans**. *Current opinion in lipidology* 2014, **25**(1):75-79.

18. Sanders TA: **Protective effects of dietary PUFA against chronic disease: evidence from epidemiological studies and intervention trials**. *The Proceedings of the Nutrition Society* 2014, **73**(1):73-79.

19. Ness AR, Powles JW: **Fruit and vegetables, and cardiovascular disease: a review**. *International journal of epidemiology* 1997, **26**(1):1-13.

20. Gorelick PB, Sacco RL, Smith DB, Alberts M, Mustone-Alexander L, Rader D, Ross JL, Raps E, Ozer MN, Brass LM *et al*: **Prevention of a first stroke: a review of guidelines and a multidisciplinary consensus statement from the National Stroke Association**. *Jama* 1999, **281**(12):1112-1120.

21. Dagenais GR, Marchioli R, Yusuf S, Tognoni G: **Beta-carotene, vitamin C, and vitamin E and cardiovascular diseases**. *Current cardiology reports* 2000, **2**(4):293-299.

22. McCarty MF: **Scavenging of peroxynitrite-derived radicals by flavonoids may support endothelial NO synthase activity, contributing to the vascular protection associated with high fruit and vegetable intakes**. *Medical hypotheses* 2008, **70**(1):170-181.

23. Perez-Vizcaino F, Duarte J: **Flavonols and cardiovascular disease**. *Molecular aspects of medicine* 2010, **31**(6):478-494.

24. Companys J, Pedret A, Valls RM, Solà R, Pascual V: **Fermented dairy foods rich in probiotics and cardiometabolic risk factors: a narrative review from prospective cohort studies**. *Critical reviews in food science and nutrition* 2020:1-10.

25. Butt MS, Sultan MT: **Coffee and its consumption: benefits and risks**. *Critical reviews in food science and nutrition* 2011, **51**(4):363-373.

26. Danik JS, Manson JE: **Vitamin d and cardiovascular disease**. *Current treatment options in cardiovascular medicine* 2012, **14**(4):414-424.

27. Foroughi M, Akhavanzanjani M, Maghsoudi Z, Ghiasvand R, Khorvash F, Askari G: **Stroke and nutrition: a review of studies**. *International journal of preventive medicine* 2013, **4**(Suppl 2):S165-179.

28. Lorente-Cebrián S, Costa AG, Navas-Carretero S, Zabala M, Martínez JA, Moreno-Aliaga MJ: **Role of omega-3 fatty acids in obesity, metabolic syndrome, and cardiovascular diseases: a review of the evidence**. *Journal of physiology and biochemistry* 2013, **69**(3):633-651.

29. Rebello SA, van Dam RM: **Coffee consumption and cardiovascular health: getting to the heart of the matter**. *Current cardiology reports* 2013, **15**(10):403.

30. Sanders TA: **Reappraisal of SFA and cardiovascular risk**. *The Proceedings of the Nutrition Society* 2013, **72**(4):390-398.

31. Woodside JV, Young IS, McKinley MC: **Fruit and vegetable intake and risk of cardiovascular disease**. *The Proceedings of the Nutrition Society* 2013, **72**(4):399-406.

32. Berciano S, Ordovás JM: **Nutrition and cardiovascular health**. *Revista espanola de cardiologia (English ed)* 2014, **67**(9):738-747.

33. Kromhout D, de Goede J: **Update on cardiometabolic health effects of ω-3 fatty acids**. *Current opinion in lipidology* 2014, **25**(1):85-90.

34. Reiner MF, Stivala S, Camici GG, Beer JH: **[The effects of Omega-3 fatty acids in clinical medicine]**. *Praxis* 2014, **103**(6):329-335.

35. Hu Y, Sun JY, Zhang Y, Zhang H, Gao S, Wang T, Han Z, Wang L, Sun BL, Liu G: **rs1990622 variant associates with Alzheimer's disease and regulates TMEM106B expression in human brain tissues**. *BMC medicine* 2021, **19**(1):11.

36. **Correction: Association of magnesium intake with type 2 diabetes and total stroke: an updated systematic review and meta-analysis**. *BMJ open* 2020, **10**(4):e032240corr032241.

37. Rezaei S, Gholami A: **Letter to the editor about "Diet and primary prevention of stroke: Systematic review and dietary recommendations by the ad hoc Working Group of the Italian Society of Human Nutrition"**. *Nutrition, metabolism, and cardiovascular diseases : NMCD* 2018, **28**(7):775-776.

38. Yuan S, Lu J: **Reply: "Comment on: Chocolate Consumption and Risk of Coronary Heart Disease, Stroke, and Diabetes: A Meta-Analysis of Prospective Studies, Nutrients 2017, 9, 688"**. *Nutrients* 2017, **9**(8).

39. Tikhonoff V, Palatini P, Casiglia E: **Letter by Tikhonoff et al regarding article, "dietary fiber intake and risk of first stroke: a systematic review and meta-analysis"**. *Stroke* 2013, **44**(9):e109.

40. Threapleton DE, Greenwood DC, Burley VJ: **Response to letter regarding article, "dietary fiber intake and risk of first stroke: a systematic review and meta-analysis"**. *Stroke* 2013, **44**(9):e110.

41. Bryan NS: **Letter by Bryan regarding article, "red and processed meat consumption and risk of incident coronary heart disease, stroke, and diabetes mellitus: a systematic review and meta-analysis"**. *Circulation* 2011, **123**(3):e16; author reply e17.

42. Upadhaya S, Madala S, Tiwari K: **Regular Intake of Pepper Decreases Cardiovascular Mortality: A Meta-Analysis**. *Circulation* 2020, **142**(SUPPL 3).

43. Tran D, Nguyen C, Nguyen T: **PCV5 Cost-Effectiveness Analysis of Apixaban in Treatment of Atrial Fibrillation: A Systematic Review**. *Value in Health Regional Issues* 2020, **22**:S26-S27.

44. Tariq M, Bhanu G, Chhabra A: **Excessive caffeine intake from energy supplements as a cause of ischemic stroke in young patients**. *Annals of Neurology* 2020, **88**(SUPPL 25):S90.

45. Rücker V, Montellano F, Neuhauser H, Mensink G, Neugebauer H, Haeusler KG, Störk S, Kolominsky-Rabas P, Hermanek P, Misselwitz B *et al*: **Explaining the decline in stroke mortality due to changes in treatment and risk factors in Germany: The German impact stroke model**. *International Journal of Stroke* 2020, **15**(1 SUPPL):51.

46. Park SG: **Associations Between Coffee Consumption and the Risk of Stroke: A Systematic Review and Meta-Analysis**. *Metabolism: Clinical and Experimental* 2020, **104**.

47. Olmastroni E, Casula M, Gazzotti M, Galimberti F, Tragni E, Zambon A, Catapano AL: **Evaluation of the effect of omega-3 polyunsaturated fatty acids supplementation on cardiovascular outcomes: An updated meta-analysis of randomized controlled trials**. *Pharmacoepidemiology and Drug Safety* 2020, **29**(SUPPL 3):53-54.

48. Morze J, Danielewicz A, Rynkiewicz A, Przybylowicz K: **Breakfast skipping and risk of cardiovascular disease: A systematic review and meta-analysis of prospective studies**. *Proceedings of the Nutrition Society* 2020, **79**(OCE2).

49. Mazidi M, Shekoohi N, Katsiki N, Banach M: **Omega-6 fatty acids and risk of cardiovascular disease: Insight from systematic review and meta-analysis of randomized controlled trials and a mendelian randomization study**. *European Heart Journal* 2020, **41**(SUPPL 2):2836.

50. Love MF, Biondo-Wood GL, Wardell DW, Beauchamp JE: **Predictors of resilience among CVD patients**. *Circulation* 2020, **141**(SUPPL 1).

51. Limbachia J, Ajmeri M, Keating B, de Souza R, Anand S: **EFFECTS OF LIFESTYLE INTERVENTION ON CARDIOVASCULAR DISEASE RISK IN SOUTH ASIANS: A SYSTEMATIC REVIEW AND META-ANALYSIS**. *Canadian Journal of Cardiology* 2020, **36**(10):S90-S91.

52. Kaur M, Verma BR, Zhou L, Kaur S, Sammour Y, Lak HM, Xu B: **Impact of Chilli-pepper Intake on All-cause and Cardiovascular Mortality - A Systematic Review and Meta-analysis**. *Circulation* 2020, **142**(SUPPL 3).

53. Hernandez AV, Piscoya A, Marti KM, Marti KE, Pasupuleti V, Benites-Zapata VA, Roman YM: **Effect of mediterranean diets on cardiovascular risk factors and diseases in the primary prevention setting: A systematic review and meta-analysis of randomized controlled trials**. *European Heart Journal* 2020, **41**(SUPPL 2):2856.

54. Green BP, Wong E, Andrews S, Hampshire-Jones K, McKinnon S, Brooks C, McAdam R, Gray S, Vickers C, Blake Y *et al*: **Increased protein intake is associated with improved hand grip strength and quality of life in home enterally tube fed adults using a high-energy, high-protein feed**. *Clinical Nutrition ESPEN* 2020, **35**:208.

55. Bensaaud A, Seery S, Gibson I, Jones J, Flaherty G, McEvoy JW, Jordan F, Tawfick W, Sultan S: **Dietary approaches to stop hypertension (DASH) for the primary and secondary prevention of cardiovascular diseases: A protocol for a cochrane systematic review and meta-analysis**. *Atherosclerosis* 2020, **315**:e236.

56. Asfaw AA, Teshome MK, Cave B, Khouzam RN: **The fish oil dilemma-does it increase recurrence risk of ventricular arrythmia?** *Journal of Investigative Medicine* 2020, **68**(2):473.

57. Worrall L: **Three key clinical indicators of effective stroke care for people with aphasia**. *International Journal of Stroke* 2019, **14**(1):7.

58. Wang J, Lee-Bravatti MA, Raman G, Avendano EE, Johnson EJ: **Almond consumption in cardiovascular disease: Markov model and health economic evaluation**. *Circulation* 2019, **139**.

59. Van Der Weijde E, Saouti N, Bakker OJ, Heijmen RH: **Left Subclavian Artery Revascularization for Thoracic Aortic Stentgrafting: Single Center Experience in 101 Patients**. *European Journal of Vascular and Endovascular Surgery* 2019, **58**(6):e76-e77.

60. Mazidi M, Mikhailidis DP, Sattar N, Toth PP, Judd S, Blaha MJ, Hernandez AV, Banach M: **Association of types of dietary fats and all-cause and cause-specific mortality: A prospective cohort study and meta-analysis of prospective studies with 1,148,117 participants**. *European Heart Journal* 2019, **40**:7.

61. Mazidi M, Mikhailidis DP, Katsiki N, Pella D, Banach M: **Potato consumption is associated with total and cause-specific mortality: A population-based cohort study and pooling of prospective studies with 73,717 participants**. *European Heart Journal* 2019, **40**:3837.

62. Park JM, Cho YJ, Park TH: **Alcohol intake and risk of ischemic stroke : aa dose-response meta-analysis**. *European Stroke Journal* 2018, **3**(1):548.

63. Nudy M, Krakowksi G, Ghahramani M, Ruzieh M, Foy AJ: **Vitamin D supplementation, cardiac events, and stroke: A systematic review and meta-analysis**. *Circulation* 2018, **138**.

64. Mazidi M, Mikhailidis DP, Howard G, Graham I, Banach M: **Consumption of dairy product and its association with total and cause specific mortality-A population-based cohort study and meta-analysis**. *European Heart Journal* 2018, **39**:1115.

65. Mazidi M, Katsiki N, Mikhailidis DP, Banach M: **Low-carbohydrate diets and all-cause and cause-specific mortality: A population-based cohort study and pooling prospective studies**. *European Heart Journal* 2018, **39**:1112-1113.

66. Francini-Pesenti F: **Nutrition in the healthy population**. *Acta Myologica* 2018, **37**(1):39.

67. Su PJ, Afshin A: **The impact of diet low in fruits and vegetables on cardiovascular disease burden across 195 countries in 2015**. *Circulation* 2017, **135**.

68. Scrafford C, Schmier JK, Murphy MM, Barraj LM: **Healthcare costs and savings associated with increased dairy consumption in the United States**. *FASEB Journal* 2017, **31**(1).

69. Schoufour J, Voortman T, Kiefte-De Jong J, Franco O: **Adherence to the 2015 DUTCH dietary guidelines and its associations with mortality and incidence of non-communicable diseases in the rotterdam study**. *Annals of Nutrition and Metabolism* 2017, **71**:355-356.

70. Kim K, Hyeon J, Lee SA, Kwon SO, Lee H, Keum N, Lee JK, Park SM: **Total, red, processed, and white meat intake and stroke incidence and mortality: A systematic review and meta-analysis of cohort studies**. *European Stroke Journal* 2017, **2**(1):445.

71. Heseker H: **Evidence-based guidelines on fat and carbohydrate intake**. *Annals of Nutrition and Metabolism* 2017, **71**:88-89.

72. Cho YJ, Park TH, Park JM, Jang JE, Shin IS: **Alcohol intake and risk of stroke: A dose-response meta-analysis**. *Cerebrovascular Diseases* 2017, **44**:17.

73. Sy S, Peñalvo J, Abrahams-Gessel S, Alam S, Pandya A, Mozaffarian D, Gaziano TA: **Changes in food prices improve cardiovascular disease (CVD) outcomes**. *Circulation* 2016, **133**.

74. Salas-Salvadó J: **Dietary magnesium and cardiovascular diseases**. *Magnesium Research* 2016, **29**(3):64.

75. Saber H, Yakoob MY, Shi P, Longstreth W, Rimm EB, Lemaitre RN, Siscovick D, Rexrode K, Willett WC, Mozaffarian D: **Circulating phospholipid N-3 polyunsaturated fatty acids and incident atherothrombotic and cardioembolic ischemic stroke in 3 large us cohorts**. *Circulation* 2016, **133**.

76. Rexrode M: **Dietary magnesium and stroke Kathryn**. *Magnesium Research* 2016, **29**(3):64-65.

77. Narain A: **Soft drinks and sweetened beverages and the risk of cardiovascular disease and mortality: a systematic review and meta-analysis**. *Heart* 2016, **102**:A61.

78. Mostofsky E, Chahal HS, Mukamal KJ, Rimm EB, Mittleman MA: **Systematic review and meta-analysis of acute effects of alcohol consumption on risk of cardiovascular events**. *Circulation* 2016, **133**.

79. Marklund M, Chen TA, De Goede J, Imamura F, Laguzzi F, Prem K, Samieri C, Shi P, Virtanen J, Wennberg M *et al*: **Biomarkers of N-6 polyunsaturated fatty acids and CVD risk: A global pooling project of 19 cohort studies**. *Circulation* 2016, **133**.

80. Ivanov A, Patel T, Ho J, Gerber M, Chen A, Khan S, Heitner J, Brener S: **Effect of coffee or tea consumption on mortality and morbidity: A network metaanalysis of 58 studies**. *Journal of the American College of Cardiology* 2016, **67**(13):1919.

81. Eui Ju L, Shin S, Yoo J, Kim EY, Yanhua L, Hyun Jung K: **Effect of constitution-based diet therapy for lifestyle-related diseases in Korea medicine: A systematic review and meta-analysis**. *European Stroke Journal* 2016, **1**(1):559.

82. Dinu M, Pagliai G, Casini A, Sofi F: **Vegetarian, vegan diets and multiple health outcomes: A systematic review with meta-analysis of observational studies**. *European Heart Journal* 2016, **37**:549.

83. De Goede J, Geleijnse JM, Pan A, Gijsbers L, Soedamah-Muthu S: **Dairy consumption and risk of stroke: A systematic review and dose-response meta-analysis of prospective cohort studies**. *Circulation* 2016, **133**.

84. Clague-Baker N, Carpenter C, Robinson T, Hagenberg A, Drewry S, Singh S: **Attitudes and understanding of exercise and healthy lifestyles in people with mild to moderate sub-acute stroke**. *Physiotherapy* 2016, **102**:e9-e10.

85. Agarwal S: **Yoga and stroke: An evidence based review**. *Journal of Alternative and Complementary Medicine* 2016, **22**(6):A68.

86. Sotos-Prieto M, Bhupathiraju SN, Mattei J, Fung TT, Li Y, Pan A, Willett WC, Rimm EB, Hu FB: **Changes in diet quality scores and risk of cardiovascular disease among us men and women**. *Circulation* 2015, **131**.

87. O'Day K, Campbell DJ: **Cost-utility analysis of chocolate consumption for prevention of cardiometabolic disease**. *Value in Health* 2015, **18**(7):A395.

88. Liyanage T, Ninomiya T, Wang A, Jun M, Neal B, Wong MG, Jardine M, Hillis G, Perkovic V: **Effects of the mediterranean diet on cardiovascular outcomes: A systematic review and meta-analysis**. *Journal of the American College of Cardiology* 2015, **65**(10):A1448.

89. Krittanawong C, Tunhasiriwet A, Tweet MS, Bisanz KJ, Chirapongsathorn S, Hoy MB, Wang Z, Hayes SN: **Egg consumption and risk of cardiovascular disease: A systematic review and meta-analysis**. *Circulation* 2015, **132**.

90. Colpani V, Baena C, Jaspers L, Farajzadegan Z, Dhana K, Veloso G, Tielemans M, Kavousi M, Chowdhury R, Franco O: **Modifiable risk factors for prevention of cardiovascular disease and mortality in middle-aged women: Systematic review and meta-analysis**. *Circulation* 2015, **131**.

91. Strazzullo P, D'Elia L, Cairella G, Garbagnati F, Rossi G, Scalfi L: **Habitual coffee intake, blood pressure and risk of stroke: A meta-analysis of prospective studies**. *Circulation* 2014, **129**.

92. O'Day K, Campbell CM, Popelar BV: **Cost-utility analysis of coffee consumption for prevention of chronic disease and cancer in the United States**. *Value in Health* 2014, **17**(3):A91.

93. Khatibzadeh S, Afshin A, Micha R, Shi P, Yakoob MY, Singh GM, Rao M, Fahimi S, Ezzati M, Mozaffarian D: **The impact of low consumption of fruits and vegetables on mortality from cardiovascular diseases in 187 countries**. *Circulation* 2014, **129**.

94. Ekmekcioglu C: **The role of nutrition in health and behavior**. *Cell Membranes and Free Radical Research* 2014, **6**(1):317.

95. Camil CB: **Does calcium supplementation cause CVD?** *Climacteric* 2014, **17**:9.

96. Yakoob MY, Shi P, Hu FB, Campos H, Rexrode K, Mozaffarian D: **Circulating plasma dairy fatty acid biomarkers and incident stroke in two us prospective cohort studies**. *Circulation* 2013, **127**(12).

97. Strazzullo P, D'Elia L, Cappuccio FP: **Sodium and potassium intake and risk of cardiovascular events: Updated meta-analyses**. *Circulation* 2013, **127**(12).

98. Mozaffarian D, Fahimi S, Singh G, Micha R, Khatibzadeh S, Danaei G, Ezzati M, Lim S, Powles J: **The global impact of sodium consumption on cardiovascular mortality: A global, regional, and national comparative risk assessment**. *Circulation* 2013, **127**(12).

99. Liu P, Liu G, Dosieah S, Yuan G, Wang J: **Alcohol consumption and risk of stroke and coronary heart disease in Eastern Asian men: A meta-analysis of prospective cohort studies**. *Cardiology (Switzerland)* 2013, **126**:6.

100. Huang C, Tian Y, Huang J: **Sugar sweetened beverages consumption and risk of coronary heart disease and stroke: A meta-analysis of prospective studies**. *Cardiology (Switzerland)* 2013, **126**:19.

101. Franco Duran OH, Chowdhury RC, Stevens S, Warnakula S, Gorman D, Chowdhury S, Pan A, Ward H, Crowe F, Hu F: **Fish consumption, long-chain omega-3 fatty acids and cerebrovascular risk: A systematic review and meta-analysis**. *European Journal of Preventive Cardiology* 2013, **20**(1):S16.

102. Foroughi M, Akhavan M: **Stroke and nutrition: A review of studies**. *Annals of Nutrition and Metabolism* 2013, **63**:1136.

103. Afshin A, Micha R, Khatibzadeh S, Mozaffarian D: **Consumption of nuts and beans and risk of incident coronary heart disease, stroke, and diabetes mellitus: A systematic review and meta-analysis**. *Circulation* 2013, **127**(12).

104. Threapleton DE, Greenwood DC, Evans CE, Cleghorn CL, Nykjaer C, Woodhead C, Cade JE, Gale C, Burley VJ: **Dietary fibre intake and risk of stroke: A systematic review and meta-analysis of prospective studies**. *Proceedings of the Nutrition Society* 2012, **71**(OCE3):E248.

105. Strazzullo P, D'Elia L, Cappuccio FP: **Sodium and potassium intake and risk of cardiovascular accidents: Updated meta-analyses**. *High Blood Pressure and Cardiovascular Prevention* 2012, **19**(3):154.

106. Shin JY, Xun P, He K: **Egg consumption and risk of cardiovascular disease: Meta-analysis**. *Diabetes* 2012, **61**:A379-A380.

107. Pan A, Chen M, Chowdhury R, Wu J, Sun Q, Mozaffarian D, Hu FB: **Alpha-linolenic acid intake and biomarker in relation to risk of cardiovascular disease: A meta-analysis and systematic review**. *Circulation* 2012, **125**(10).

108. Paik J, Curhan G, Rexrode K, Manson J, Eric R, Taylor E: **A prospective study of calcium supplement intake and risk of cardiovascular disease in women**. *Journal of Bone and Mineral Research* 2012, **27**.

109. Obersby D, Chappell DC, Tsiami AA: **Are vegetarians susceptible to primary cardiovascular disease, due to a lack or absence of dietary animal produce?** *Proceedings of the Nutrition Society* 2012, **71**(OCE3):E241.

110. Gomes F, Hookway C, Emery PW, Weekes CE: **A systematic review of the evidence for oral nutritional supplements in patients at risk of malnutrition who have had a stroke**. *Cerebrovascular Diseases* 2012, **33**:15.

111. Francis RM: **Calcium supplements and cardiovascular disease: What level is safe?** *Maturitas* 2012, **71**:S3.

112. Fan J, Wang Y, Hui R, Zhang W: **Associations of dietary glycemic index and glycemic load with the risk of coronary heart disease, stroke, and stroke mortality: A meta-analysis of prospective studies**. *Cardiology (Switzerland)* 2012, **123**.

113. D'Elia L, Cairella G, Scalfi L, Garbagnati F, Rossi G, Strazzullo P: **Coffee intake and risk of hypertension and stroke: Meta-analysis of prospective studies**. *High Blood Pressure and Cardiovascular Prevention* 2012, **19**(3):153.

114. Sofi F, Abbate R, Gensini GF, Casini A: **The mediterranean diet in 2011**. *European Journal of Nutrition* 2011, **50**(6):497-498.

115. Rizos EC, Ntzani EE, Bika E, Kostapanos M, Elisaf MC: **Is the omega-3 fatty acids supplementation justified to modify major cardiovascular outcomes? A meta-analysis**. *Atherosclerosis Supplements* 2011, **12**(1):139-140.

116. Liu PM, Gang L, Shailendrasing D, Yuan GY, Wang JF: **Alcohol consumption and risk of stroke and coronary heart disease in eastern Asian men: A meta-analysis of prospective cohort studies**. *Heart* 2011, **97**:A91.

117. Franco Duran OH, Buitrago Lopez A, Sanderson J, Johnson L, Di Angelantonio E, Warnakula S, Wood A, Danesh J: **Chocolate consumption and cardiometabolic disorders: Systematic review and meta-analysis**. *European Heart Journal* 2011, **32**:717.

118. Almeida JC, Sarmento RA, Silva FM, Sbruzzi G, Schaan BD: **Micronutrient antioxidants and cardiovascular risk in patients with diabetes mellitus: A systematic review of observational studies**. *Endocrine Reviews* 2011, **32**(3).

119. Strazzullo P, D'Elia L, Barba G, Cappuccio FP: **Potassium intake and stroke risk: Meta-analysis of prospective studies**. *Journal of Hypertension* 2010, **28**:e239.

120. D'Elia L, Barba G, Cappuccio FP, Strazzull P: **Potassium intake and cardiovascular risk: A meta-analysis of prospective studies**. *High Blood Pressure and Cardiovascular Prevention* 2010, **17**(3):141.

121. Bolland M, Avenell A, Baron JA, Grey A, MacLennan GS, Gamble GD, Reid IR: **Effect of calcium supplements on the risk of myocardial infarction and cardiovascular events: A meta-analysis**. *Osteoporosis International* 2010, **21**:S21.

122. Clarke R: **Folic acid, homocysteine and risk of coronary heart disease, stroke and cancer: Meta-analysis of 8 trials involving 37,500 participants**. *Atherosclerosis Supplements* 2009, **10**(2).

123. Clarke R: **Folic acid, homocysteine and risk of coronary heart disease and stroke: Meta-analysis of 8 trials involving 37,500 participants**. *European Heart Journal* 2009, **30**:589.

124. Arab L, Liu W, Elashoff D: **Both black and green tea consumption associated with reduced risk of stroke**. *The FASEB Journal* 2009, **23**(S1).

125. Arab L, Elashoff D, Liu W: **Black and green tea consumption associated with reduced risk of ischemic stroke worldwide**. *Stroke* 2009, **40**(4):e128-e129.

126. Zhao B, Zeng L, Zhao J: **Erratum: Association of magnesium intake with type 2 diabetes and total stroke: An updated systematic review and meta-analysis (BMJ Open (2020) 10 (e032240) DOI: 10.1136/bmjopen-2019-032240)**. *BMJ Open* 2020, **10**(4).

127. **Erratum: Association between intakes of magnesium, potassium, and calcium and risk of stroke: 2 cohorts of US women and updated meta-analyses (American Journal of Clinical Nutrition (2015) 101 (1269-1277))**. *American Journal of Clinical Nutrition* 2015, **102**(4):981-982.

128. Micha R, Wallace SK, Mozaffarian D: **Response to letter regarding article, red and processed meat consumption and risk of incident coronary heart disease, stroke, and diabetes mellitus: A systematic review and meta-analysis**. *Circulation* 2011, **123**(3):e17.

129. **Erratum: Alcohol Consumption and Risk of Stroke: A Meta-analysis (Journal of the American Medical Association (February 5, 2003) 289 (579-588))**. *Journal of the American Medical Association* 2003, **289**(21):2798.

130. Bahonar A, Saadatnia M, Khorvash F, Maracy M, Khosravi A: **Carotenoids as potential antioxidant agents in stroke prevention: A systematic review**. *International Journal of Preventive Medicine* 2017, **8**:6-12.

131. Larsson SC: **Dietary Fiber Intake and Risk of Stroke**. *Current Nutrition Reports* 2014, **3**(2):88-93.

132. Kühn T: **Fish Consumption and the Risk of Stroke**. *Current Nutrition Reports* 2014, **3**(2):94-101.

133. Naude C: **Would an increase in vegetable and fruit intake help to reduce the burden of nutrition-related disease in South Africa? An umbrella review of the evidence**. *South African Journal of Clinical Nutrition* 2013, **26**(3):104-114.

134. Foroughi M, Akhavanzanjani M, Maghsoudi Z, Ghiasvand R, Khorvash F, Askari G: **Stroke and nutrition: A review of studies**. *International Journal of Preventive Medicine* 2013, **4**:S165-S179.

135. Sofi F, Abbate R, Gensini GF, Casini A: **Which diet for an effective cardiovascular prevention?** *Monaldi Archives for Chest Disease - Cardiac Series* 2012, **78**(2):60-65.

136. Liang W, Lee AH, Binns CW, Hu D, Huang R, Tian H: **Are soy foods protective against ischemic stroke?** *Future Neurology* 2007, **2**(5):505-511.

137. Ding EL, Hutfless SM, Ding X, Girotra S: **Chocolate and prevention of cardiovascular disease: A systematic review**. *Nutrition and Metabolism* 2006, **3**.

138. Zhang X, Chen X, Xu Y, Yang J, Du L, Li K, Zhou Y: **Milk consumption and multiple health outcomes: umbrella review of systematic reviews and meta-analyses in humans**. *Nutrition and Metabolism* 2021, **18**(1).

139. Li N, Wu X, Zhuang W, Xia L, Chen Y, Wu C, Rao Z, Du L, Zhao R, Yi M *et al*: **Tomato and lycopene and multiple health outcomes: Umbrella review**. *Food chemistry* 2021, **343**:128396.

140. Veronese N, Demurtas J, Pesolillo G, Celotto S, Barnini T, Calusi G, Caruso MG, Notarnicola M, Reddavide R, Stubbs B *et al*: **Magnesium and health outcomes: an umbrella review of systematic reviews and meta-analyses of observational and intervention studies**. *European journal of nutrition* 2020, **59**(1):263-272.

141. Marventano S, Godos J, Tieri M, Ghelfi F, Titta L, Lafranconi A, Gambera A, Alonzo E, Sciacca S, Buscemi S *et al*: **Egg consumption and human health: an umbrella review of observational studies**. *International journal of food sciences and nutrition* 2020, **71**(3):325-331.

142. Mah E, Chen CO, Liska DJ: **The effect of egg consumption on cardiometabolic health outcomes: an umbrella review**. *Public health nutrition* 2020, **23**(5):935-955.

143. Jayedi A, Shab-Bidar S: **Fish Consumption and the Risk of Chronic Disease: An Umbrella Review of Meta-Analyses of Prospective Cohort Studies**. *Advances in nutrition (Bethesda, Md)* 2020, **11**(5):1123-1133.

144. Godos J, Tieri M, Ghelfi F, Titta L, Marventano S, Lafranconi A, Gambera A, Alonzo E, Sciacca S, Buscemi S *et al*: **Dairy foods and health: an umbrella review of observational studies**. *International journal of food sciences and nutrition* 2020, **71**(2):138-151.

145. Yi M, Wu X, Zhuang W, Xia L, Chen Y, Zhao R, Wan Q, Du L, Zhou Y: **Tea Consumption and Health Outcomes: Umbrella Review of Meta-Analyses of Observational Studies in Humans**. *Molecular nutrition & food research* 2019, **63**(16):e1900389.

146. Viguiliouk E, Glenn AJ, Nishi SK, Chiavaroli L, Seider M, Khan T, Bonaccio M, Iacoviello L, Mejia SB, Jenkins DJA *et al*: **Associations between Dietary Pulses Alone or with Other Legumes and Cardiometabolic Disease Outcomes: An Umbrella Review and Updated Systematic Review and Meta-analysis of Prospective Cohort Studies**. *Advances in nutrition (Bethesda, Md)* 2019, **10**(Suppl_4):S308-s319.

147. Veronese N, Demurtas J, Celotto S, Caruso MG, Maggi S, Bolzetta F, Firth J, Smith L, Schofield P, Koyanagi A *et al*: **Is chocolate consumption associated with health outcomes? An umbrella review of systematic reviews and meta-analyses**. *Clinical nutrition (Edinburgh, Scotland)* 2019, **38**(3):1101-1108.

148. Khan SU, Khan MU, Riaz H, Valavoor S, Zhao D, Vaughan L, Okunrintemi V, Riaz IB, Khan MS, Kaluski E *et al*: **Effects of Nutritional Supplements and Dietary Interventions on Cardiovascular Outcomes: An Umbrella Review and Evidence Map**. *Annals of internal medicine* 2019, **171**(3):190-198.

149. Fontecha J, Calvo MV, Juarez M, Gil A, Martínez-Vizcaino V: **Milk and Dairy Product Consumption and Cardiovascular Diseases: An Overview of Systematic Reviews and Meta-Analyses**. *Advances in nutrition (Bethesda, Md)* 2019, **10**(suppl_2):S164-s189.

150. Altobelli E, Angeletti PM, Rapacchietta L, Petrocelli R: **Overview of Meta-Analyses: The Impact of Dietary Lifestyle on Stroke Risk**. *International journal of environmental research and public health* 2019, **16**(19).

151. Deng C, Lu Q, Gong B, Li L, Chang L, Fu L, Zhao Y: **Stroke and food groups: an overview of systematic reviews and meta-analyses**. *Public health nutrition* 2018, **21**(4):766-776.

152. Schwingshackl L, Hoffmann G, Missbach B, Stelmach-Mardas M, Boeing H: **An Umbrella Review of Nuts Intake and Risk of Cardiovascular Disease**. *Current pharmaceutical design* 2017, **23**(7):1016-1027.

153. Liu YS, Wu QJ, Lv JL, Jiang YT, Sun H, Xia Y, Chang Q, Zhao YH: **Dietary Carbohydrate and Diverse Health Outcomes: Umbrella Review of 30 Systematic Reviews and Meta-Analyses of 281 Observational Studies**. *Frontiers in nutrition* 2021, **8**:670411.

154. Li N, Wu X, Zhuang W, Xia L, Chen Y, Wang Y, Wu C, Rao Z, Du L, Zhao R *et al*: **Green leafy vegetable and lutein intake and multiple health outcomes**. *Food chemistry* 2021, **360**:130145.

155. Keller A, Wallace TC: **Tea intake and cardiovascular disease: an umbrella review**. *Annals of medicine* 2021, **53**(1):929-944.

156. McRae MP: **Dietary Fiber Is Beneficial for the Prevention of Cardiovascular Disease: An Umbrella Review of Meta-analyses**. *Journal of chiropractic medicine* 2017, **16**(4):289-299.

157. Li N, Wu X, Zhuang W, Xia L, Chen Y, Wu C, Rao Z, Du L, Zhao R, Yi M *et al*: **Tomato and lycopene and multiple health outcomes: Umbrella review**. *Food Chemistry* 2021, **343**.

158. Pana TA, Dehghani M, Baradaran HR, Neal SR, Wood AD, Kwok CS, Loke YK, Luben RN, Mamas MA, Khaw KT *et al*: **Calcium intake, calcium supplementation and cardiovascular disease and mortality in the British population: EPIC-norfolk prospective cohort study and meta-analysis**. *European journal of epidemiology* 2020.

159. Pagliai G, Dinu M, Madarena MP, Bonaccio M, Iacoviello L, Sofi F: **Consumption of ultra-processed foods and health status: a systematic review and meta-analysis**. *The British journal of nutrition* 2020:1-11.

160. Jayedi A, Soltani S, Abdolshahi A, Shab-Bidar S: **Fish consumption and the risk of cardiovascular disease and mortality in patients with type 2 diabetes: a dose-response meta-analysis of prospective cohort studies**. *Critical reviews in food science and nutrition* 2020:1-11.

161. Companys J, Pla-Pagà L, Calderón-Pérez L, Llauradó E, Solà R, Pedret A, Valls RM: **Fermented Dairy Products, Probiotic Supplementation, and Cardiometabolic Diseases: A Systematic Review and Meta-analysis**. *Advances in nutrition (Bethesda, Md)* 2020, **11**(4):834-863.

162. Yip CSC, Chan W, Fielding R: **The Associations of Fruit and Vegetable Intakes with Burden of Diseases: A Systematic Review of Meta-Analyses**. *Journal of the Academy of Nutrition and Dietetics* 2019, **119**(3):464-481.

163. Mazidi M, Mikhailidis DP, Sattar N, Howard G, Graham I, Banach M: **Consumption of dairy product and its association with total and cause specific mortality - A population-based cohort study and meta-analysis**. *Clinical nutrition (Edinburgh, Scotland)* 2019, **38**(6):2833-2845.

164. Barbaresko J, Neuenschwander M, Schwingshackl L, Schlesinger S: **Dietary factors and diabetes-related health outcomes in patients with type 2 diabetes: protocol for a systematic review and meta-analysis of prospective observational studies**. *BMJ open* 2019, **9**(7):e027298.

165. Kelly SA, Hartley L, Loveman E, Colquitt JL, Jones HM, Al-Khudairy L, Clar C, Germanò R, Lunn HR, Frost G *et al*: **Whole grain cereals for the primary or secondary prevention of cardiovascular disease**. *The Cochrane database of systematic reviews* 2017, **8**(8):Cd005051.

166. Dinu M, Abbate R, Gensini GF, Casini A, Sofi F: **Vegetarian, vegan diets and multiple health outcomes: A systematic review with meta-analysis of observational studies**. *Critical reviews in food science and nutrition* 2017, **57**(17):3640-3649.

167. Grosso G, Yang J, Marventano S, Micek A, Galvano F, Kales SN: **Nut consumption on all-cause, cardiovascular, and cancer mortality risk: a systematic review and meta-analysis of epidemiologic studies**. *The American journal of clinical nutrition* 2015, **101**(4):783-793.

168. Tran NL, Barraj LM, Heilman JM, Scrafford CG: **Egg consumption and cardiovascular disease among diabetic individuals: a systematic review of the literature**. *Diabetes, metabolic syndrome and obesity : targets and therapy* 2014, **7**:121-137.

169. Schwab U, Lauritzen L, Tholstrup T, Haldorssoni T, Riserus U, Uusitupa M, Becker W: **Effect of the amount and type of dietary fat on cardiometabolic risk factors and risk of developing type 2 diabetes, cardiovascular diseases, and cancer: a systematic review**. *Food & nutrition research* 2014, **58**.

170. Ding M, Bhupathiraju SN, Satija A, Van Dam RM, Hu FB: **Long-term coffee consumption and risk of cardiovascular disease: A systematic review and a dose-response meta-analysis of prospective cohort studies**. *Circulation* 2014, **129**(6):643-659.

171. Sarmento RA, Silva FM, Sbruzzi G, Schaan BD, Almeida JC: **Antioxidant micronutrients and cardiovascular risk in patients with diabetes: a systematic review**. *Arquivos brasileiros de cardiologia* 2013, **101**(3):240-248.

172. Arab L, Khan F, Lam H: **Tea consumption and cardiovascular disease risk**. *The American journal of clinical nutrition* 2013, **98**(6 Suppl):1651s-1659s.

173. Mellen PB, Walsh TF, Herrington DM: **Whole grain intake and cardiovascular disease: a meta-analysis**. *Nutrition, metabolism, and cardiovascular diseases : NMCD* 2008, **18**(4):283-290.

174. Zhang K, Chen X, Zhang L, Deng Z: **Fermented dairy foods intake and risk of cardiovascular diseases: A meta-analysis of cohort studies**. *Critical reviews in food science and nutrition* 2020, **60**(7):1189-1194.

175. Han M, Guan L, Ren Y, Zhao Y, Liu D, Zhang D, Liu L, Liu F, Chen X, Cheng C *et al*: **Dietary iron intake and risk of death due to cardiovascular diseases: A systematic review and dose-response meta-analysis of prospective cohort studies**. *Asia Pacific journal of clinical nutrition* 2020, **29**(2):309-321.

176. D'Elia L, Dinu M, Sofi F, Volpe M, Strazzullo P: **100% Fruit juice intake and cardiovascular risk: a systematic review and meta-analysis of prospective and randomised controlled studies**. *European journal of nutrition* 2020.

177. Micha R, Shulkin ML, Peñalvo JL, Khatibzadeh S, Singh GM, Rao M, Fahimi S, Powles J, Mozaffarian D: **Etiologic effects and optimal intakes of foods and nutrients for risk of cardiovascular diseases and diabetes: Systematic reviews and meta-analyses from the Nutrition and Chronic Diseases Expert Group (NutriCoDE)**. *PloS one* 2017, **12**(4):e0175149.

178. Chung M, Tang AM, Fu Z, Wang DD, Newberry SJ: **Calcium Intake and Cardiovascular Disease Risk: An Updated Systematic Review and Meta-analysis**. *Annals of internal medicine* 2016, **165**(12):856-866.

179. Alexander DD, Bylsma LC, Vargas AJ, Cohen SS, Doucette A, Mohamed M, Irvin SR, Miller PE, Watson H, Fryzek JP: **Dairy consumption and CVD: a systematic review and meta-analysis**. *The British journal of nutrition* 2016, **115**(4):737-750.

180. Malerba S, Turati F, Galeone C, Pelucchi C, Verga F, La Vecchia C, Tavani A: **A meta-analysis of prospective studies of coffee consumption and mortality for all causes, cancers and cardiovascular diseases**. *European journal of epidemiology* 2013, **28**(7):527-539.

181. Aburto NJ, Hanson S, Gutierrez H, Hooper L, Elliott P, Cappuccio FP: **Effect of increased potassium intake on cardiovascular risk factors and disease: systematic review and meta-analyses**. *BMJ (Clinical research ed)* 2013, **346**:f1378.

182. Peters U, Poole C, Arab L: **Does tea affect cardiovascular disease? A meta-analysis**. *American journal of epidemiology* 2001, **154**(6):495-503.

183. Thorning TK, Raben A, Tholstrup T, Soedamah-Muthu SS, Givens I, Astrup A: **Milk and dairy products: Good or bad for human health? An assessment of the totality of scientific evidence**. *Food and Nutrition Research* 2016, **60**.

184. McLaren L, Sumar N, Barberio AM, Trieu K, Lorenzetti DL, Tarasuk V, Webster J, Campbell NRC: **Population-level interventions in government jurisdictions for dietary sodium reduction**. *Cochrane Database of Systematic Reviews* 2016, **2016**(9).

185. Dallongeville J: **Omega-3 fatty acids and cardiovascular risk**. *Medecine des Maladies Metaboliques* 2009, **3**(5):491-495.

186. Danik JS, Manson JE: **Vitamin D and cardiovascular disease**. *Current Treatment Options in Cardiovascular Medicine* 2012, **14**(4):414-424.

187. Krittanawong C, Tunhasiriwet A, Zhang H, Prokop LJ, Chirapongsathorn S, Sun T, Wang Z: **Is white rice consumption a risk for metabolic and cardiovascular outcomes? a systematic review and meta-analysis**. *Heart Asia* 2019, **9**(2).

188. Hemmingsen B, Gimenez-Perez G, Mauricio D, Roqué IFM, Metzendorf MI, Richter B: **Diet, physical activity or both for prevention or delay of type 2 diabetes mellitus and its associated complications in people at increased risk of developing type 2 diabetes mellitus**. *The Cochrane database of systematic reviews* 2017, **12**(12):Cd003054.

189. Pasin G, Comerford KB: **Dairy foods and dairy proteins in the management of type 2 diabetes: a systematic review of the clinical evidence**. *Advances in nutrition (Bethesda, Md)* 2015, **6**(3):245-259.

190. Feskens EJ, Sluik D, van Woudenbergh GJ: **Meat consumption, diabetes, and its complications**. *Current diabetes reports* 2013, **13**(2):298-306.

191. Ribeiro EM, Alves M, Costa J, Ferreira JJ, Pinto FJ, Caldeira D: **Safety of coffee consumption after myocardial infarction: A systematic review and meta-analysis**. *Nutrition, metabolism, and cardiovascular diseases : NMCD* 2020, **30**(12):2146-2158.

192. Bonaccio M, Di Castelnuovo A, Costanzo S, Gialluisi A, Persichillo M, Cerletti C, Donati MB, de Gaetano G, Iacoviello L: **Mediterranean diet and mortality in the elderly: a prospective cohort study and a meta-analysis**. *The British journal of nutrition* 2018, **120**(8):841-854.

193. Ding M, Bhupathiraju SN, Satija A, van Dam RM, Hu FB: **Long-term coffee consumption and risk of cardiovascular disease: a systematic review and a dose-response meta-analysis of prospective cohort studies**. *Circulation* 2014, **129**(6):643-659.

194. Shahinfar H, Jayedi A, Khan TA, Shab-Bidar S: **Coffee consumption and cardiovascular diseases and mortality in patients with type 2 diabetes: A systematic review and dose-response meta-analysis of cohort studies**. *Nutrition, metabolism, and cardiovascular diseases : NMCD* 2021, **31**(9):2526-2538.

195. Cahoon D, Shertukde SP, Avendano EE, Tanprasertsuk J, Scott TM, Johnson EJ, Chung M, Nirmala N: **Walnut intake, cognitive outcomes and risk factors: a systematic review and meta-analysis**. *Annals of medicine* 2021, **53**(1):971-997.

196. Arnotti K, Bamber M: **Fruit and Vegetable Consumption and High-Density Lipoproteins in Overweight or Obese Individuals: A Meta-analysis**. *The Journal of cardiovascular nursing* 2021, **36**(1):78-87.

197. Hardy DS, Garvin JT, Xu H: **Carbohydrate quality, glycemic index, glycemic load and cardiometabolic risks in the US, Europe and Asia: A dose-response meta-analysis**. *Nutrition, metabolism, and cardiovascular diseases : NMCD* 2020, **30**(6):853-871.

198. Hayat K, Iqbal H, Malik U, Bilal U, Mushtaq S: **Tea and its consumption: benefits and risks**. *Critical reviews in food science and nutrition* 2015, **55**(7):939-954.

199. Mazzaglia G, Britton AR, Altmann DR, Chenet L: **Exploring the relationship between alcohol consumption and non-fatal or fatal stroke: A systematic review**. *Addiction* 2001, **96**(12):1743-1756.

200. Zhang W, Zhou F, Huang H, Mao Y, Ye D: **Biomarker of dietary linoleic acid and risk for stroke: A systematic review and meta-analysis**. *Nutrition (Burbank, Los Angeles County, Calif)* 2020, **79-80**:110953.

201. O’Connor EA, Evans CV, Rushkin MC, Redmond N, Lin JS: **U.S. Preventive Services Task Force Evidence Syntheses, formerly Systematic Evidence Reviews**. In: *Behavioral Counseling Interventions to Promote a Healthy Diet and Physical Activity for Cardiovascular Disease Prevention in Adults With Cardiovascular Risk Factors: Updated Systematic Review for the US Preventive Services Task Force.* edn. Rockville (MD): Agency for Healthcare Research and Quality (US); 2020.

202. Nyberg ST, Singh-Manoux A, Pentti J, Madsen IEH, Sabia S, Alfredsson L, Bjorner JB, Borritz M, Burr H, Goldberg M *et al*: **Association of Healthy Lifestyle With Years Lived Without Major Chronic Diseases**. *JAMA internal medicine* 2020, **180**(5):760-768.

203. Luo L, Meng H, Wang Z, Zhu S, Yuan S, Wang Y, Wang Q: **Effect of high-intensity exercise on cardiorespiratory fitness in stroke survivors: A systematic review and meta-analysis**. *Annals of physical and rehabilitation medicine* 2020, **63**(1):59-68.

204. Marklund M, Wu JHY, Imamura F, Del Gobbo LC, Fretts A, de Goede J, Shi P, Tintle N, Wennberg M, Aslibekyan S *et al*: **Biomarkers of Dietary Omega-6 Fatty Acids and Incident Cardiovascular Disease and Mortality**. *Circulation* 2019, **139**(21):2422-2436.

205. Sibbritt D, Peng W, Lauche R, Ferguson C, Frawley J, Adams J: **Efficacy of acupuncture for lifestyle risk factors for stroke: A systematic review**. *PloS one* 2018, **13**(10):e0206288.

206. Parappilly BP, Field TS, Mortenson WB, Sakakibara BM, Eng JJ: **Effectiveness of interventions involving nurses in secondary stroke prevention: A systematic review and meta-analysis**. *European journal of cardiovascular nursing : journal of the Working Group on Cardiovascular Nursing of the European Society of Cardiology* 2018, **17**(8):728-736.

207. Liang J, Zhou Q, Kwame Amakye W, Su Y, Zhang Z: **Biomarkers of dairy fat intake and risk of cardiovascular disease: A systematic review and meta analysis of prospective studies**. *Critical reviews in food science and nutrition* 2018, **58**(7):1122-1130.

208. Hackshaw A, Morris JK, Boniface S, Tang JL, Milenković D: **Low cigarette consumption and risk of coronary heart disease and stroke: meta-analysis of 141 cohort studies in 55 study reports**. *BMJ (Clinical research ed)* 2018, **360**:j5855.

209. Colpani V, Baena CP, Jaspers L, van Dijk GM, Farajzadegan Z, Dhana K, Tielemans MJ, Voortman T, Freak-Poli R, Veloso GGV *et al*: **Lifestyle factors, cardiovascular disease and all-cause mortality in middle-aged and elderly women: a systematic review and meta-analysis**. *European journal of epidemiology* 2018, **33**(9):831-845.

210. Barbaresko J, Rienks J, Nöthlings U: **Lifestyle Indices and Cardiovascular Disease Risk: A Meta-analysis**. *American journal of preventive medicine* 2018, **55**(4):555-564.

211. Yang B, Ren XL, Huang H, Guo XJ, Ma AG, Li D: **Circulating long-chain n-3 polyunsaturated fatty acid and incidence of stroke: a meta-analysis of prospective cohort studies**. *Oncotarget* 2017, **8**(48):83781-83791.

212. Zeng R, Xu CH, Xu YN, Wang YL, Wang M: **The effect of folate fortification on folic acid-based homocysteine-lowering intervention and stroke risk: a meta-analysis**. *Public health nutrition* 2015, **18**(8):1514-1521.

213. Li X, Xu J: **Dietary and circulating lycopene and stroke risk: a meta-analysis of prospective studies**. *Scientific reports* 2014, **4**:5031.

214. Chen X, Zhou L, Zhang Y, Yi D, Liu L, Rao W, Wu Y, Ma D, Liu X, Zhou XH *et al*: **Risk factors of stroke in Western and Asian countries: a systematic review and meta-analysis of prospective cohort studies**. *BMC public health* 2014, **14**:776.

215. Sun Q, Pan A, Hu FB, Manson JE, Rexrode KM: **25-hydroxyvitamin D levels and the risk of stroke: A prospective study and meta-analysis**. *Stroke* 2012, **43**(6):1470-1477.

216. Lawrence M, Kerr S, McVey C, Godwin J: **The effectiveness of secondary prevention lifestyle interventions designed to change lifestyle behavior following stroke: summary of a systematic review**. *International journal of stroke : official journal of the International Stroke Society* 2012, **7**(3):243-247.

217. Fan J, Song Y, Wang Y, Hui R, Zhang W: **Dietary glycemic index, glycemic load, and risk of coronary heart disease, stroke, and stroke mortality: a systematic review with meta-analysis**. *PloS one* 2012, **7**(12):e52182.

218. Lawrence M, Kerr S, McVey MC, Godwin J: **A systematic review of the effectiveness of secondary prevention lifestyle interventions designed to change lifestyle behaviour following stroke**. *JBI library of systematic reviews* 2011, **9**(43):1782-1827.

219. De Caterina R, Scarano M, Marfisi R, Lucisano G, Palma F, Tatasciore A, Marchioli R: **Cholesterol-lowering interventions and stroke: insights from a meta-analysis of randomized controlled trials**. *Journal of the American College of Cardiology* 2010, **55**(3):198-211.

220. Clarke R, Halsey J, Lewington S, Lonn E, Armitage J, Manson JE, Bønaa KH, Spence JD, Nygård O, Jamison R *et al*: **Effects of lowering homocysteine levels with B vitamins on cardiovascular disease, cancer, and cause-specific mortality: Meta-analysis of 8 randomized trials involving 37 485 individuals**. *Archives of internal medicine* 2010, **170**(18):1622-1631.

221. Clarke R, Armitage J, Lewington S, Collins R: **Homocysteine-lowering trials for prevention of vascular disease: protocol for a collaborative meta-analysis**. *Clinical chemistry and laboratory medicine* 2007, **45**(12):1575-1581.

222. Ariesen MJ, Claus SP, Rinkel GJ, Algra A: **Risk factors for intracerebral hemorrhage in the general population: a systematic review**. *Stroke* 2003, **34**(8):2060-2065.

223. Wald DS, Law M, Morris JK: **Homocysteine and cardiovascular disease: evidence on causality from a meta-analysis**. *BMJ (Clinical research ed)* 2002, **325**(7374):1202.

224. Boushey CJ, Beresford SA, Omenn GS, Motulsky AG: **A quantitative assessment of plasma homocysteine as a risk factor for vascular disease. Probable benefits of increasing folic acid intakes**. *Jama* 1995, **274**(13):1049-1057.

225. Soltani S, Arablou T, Jayedi A, Salehi-Abargouei A: **Adherence to the dietary approaches to stop hypertension (DASH) diet in relation to all-cause and cause-specific mortality: a systematic review and dose-response meta-analysis of prospective cohort studies**. *Nutrition journal* 2020, **19**(1):37.

226. Papadaki A, Nolen-Doerr E, Mantzoros CS: **The Effect of the Mediterranean Diet on Metabolic Health: A Systematic Review and Meta-Analysis of Controlled Trials in Adults**. *Nutrients* 2020, **12**(11).

227. Jalilpiran Y, Jayedi A, Djafarian K, Shab-Bidar S: **The Nordic diet and the risk of non-communicable chronic disease and mortality: a systematic review and dose-response meta-analysis of prospective cohort studies**. *Critical reviews in food science and nutrition* 2020:1-13.

228. Becerra-Tomás N, Blanco Mejía S, Viguiliouk E, Khan T, Kendall CWC, Kahleova H, Rahelić D, Sievenpiper JL, Salas-Salvadó J: **Mediterranean diet, cardiovascular disease and mortality in diabetes: A systematic review and meta-analysis of prospective cohort studies and randomized clinical trials**. *Critical reviews in food science and nutrition* 2020, **60**(7):1207-1227.

229. Saulle R, Lia L, De Giusti M, La Torre G: **A systematic overview of the scientific literature on the association between Mediterranean Diet and the Stroke prevention**. *La Clinica terapeutica* 2019, **170**(5):e396-e408.

230. Rosato V, Temple NJ, La Vecchia C, Castellan G, Tavani A, Guercio V: **Mediterranean diet and cardiovascular disease: a systematic review and meta-analysis of observational studies**. *European journal of nutrition* 2019, **58**(1):173-191.

231. Martínez-González MA, Gea A, Ruiz-Canela M: **The Mediterranean Diet and Cardiovascular Health**. *Circulation research* 2019, **124**(5):779-798.

232. Glenn AJ, Viguiliouk E, Seider M, Boucher BA, Khan TA, Blanco Mejia S, Jenkins DJA, Kahleová H, Rahelić D, Salas-Salvadó J *et al*: **Relation of Vegetarian Dietary Patterns With Major Cardiovascular Outcomes: A Systematic Review and Meta-Analysis of Prospective Cohort Studies**. *Frontiers in nutrition* 2019, **6**:80.

233. D'Alessandro A, Lampignano L, De Pergola G: **Mediterranean Diet Pyramid: A Proposal for Italian People. A Systematic Review of Prospective Studies to Derive Serving Sizes**. *Nutrients* 2019, **11**(6).

234. Chiavaroli L, Viguiliouk E, Nishi SK, Blanco Mejia S, Rahelić D, Kahleová H, Salas-Salvadó J, Kendall CW, Sievenpiper JL: **DASH Dietary Pattern and Cardiometabolic Outcomes: An Umbrella Review of Systematic Reviews and Meta-Analyses**. *Nutrients* 2019, **11**(2).

235. Chen GC, Neelakantan N, Martín-Calvo N, Koh WP, Yuan JM, Bonaccio M, Iacoviello L, Martínez-González MA, Qin LQ, van Dam RM: **Adherence to the Mediterranean diet and risk of stroke and stroke subtypes**. *European journal of epidemiology* 2019, **34**(4):337-349.

236. Salas-Salvadó J, Becerra-Tomás N, García-Gavilán JF, Bulló M, Barrubés L: **Mediterranean Diet and Cardiovascular Disease Prevention: What Do We Know?** *Progress in cardiovascular diseases* 2018, **61**(1):62-67.

237. Feng Q, Fan S, Wu Y, Zhou D, Zhao R, Liu M, Song Y: **Adherence to the dietary approaches to stop hypertension diet and risk of stroke: A meta-analysis of prospective studies**. *Medicine* 2018, **97**(38):e12450.

238. D'Alessandro A, De Pergola G: **The Mediterranean Diet: its definition and evaluation of a priori dietary indexes in primary cardiovascular prevention**. *International journal of food sciences and nutrition* 2018, **69**(6):647-659.

239. Grosso G, Marventano S, Yang J, Micek A, Pajak A, Scalfi L, Galvano F, Kales SN: **A comprehensive meta-analysis on evidence of Mediterranean diet and cardiovascular disease: Are individual components equal?** *Critical reviews in food science and nutrition* 2017, **57**(15):3218-3232.

240. Liyanage T, Ninomiya T, Wang A, Neal B, Jun M, Wong MG, Jardine M, Hillis GS, Perkovic V: **Effects of the Mediterranean Diet on Cardiovascular Outcomes-A Systematic Review and Meta-Analysis**. *PloS one* 2016, **11**(8):e0159252.

241. Zhang X, Shu L, Si C, Yu X, Gao W, Liao D, Zhang L, Liu X, Zheng P: **Dietary Patterns and Risk of Stroke in Adults: A Systematic Review and Meta-analysis of Prospective Cohort Studies**. *Journal of stroke and cerebrovascular diseases : the official journal of National Stroke Association* 2015, **24**(10):2173-2182.

242. Rodríguez-Monforte M, Flores-Mateo G, Sánchez E: **Dietary patterns and CVD: a systematic review and meta-analysis of observational studies**. *The British journal of nutrition* 2015, **114**(9):1341-1359.

243. Li F, Hou LN, Chen W, Chen PL, Lei CY, Wei Q, Tan WL, Zheng SB: **Associations of dietary patterns with the risk of all-cause, CVD and stroke mortality: a meta-analysis of prospective cohort studies**. *The British journal of nutrition* 2015, **113**(1):16-24.

244. Aljefree N, Ahmed F: **Association between dietary pattern and risk of cardiovascular disease among adults in the Middle East and North Africa region: a systematic review**. *Food & nutrition research* 2015, **59**:27486.

245. Kwok CS, Umar S, Myint PK, Mamas MA, Loke YK: **Vegetarian diet, Seventh Day Adventists and risk of cardiovascular mortality: a systematic review and meta-analysis**. *International journal of cardiology* 2014, **176**(3):680-686.

246. Kontogianni MD, Panagiotakos DB: **Dietary patterns and stroke: A systematic review and re-meta-analysis**. *Maturitas* 2014, **79**(1):41-47.

247. Salehi-Abargouei A, Maghsoudi Z, Shirani F, Azadbakht L: **Effects of Dietary Approaches to Stop Hypertension (DASH)-style diet on fatal or nonfatal cardiovascular diseases--incidence: a systematic review and meta-analysis on observational prospective studies**. *Nutrition (Burbank, Los Angeles County, Calif)* 2013, **29**(4):611-618.

248. Psaltopoulou T, Sergentanis TN, Panagiotakos DB, Sergentanis IN, Kosti R, Scarmeas N: **Mediterranean diet, stroke, cognitive impairment, and depression: A meta-analysis**. *Annals of neurology* 2013, **74**(4):580-591.

249. Sherzai A, Heim LT, Boothby C, Sherzai AD: **Stroke, food groups, and dietary patterns: a systematic review**. *Nutrition reviews* 2012, **70**(8):423-435.

250. Scarborough P, Nnoaham KE, Clarke D, Capewell S, Rayner M: **Modelling the impact of a healthy diet on cardiovascular disease and cancer mortality**. *Journal of epidemiology and community health* 2012, **66**(5):420-426.

251. Hoevenaar-Blom MP, Nooyens AC, Kromhout D, Spijkerman AM, Beulens JW, van der Schouw YT, Bueno-de-Mesquita B, Verschuren WM: **Mediterranean style diet and 12-year incidence of cardiovascular diseases: the EPIC-NL cohort study**. *PloS one* 2012, **7**(9):e45458.

252. Sofi F, Abbate R, Gensini GF, Casini A: **Accruing evidence on benefits of adherence to the Mediterranean diet on health: an updated systematic review and meta-analysis**. *The American journal of clinical nutrition* 2010, **92**(5):1189-1196.

253. Lloyd-Williams F, Mwatsama M, Ireland R, Capewell S: **Small changes in snacking behaviour: the potential impact on CVD mortality**. *Public health nutrition* 2009, **12**(6):871-876.

254. Ginter E: **Vegetarian diets, chronic diseases and longevity**. *Bratislavske lekarske listy* 2008, **109**(10):463-466.

255. Hajjar M, Rezazadeh A: **The recommended food score and healthy nordic food index in cardiovascular disease and stroke: A systematic review**. *ARYA Atherosclerosis* 2020, **16**(5):248-257.

256. Rodríguez-Monforte M, Flores-Mateo G, Sánchez E: **Dietary patterns and CVD: A systematic review and meta-analysis of observational studies**. *British Journal of Nutrition* 2015, **114**(9):1341-1359.

257. Ding C, O'Neill D, Bell S, Stamatakis E, Britton A: **Association of alcohol consumption with morbidity and mortality in patients with cardiovascular disease: original data and meta-analysis of 48,423 men and women**. *BMC medicine* 2021, **19**(1):167.

258. Lu JW, Yu LH, Tu YK, Cheng HY, Chen LY, Loh CH, Chen TL: **Risk of Incident Stroke among Vegetarians Compared to Nonvegetarians: A Systematic Review and Meta-Analysis of Prospective Cohort Studies**. *Nutrients* 2021, **13**(9).

259. Green M, Styles T, Russell T, Sada C, Jallow E, Stewart J, Lazariashvili O, Lubomirova I, Cotlarciuc I, Sharma S *et al*: **Non-genetic and genetic risk factors for adult cerebral venous thrombosis**. *Thrombosis research* 2018, **169**:15-22.

260. He FJ, MacGregor GA: **How Far Should Salt Intake Be Reduced?** *Hypertension* 2003, **42**(6):1093-1099.

261. Ding EL, Hutfless SM, Ding X, Girotra S: **Chocolate and prevention of cardiovascular disease: a systematic review**. *Nutrition & metabolism* 2006, **3**:2.

262. Wang C, Harris WS, Chung M, Lichtenstein AH, Balk EM, Kupelnick B, Jordan HS, Lau J: **n-3 Fatty acids from fish or fish-oil supplements, but not alpha-linolenic acid, benefit cardiovascular disease outcomes in primary- and secondary-prevention studies: a systematic review**. *The American journal of clinical nutrition* 2006, **84**(1):5-17.

263. Rees K, Guraewal S, Wong YL, Majanbu DL, Mavrodaris A, Stranges S, Kandala NB, Clarke A, Franco OH: **Is vitamin K consumption associated with cardio-metabolic disorders? A systematic review**. *Maturitas* 2010, **67**(2):121-128.

264. Drouin-Chartier JP, Brassard D, Tessier-Grenier M, Côté JA, Labonté M, Desroches S, Couture P, Lamarche B: **Systematic Review of the Association between Dairy Product Consumption and Risk of Cardiovascular-Related Clinical Outcomes**. *Advances in nutrition (Bethesda, Md)* 2016, **7**(6):1026-1040.

265. Iacoviello L, Bonaccio M, Cairella G, Catani MV, Costanzo S, D'Elia L, Giacco R, Rendina D, Sabino P, Savini I *et al*: **Diet and primary prevention of stroke: Systematic review and dietary recommendations by the ad hoc Working Group of the Italian Society of Human Nutrition**. *Nutrition, metabolism, and cardiovascular diseases : NMCD* 2018, **28**(4):309-334.

266. Ismail SR, Maarof SK, Siedar Ali S, Ali A: **Systematic review of palm oil consumption and the risk of cardiovascular disease**. *PloS one* 2018, **13**(2):e0193533.

267. Kim Y, Keogh J, Clifton PM: **Nuts and Cardio-Metabolic Disease: A Review of Meta-Analyses**. *Nutrients* 2018, **10**(12).

268. Kim Y, Keogh JB, Clifton PM: **Does Nut Consumption Reduce Mortality and/or Risk of Cardiometabolic Disease? An Updated Review Based on Meta-Analyses**. *International journal of environmental research and public health* 2019, **16**(24).

269. Chen X, Zhang Z, Yang H, Qiu P, Wang H, Wang F, Zhao Q, Fang J, Nie J: **Consumption of ultra-processed foods and health outcomes: a systematic review of epidemiological studies**. *Nutrition journal* 2020, **19**(1):86.

270. Evans CEL: **Dietary fibre and cardiovascular health: a review of current evidence and policy**. *The Proceedings of the Nutrition Society* 2020, **79**(1):61-67.

271. Gonçalves C, Abreu S: **Sodium and Potassium Intake and Cardiovascular Disease in Older People: A Systematic Review**. *Nutrients* 2020, **12**(11).

272. Krittanawong C, Isath A, Hahn J, Wang Z, Fogg SE, Bandyopadhyay D, Jneid H, Virani SS, Tang WHW: **Mushroom Consumption and Cardiovascular Health: A Systematic Review**. *The American journal of medicine* 2020.

273. So J, Avendano EE, Raman G, Johnson EJ: **Potato consumption and risk of cardio-metabolic diseases: evidence mapping of observational studies**. *Systematic reviews* 2020, **9**(1):274.

274. Manson JE, Bassuk SS, Cook NR, Lee IM, Mora S, Albert CM, Buring JE: **Vitamin D, Marine n-3 Fatty Acids, and Primary Prevention of Cardiovascular Disease Current Evidence**. *Circulation research* 2020, **126**(1):112-128.

275. Hooper L, Martin N, Jimoh OF, Kirk C, Foster E, Abdelhamid AS: **Reduction in saturated fat intake for cardiovascular disease**. *The Cochrane database of systematic reviews* 2020, **5**(5):Cd011737.

276. Abdelhamid AS, Brown TJ, Brainard JS, Biswas P, Thorpe GC, Moore HJ, Deane KH, Summerbell CD, Worthington HV, Song F *et al*: **Omega-3 fatty acids for the primary and secondary prevention of cardiovascular disease**. *The Cochrane database of systematic reviews* 2020, **3**(2):Cd003177.

277. Rui Q, Ni H, Liu H, Zhu X, Gao R: **Coffee and tea consumption and the risk for subarachnoid hemorrhage: A meta-analysis**. *Nutrition (Burbank, Los Angeles County, Calif)* 2019, **59**:21-28.

278. Reynolds A, Mann J, Cummings J, Winter N, Mete E, Te Morenga L: **Carbohydrate quality and human health: a series of systematic reviews and meta-analyses**. *Lancet (London, England)* 2019, **393**(10170):434-445.

279. Raman G, Avendano EE, Chen S, Wang J, Matson J, Gayer B, Novotny JA, Cassidy A: **Dietary intakes of flavan-3-ols and cardiometabolic health: systematic review and meta-analysis of randomized trials and prospective cohort studies**. *The American journal of clinical nutrition* 2019, **110**(5):1067-1078.

280. Jayedi A, Ghomashi F, Zargar MS, Shab-Bidar S: **Dietary sodium, sodium-to-potassium ratio, and risk of stroke: A systematic review and nonlinear dose-response meta-analysis**. *Clinical nutrition (Edinburgh, Scotland)* 2019, **38**(3):1092-1100.

281. Gayer BA, Avendano EE, Edelson E, Nirmala N, Johnson EJ, Raman G: **Effects of Intake of Apples, Pears, or Their Products on Cardiometabolic Risk Factors and Clinical Outcomes: A Systematic Review and Meta-Analysis**. *Current developments in nutrition* 2019, **3**(10):nzz109.

282. Kim J, Choi J, Kwon SY, McEvoy JW, Blaha MJ, Blumenthal RS, Guallar E, Zhao D, Michos ED: **Association of Multivitamin and Mineral Supplementation and Risk of Cardiovascular Disease: A Systematic Review and Meta-Analysis**. *Circulation Cardiovascular quality and outcomes* 2018, **11**(7):e004224.

283. Jenkins DJA, Spence JD, Giovannucci EL, Kim YI, Josse R, Vieth R, Blanco Mejia S, Viguiliouk E, Nishi S, Sahye-Pudaruth S *et al*: **Supplemental Vitamins and Minerals for CVD Prevention and Treatment**. *Journal of the American College of Cardiology* 2018, **71**(22):2570-2584.

284. Hooper L, Al-Khudairy L, Abdelhamid AS, Rees K, Brainard JS, Brown TJ, Ajabnoor SM, O'Brien AT, Winstanley LE, Donaldson DH *et al*: **Omega-6 fats for the primary and secondary prevention of cardiovascular disease**. *The Cochrane database of systematic reviews* 2018, **11**(11):Cd011094.

285. Abdelhamid AS, Martin N, Bridges C, Brainard JS, Wang X, Brown TJ, Hanson S, Jimoh OF, Ajabnoor SM, Deane KH *et al*: **Polyunsaturated fatty acids for the primary and secondary prevention of cardiovascular disease**. *The Cochrane database of systematic reviews* 2018, **7**(7):Cd012345.

286. Abdelhamid AS, Brown TJ, Brainard JS, Biswas P, Thorpe GC, Moore HJ, Deane KH, AlAbdulghafoor FK, Summerbell CD, Worthington HV *et al*: **Omega-3 fatty acids for the primary and secondary prevention of cardiovascular disease**. *The Cochrane database of systematic reviews* 2018, **11**(11):Cd003177.

287. Krittanawong C, Tunhasiriwet A, Zhang H, Prokop LJ, Chirapongsathorn S, Sun T, Wang Z: **Is white rice consumption a risk for metabolic and cardiovascular outcomes? A systematic review and meta-analysis**. *Heart Asia* 2017, **9**(2):e010909.

288. Pang J, Zhang Z, Zheng TZ, Bassig BA, Mao C, Liu X, Zhu Y, Shi K, Ge J, Yang YJ *et al*: **Green tea consumption and risk of cardiovascular and ischemic related diseases: A meta-analysis**. *International journal of cardiology* 2016, **202**:967-974.

289. Leermakers ET, Darweesh SK, Baena CP, Moreira EM, Melo van Lent D, Tielemans MJ, Muka T, Vitezova A, Chowdhury R, Bramer WM *et al*: **The effects of lutein on cardiometabolic health across the life course: a systematic review and meta-analysis**. *The American journal of clinical nutrition* 2016, **103**(2):481-494.

290. Hooper L, Summerbell CD, Thompson R, Sills D, Roberts FG, Moore HJ, Smith GD: **Reduced or modified dietary fat for preventing cardiovascular disease**. *Sao Paulo medical journal = Revista paulista de medicina* 2016, **134**(2):182-183.

291. Hooper L, Martin N, Abdelhamid A, Davey Smith G: **Reduction in saturated fat intake for cardiovascular disease**. *The Cochrane database of systematic reviews* 2015(6):Cd011737.

292. Hookway C, Gomes F, Weekes CE: **Royal College of Physicians Intercollegiate Stroke Working Party evidence-based guidelines for the secondary prevention of stroke through nutritional or dietary modification**. *Journal of human nutrition and dietetics : the official journal of the British Dietetic Association* 2015, **28**(2):107-125.

293. Martínez-González MA, Dominguez LJ, Delgado-Rodríguez M: **Olive oil consumption and risk of CHD and/or stroke: a meta-analysis of case-control, cohort and intervention studies**. *The British journal of nutrition* 2014, **112**(2):248-259.

294. Graudal N, Jürgens G, Baslund B, Alderman MH: **Compared with usual sodium intake, low- and excessive-sodium diets are associated with increased mortality: a meta-analysis**. *American journal of hypertension* 2014, **27**(9):1129-1137.

295. Kruse LG, Ogletree RL, Jr.: **Omega-3 fatty acids and cardiovascular risk**. *Journal of the Mississippi State Medical Association* 2013, **54**(6):156-157.

296. Droste DW, Keipes M: **[The reduction of stroke risk, risk of myocardial infarction and death by healthy diet and physical activity]**. *Bulletin de la Societe des sciences medicales du Grand-Duche de Luxembourg* 2013(2):51-62.

297. Kotwal S, Jun M, Sullivan D, Perkovic V, Neal B: **Omega 3 Fatty acids and cardiovascular outcomes: systematic review and meta-analysis**. *Circulation Cardiovascular quality and outcomes* 2012, **5**(6):808-818.

298. Hooper L, Summerbell CD, Thompson R, Sills D, Roberts FG, Moore HJ, Davey Smith G: **Reduced or modified dietary fat for preventing cardiovascular disease**. *The Cochrane database of systematic reviews* 2012, **2012**(5):Cd002137.

299. Gibson CL, Murphy AN, Murphy SP: **Stroke outcome in the ketogenic state--a systematic review of the animal data**. *Journal of neurochemistry* 2012, **123 Suppl 2**(0 2):52-57.

300. Hooper L, Summerbell CD, Thompson R, Sills D, Roberts FG, Moore H, Davey Smith G: **Reduced or modified dietary fat for preventing cardiovascular disease**. *The Cochrane database of systematic reviews* 2011(7):Cd002137.

301. Clarke R, Lewington S, Sherliker P, Armitage J: **Effects of B-vitamins on plasma homocysteine concentrations and on risk of cardiovascular disease and dementia**. *Current opinion in clinical nutrition and metabolic care* 2007, **10**(1):32-39.

302. Huang HY, Caballero B, Chang S, Alberg AJ, Semba RD, Schneyer CR, Wilson RF, Cheng TY, Vassy J, Prokopowicz G *et al*: **The efficacy and safety of multivitamin and mineral supplement use to prevent cancer and chronic disease in adults: a systematic review for a National Institutes of Health state-of-the-science conference**. *Annals of internal medicine* 2006, **145**(5):372-385.

303. Bazzano LA, Reynolds K, Holder KN, He J: **Effect of folic acid supplementation on risk of cardiovascular diseases: a meta-analysis of randomized controlled trials**. *Jama* 2006, **296**(22):2720-2726.

304. Yzebe D, Lievre M: **Fish oils in the care of coronary heart disease patients: a meta-analysis of randomized controlled trials**. *Fundamental & clinical pharmacology* 2004, **18**(5):581-592.

305. Walters MR, Williamson C, Lunn K, Munteanu A: **Chocolate consumption and risk of stroke: A prospective cohort of men and meta-analysis**. *Neurology* 2013, **80**(12):1173-1174.

306. Hooper L, Al-Khudairy L, Abdelhamid AS, Rees K, Brainard JS, Brown TJ, Ajabnoor SM, O'Brien AT, Winstanley LE, Donaldson DH *et al*: **Omega-6 fats for the primary and secondary prevention of cardiovascular disease**. *Cochrane Database of Systematic Reviews* 2018, **2018**(11).

307. Qian Y, Ye D, Huang H, Wu DJH, Zhuang Y, Jiang X, Mao Y: **Coffee Consumption and Risk of Stroke: A Mendelian Randomization Study**. *Annals of neurology* 2020, **87**(4):525-532.

308. Schwingshackl L, Knüppel S, Michels N, Schwedhelm C, Hoffmann G, Iqbal K, De Henauw S, Boeing H, Devleesschauwer B: **Intake of 12 food groups and disability-adjusted life years from coronary heart disease, stroke, type 2 diabetes, and colorectal cancer in 16 European countries**. *European journal of epidemiology* 2019, **34**(8):765-775.

309. Greenberg JA, Manson JE, Neuhouser ML, Tinker L, Eaton C, Johnson KC, Shikany JM: **Chocolate intake and heart disease and stroke in the Women's Health Initiative: a prospective analysis**. *The American journal of clinical nutrition* 2018, **108**(1):41-48.

310. Kiechl S, Traindl O: **[Prevention of stroke]**. *Wiener medizinische Wochenschrift (1946)* 2003, **153**(1-2):6-13.

311. D'Elia L, Barba G, Cappuccio FP, Strazzullo P: **Potassium intake, stroke and cardiovascular disease: A meta-analysis of prospective studies**. *Vasomed* 2011, **23**(3):141.

312. Dauchet L, Amouyel P, Dallongeville J: **Consumption of fruit and vegetables and cardiovascular risks: Meta-analysis of prospective epidemiological studies**. *Cahiers de Nutrition et de Dietetique* 2005, **40**(1):31-40.
