# Supplementary material for: Role of diet in stroke incidence: an umbrella review of meta-analyses of prospective observational studies
Source: BMC Med. 2022 May 24;20:194. doi: 10.1186/s12916-022-02381-6 (PMC9128224; doi:10.1186/s12916-022-02381-6)
Supplement: Supplementary file 2 — Additional file 2: Table S2. Characteristics of included meta-analyses evaluating associations between food groups, foods as well as beverages and stroke risk. [file 12916_2022_2381_MOESM2_ESM.docx]

|  | |  |  |  | | | | | | | | | | | | | | | | |
| --- | --- | --- | --- | --- | --- | --- | --- | --- | --- | --- | --- | --- | --- | --- | --- | --- | --- | --- | --- | --- |
|  | |  |  |  | | | | | | | | | | | | | | | | |
|  | |  |  |  | | | | | | | | | | | | | | | | |
| **Table S2.** **Characteristics of included meta-analyses evaluating** **associations between** **food groups, foods as well as beverages and stroke risk.** | | | | | | | | | | | | | | | | | | | | |
| **Dietary factor** | **Author, year** | | | | **Comparison** | **Article retrieval time** | **Duration of follow up (years)** | **Assessment tool of original study** | **Studies** | **Subjects** | **Cases** | **Relative risk and 95% CIs** | | **Random *P* value** | **Fixed *P* value** | **Heterogeneity** | | **Small-study**  **effects** | **95%PI** | **Includedin main analysis** |
|  |  |  |  |  |  |  |  |  | **（n）** | **（n）** | **（n）** | **Random effects** | **Fixed effects** |  |  | ***P-value*** | ***I²*** |  |  |  |
| total grains | | | | | | | | | | | | | | | | | | | | |
|  | Chen J, 2016 [14] | | | | high versus low intake | Feb 2016 | 5.5-24.0 | NOS | 8 | 407199 | 5264 | RR:0.97 (0.83-1.14) | 0.95 (0.85-1.06) | 0.738 | 0.345 | 0.099 | 42% | 0.513 | 0.65-1.46 | N |
|  | Aune D, 2016 [15] | | | | high versus low intake | Apr 2016 | 5.5-26.0 | NOS | 4 | 107858 | 4425 | RR:0.89 (0.79-0.995) | 0.89 (0.79-0.99) | 0.041 | 0.035 | 0.376 | 3% | 0.884 | 0.68-1.17 | N |
|  | Chen J, 2016 [14] | | | | per 3 servings/day | Feb 2016 | 5.5-24.0 | NOS | 6 | 327746 | 4287 | RR:0.97 (0.90-1.03) | 0.97 (0.94-1.01) | 0.311 | 0.136 | 0.023 | 62% | 0.830 | 0.80-1.18 | Y |
|  | Aune D, 2016 [15] | | | | per 90 g/day | Apr 2016 | 5.5-26.0 | NOS | 5 | 132055 | 5105 | RR:0.93 (0.85-1.02) | 0.95 (0.90-0.99) | 0.132 | 0.017 | 0.031 | 63% | 0.503 | 0.69-1.25 | N |
| whole grains | | | | | | | | | | | | | | | | | | | | |
|  | Chen J, 2016 [14] | | | | high versus low intake | Feb 2016 | 5.5-24.0 | NOS | 5 | 204895 | 1751 | RR:0.92 (0.72-1.17) | 0.93 (0.80-1.09) | 0.502 | 0.382 | 0.076 | 53% | 0.859 | 0.45-1.88 | N |
|  | Chen G, 2016 [16] | | | | high versus low intake | Jun 2015 | 5.5-26.0 | NOS | 4 | 136489 | 987 | RR:0.96 (0.75-1.22) | 0.94 (0.78-1.13) | 0.713 | 0.511 | 0.175 | 40% | 0.195 | 0.41-2.27 | N |
|  | Aune D, 2016 [15] | | | | high versus low intake | Apr 2016 | 5.5-26.0 | NOS | 5 | 242567 | 1885 | RR:0.87 (0.72-1.05) | 0.89 (0.77-1.03) | 0.141 | 0.115 | 0.208 | 32% | 0.156 | 0.54-1.42 | N |
|  | Chen J, 2016 [14] | | | | per 3 servings/day | Feb 2016 | 5.5-24.0 | NOS | 5 | 204895 | 1751 | RR:0.88 (0.64-1.22) | 0.85 (0.74-0.97) | 0.441 | 0.017 | 0.001 | 80% | 0.614 | 0.28-2.74 | N |
|  | Chen G, 2016 [16] | | | | per 50 g /day | Jun 2015 | 5.5-26.0 | NOS | 3 | 125512 | 840 | RR:0.93 (0.54-1.62) | 0.86 (0.65-1.13) | 0.805 | 0.271 | 0.078 | 61% | 0.603 | 0.00-343.84 | N |
|  | Aune D, 2016 [15] | | | | per 90 g/day | Apr 2016 | 5.5-26.0 | NOS | 6 | 245012 | 2337 | RR:0.87 (0.75-1.02) | 0.95 (0.87-1.04) | 0.093 | 0.259 | 0.047 | 56% | 0.010 | 0.56-1.35 | N |
|  | Li B, 2016 [17] | | | | per 1 serving/day | Nov 2015 | NA | NOS | 4 | 240532 | 840 | RR:0.96 (0.91-1.00) | 0.95 (0.92-0.99) | 0.074 | 0.005 | 0.161 | 42% | 0.204 | 0.80-1.15 | N |
|  | Fang L, 2016 [18] | | | | high versus low intake | May 2015 | 5.5-24.0 | NA | 6 | 247487 | 1635 | RR:0.86 (0.73-1.01) | 0.86 (0.73-1.01) | 0.071 | 0.071 | 0.551 | 0% | 0.836 | 0.68-1.08 | N |
|  | Bechthold A, 2019 [19] | | | | high versus low intake | Mar 2017 | 10.4-24.0 | NutriGrade | 7 | 417505 | 11116 | RR:0.92 (0.82-1.02) | 0.90 (0.85-0.96) | 0.107 | 0.001 | 0.046 | 53% | 0.717 | 0.69-1.23 | N |
|  | Bechthold A, 2019 [19] | | | | per 30 g/day | Mar 2017 | 10.4-24.0 | NutriGrade | 4 | 302801 | 5863 | RR:0.99 (0.95-1.03) | 1.00 (0.99-1.01) | 0.623 | 0.912 | 0.033 | 66% | 0.491 | 0.84-1.16 | Y |
| refined grains | | | | | | | | | | | | | | | | | | | | |
|  | Chen J, 2016 [14] | | | | high versus low intake | Feb 2016 | 5.5-24.0 | NOS | 5 | 204895 | 1751 | RR:0.99 (0.84-1.16) | 0.99 (0.84-1.16) | 0.866 | 0.866 | 0.435 | 0% | 0.489 | 0.76-1.29 | N |
|  | Aune D, 2016 [15] | | | | high versus low intake | Apr 2016 | 5.5-26.0 | NOS | 4 | 122557 | 1605 | RR:0.95 (0.78-1.15) | 0.93 (0.79-1.08) | 0.568 | 0.321 | 0.27 | 24% | 0.535 | 0.52-1.73 | N |
|  | Wu D, 2015 [20] | | | | high versus low intake | Nov 2014 | 5.5-15.2 | NOS | 10 | 410821 | 8284 | RR:1.02 (0.93-1.10) | 1.02 (0.93-1.10) | 0.719 | 0.719 | 0.97 | 0% | 0.563 | 0.92-1.13 | N |
|  | Chen J, 2016 [14] | | | | per 3 servings/day | Feb 2016 | 5.5-24.0 | NOS | 5 | 204895 | 1751 | RR:0.95 (0.86-1.04) | 0.95 (0.86-1.04) | 0.216 | 0.216 | 0.504 | 0% | 0.337 | 0.81-1.11 | N |
|  | Aune D, 2016 [15] | | | | per 90 g/day | Apr 2016 | 5.5-26.0 | NOS | 5 | 125002 | 2075 | RR:0.91 (0.81-1.02) | 0.93 (0.87-0.99) | 0.097 | 0.024 | 0.245 | 27% | 0.588 | 0.68-1.22 | N |
|  | Wu D, 2015 [20] | | | | per 3 servings/day | Nov 2014 | 5.5-15.2 | NOS | 10 | 410821 | 8284 | RR:0.98 (0.93-1.03) | 0.98 (0.94-1.03) | 0.423 | 0.373 | 0.349 | 10% | 0.565 | 0.90-1.07 | Y |
|  | Bechthold A, 2019 [19] | | | | high versus low intake | Mar 2017 | 11.0-24.0 | NutriGrade | 6 | 420469 | 11434 | RR:1.02 (0.94-1.10) | 1.08 (0.94-1.10) | 0.678 | 0.673 | 0.406 | 2% | 0.703 | 0.91-1.15 | N |
|  | Bechthold A, 2019 [19] | | | | per 30 g/day | Mar 2017 | 11.0-24.0 | NutriGrade | 4 | 318503 | 9906 | RR:1.00 (0.99-1.01) | 1.00 (0.99-1.01) | 0.486 | 0.486 | 0.474 | 0% | 0.203 | 0.98-1.02 | N |
| whole grain bread | | | | | | | | | | | | | | | | | | | | |
|  | Aune D, 2016 [15] | | | | high versus low intake | Apr 2016 | 5.5-26.0 | NOS | 2 | 130751 | 636 | RR:0.88 (0.75-1.03) | 0.88 (0.75-1.03) | 0.121 | 0.121 | 0.891 | 0% | NA | NA | Y |
| whole grain breakfast cereals | | | | | | | | | | | | | | | | | | | | |
|  | Aune D, 2016 [15] | | | | high versus low intake | Apr 2016 | 5.5-26.0 | NOS | 2 | 206200 | 426 | RR:0.99 (0.53-1.86) | 0.89 (0.68-1.17) | 0.976 | 0.409 | 0.035 | 77% | NA | NA | N |
|  | Aune D, 2016 [15] | | | | per 90 g/day | Apr 2016 | 5.5-26.0 | NOS | 2 | 206200 | 426 | RR:1.07 (0.69-1.64） | 0.96 (0.82-1.12) | 0.773 | 0.576 | 0.032 | 78% | NA | NA | Y |
| rice | | | | | | | | | | | | | | | | | | | | |
|  | Aune D, 2016 [15] | | | | high versus low intake | Apr 2016 | 5.5-26.0 | NOS | 4 | 410393 | 10954 | RR:1.02 (0.94-1.11) | 1.02 (0.94-1.11) | 0.648 | 0.648 | 0.991 | 0% | 0.661 | 0.85-1.22 | N |
|  | Aune D, 2016 [15] | | | | per 100 g/day | Apr 2016 | 5.5-26.0 | NOS | 4 | 410393 | 10954 | RR:0.99 (0.96-1.02) | 0.99 (0.96-1.02) | 0.618 | 0.618 | 0.830 | 0% | 0.894 | 0.93-1.06 | Y |
| oat | | | | | | | | | | | | | | | | | | | | |
|  | Wehrli F, 2021 [21] | | | | high versus low intake | Sep 2020 | 6.0-26.0 | GRADE | 4 | 312172 | NA | RR:0.79 (0.59-1.07） | 0.94 (0.92-0.95) | 0.131 | <0.001 | 0.000 | 97% | 0.401 | 0.19-3.23 | Y |
| fruits and vegetables | | | | | | | | | | | | | | | | | | | | |
|  | Aune D, 2017 [22] | | | | high versus low intake | Sep 2016 | NA | NA | 8 | 226910 | 10560 | RR:0.79 (0.71-0.88) | 0.79 (0.74-0.85) | <0.001 | <0.001 | 0.130 | 38% | 0.804 | 0.61-1.02 | N |
|  | Hu D, 2014 [23] | | | | high versus low intake | Jan 2014 | 3.09-37.0 | NOS | 24 | 706629 | 16981 | RR:0.79 (0.75-0.84) | 0.80 (0.76-0.83) | <0.001 | <0.001 | 0.233 | 17% | 0.050 | 0.70-0.89 | N |
|  | He F, 2006 [24] | | | | 3-5 servings/day | July, 2005 | 3.09-20.0 | NA | 9 | 257551 | 4917 | RR:0.89 (0.83-0.97) | 0.89 (0.84-0.94) | 0.005 | <0.001 | 0.151 | 33% | 0.832 | 0.75-1.06 | N |
|  | He F, 2006 [24] | | | | > 5 servings/day | July, 2005 | 3.09-20.0 | NA | 9 | 257551 | 4917 | RR:0.74 (0.69-0.79) | 0.74 (0.69-0.79) | <0.001 | <0.001 | 0.836 | 0% | 0.524 | 0.68-0.80 | N |
|  | Dauchet L, 2005 [25] | | | | per 1 serving/day | Sep 2004 | 3.1-20.0 | NA | 6 | 193612 | 1999 | RR:0.95 (0.92-0.97) | 0.95 (0.92-0.97) | <0.001 | <0.001 | 0.966 | 0% | 0.230 | 0.92-0.99 | Y |
| fruits | | | | | | | | | | | | | | | | | | | | |
|  | Aune D, 2017 [22] | | | | high versus low intake | Sep 2016 | NA | NA | 17 | 960337 | 46951 | RR:0.82 (0.77-0.87) | 0.80 (0.78-0.83) | <0.001 | <0.001 | 0.067 | 36% | 0.598 | 0.70-0.96 | N |
|  | Hu D, 2014 [23] | | | | high versus low intake | Jan 2014 | 3.09-37.0 | NOS | 19 | 703999 | 15655 | RR:0.77 (0.71-0.84) | 0.80 (0.76-0.84) | <0.001 | <0.001 | 0.004 | 52% | 0.017 | 0.59-1.01 | N |
|  | Dauchet L, 2005 [25] | | | | per 1 serving/day | Sep 2004 | 3.1-20.0 | NA | 5 | 210601 | 1853 | RR:0.89 (0.85-0.93) | 0.89 (0.85-0.93) | <0.001 | <0.001 | 0.635 | 0% | 0.869 | 0.84-0.95 | N |
|  | Bechthold A, 2019 [19] | | | | high versus low intake | Mar 2017 | 3.09-24.0 | NutriGrade | 17 | 881004 | 31111 | RR:0.83 (0.77-0.89) | 0.85 (0.81-0.89) | <0.001 | <0.001 | 0.052 | 39% | 0.083 | 0.69-1.003 | N |
|  | Bechthold A, 2019 [19] | | | | per 100 g/day | Mar 2017 | 3.09-24.0 | NutriGrade | 10 | 670409 | 26409 | RR:0.90 (0.84-0.97) | 0.93 (0.91-0.95) | <0.001 | <0.001 | <0.001 | 87% | 0.355 | 0.71-1.14 | Y |
| vegetables | | | | | | | | | | | | | | | | | | | | |
|  | Aune D, 2017 [22] | | | | high versus low intake | Sep 2016 | NA | NA | 13 | 427124 | 14519 | RR:0.87 (0.81-0.95) | 0.87 (0.82-0.92) | 0.002 | <0.001 | 0.079 | 38% | 0.815 | 0.71-1.07 | N |
|  | Hu D, 2014 [23] | | | | high versus low intake | Jan 2014 | 3.09-37.0 | NOS | 16 | 685887 | 14803 | RR:0.86 (0.79-0.93) | 0.85 (0.80-0.89) | <0.001 | <0.001 | 0.048 | 40% | 0.784 | 0.69-1.07 | N |
|  | Dauchet L, 2005 [25] | | | | per 1 serving/day | Sep 2004 | 3.1-20.0 | NA | 4 | 172164 | 933 | RR:0.97 (0.92-1.02) | 0.97 (0.92-1.02) | 0.193 | 0.193 | 0.403 | 0% | 0.179 | 0.85-1.11 | N |
|  | Bechthold A, 2019 [19] | | | | high versus low intake | Mar 2017 | 3.09-24.0 | NutriGrade | 16 | 442317 | 12442 | RR:0.87 (0.82-0.93) | 0.89 (0.85-0.92) | <0.001 | <0.001 | 0.056 | 39% | 0.400 | 0.74-1.02 | N |
|  | Bechthold A, 2019 [19] | | | | per 100 g/day | Mar 2017 | 3.09-24.0 | NutriGrade | 10 | 242345 | 8123 | RR:0.92 (0.86-0.98) | 0.96 (0.94-0.98) | 0.012 | <0.001 | <0.001 | 80% | 0.120 | 0.75-1.12 | Y |
| potato | | | | | | | | | | | | | | | | | | | | |
|  | Mazidi M, 2020 [26] | | | | high versus low intake | Jun 2018 | 6.4-13.0 | NOS | 3 | 94169 | NA | HR:0.94 (0.86-1.04) | 0.94 (0.86-1.04) | 0.221 | 0.214 | 0.357 | 3% | 0.946 | 0.50-1.79 | N |
|  | Schwingshackl L, 2019 [27] | | | | high versus low intake | May 2018 | 8.0-24.0 | NutriGrade | 6 | 213969 | 6902 | RR:0.98 (0.89-1.08) | 0.97 (0.90-1.06) | 0.671 | 0.533 | 0.238 | 26% | 0.508 | 0.78-1.23 | N |
|  | Schwingshackl L, 2019 [27] | | | | per 150 g/day | May 2018 | 8.0-24.0 | NutriGrade | 6 | 213969 | 6902 | RR:0.98 (0.93-1.03) | 0.98 (0.93-1.03) | 0.431 | 0.408 | 0.403 | 2% | 0.162 | 0.91-1.06 | Y |
| fish | | | | | | | | | | | | | | | | | | | | |
|  | Zhao W, 2019 [28] | | | | high versus low intake | Mar 2018 | NA | NutriGrade | 40 | 1101738 | 32369 | HR:0.91 (0.85-0.96) | 0.93 (0.89-0.96) | 0.001 | <0.001 | 0.007 | 39% | 0.084 | 0.74-1.12 | N |
|  | Xun P, 2012 [29] | | | | high versus low intake | Jan 2014 | 4.0-20.0 | NA | 17 | 402127 | 10568 | HR:0.91 (0.85-0.98) | 0.92 (0.87-0.97) | 0.013 | 0.003 | 0.235 | 19% | 0.302 | 0.78-1.06 | N |
|  | Larsson S, 2011 [30] | | | | per 3 servings/week | May 2011 | NA | NA | 18 | 383838 | 9360 | RR:0.94 (0.89-0.99) | 0.94 (0.90-0.98) | 0.025 | 0.003 | 0.159 | 25% | 0.866 | 0.83-1.07 | Y |
|  | He K, 2004 [31] | | | | high versus low intake | Oct 2003 | 4.0-30.0 | NA | 9 | 200575 | 3491 | RR:0.80 (0.67-0.96) | 0.82 (0.72-0.94) | 0.016 | 0.004 | 0.131 | 36% | 0.289 | 0.52-1.23 | N |
|  | Chen C, 2021 [32] | | | | high versus low intake | May 2019 | 3.0-20.0 | NOS | 10 | 455100 | 5581 | RR:0.87 (0.78-0.98） | 0.87 (0.78-0.98) | 0.016 | 0.016 | 0.616 | 0% | 0.048 | 0.76-0.996 | N |
|  | Bechthold A, 2019 [19] | | | | high versus low intake | Mar 2017 | 4.0-30.0 | NutriGrade | 20 | 432400 | 14360 | RR:0.95 (0.89-1.01) | 0.97 (0.93-1.02) | 0.115 | 0.225 | 0.053 | 37% | 0.067 | 0.79-1.14 | N |
|  | Bechthold A, 2019 [19] | | | | per 100 g/day | Mar 2017 | 4.0-30.0 | NutriGrade | 15 | 370844 | 11326 | RR:0.86 (0.75-0.99) | 0.86 (0.78-0.96) | 0.039 | 0.007 | 0.139 | 29% | 0.571 | 0.61-1.21 | N |
| fatty fish | | | | | | | | | | | | | | | | | | | | |
|  | Qin Z, 2018 [33] | | | | high versus low intake | Feb 2018 | 4.3-18.0 | NOS | 7 | 123681 | 3066 | RR:0.88 (0.74-1.04) | 0.87 (0.76-1.01) | 0.126 | 0.062 | 0.231 | 26% | 0.884 | 0.61-1.27 | Y |
| lean fish | | | | | | | | | | | | | | | | | | | | |
|  | Qin Z, 2018 [33] | | | | high versus low intake | Feb 2018 | 4.3-18.0 | NOS | 5 | 99369 | 2645 | RR:0.81 (0.67-0.99) | 0.81 (0.67-0.99) | 0.039 | 0.039 | 0.598 | 0% | 0.316 | 0.59-1.11 | Y |
| meat | | | | | | | | | | | | | | | | | | | | |
|  | Kim K, 2017 [34] | | | | high versus low intake | Oct 2016 | 5.5-26.0 | NOS | 6 | 213722 | 8848 | RR:1.18 (1.09-1.28) | 1.18 (1.09-1.28) | <0.001 | <0.001 | 0.725 | 0% | 0.098 | 1.05-1.32 | N |
|  | Chen G, 2013 [35] | | | | high versus low intake | Jun 2012 | 10.1-26.0 | NA | 4 | 202121 | 8131 | RR:1.15 (1.05-1.25) | 1.15 (1.05-1.25) | 0.002 | 0.002 | 0.876 | 0% | 0.301 | 0.95-1.39 | N |
|  | Micha R, 2010 [36] | | | | per 100 g/day | Mar 2009 | 3.0-18.0 | Micha scoring | 2 | 115500 | 931 | RR:1.17 (0.89-1.55) | 1.24 (1.08-1.43) | 0.269 | 0.003 | 0.137 | 55% | NA | NA | Y |
| red meat | | | | | | | | | | | | | | | | | | | | |
|  | Kim K, 2017 [34] | | | | high versus low intake | Oct 2016 | 5.5-26.0 | NOS | 8 | 254742 | 9522 | RR:1.12 (1.03-1.20) | 1.12 (1.03-1.20) | 0.006 | 0.006 | 0.432 | 0% | 0.593 | 1.02-1.23 | N |
|  | Yang C, 2016 [37] | | | | high versus low intake | Apr 2013 | NA | NOS | 5 | 242470 | 9581 | RR:1.14 (1.06-1.24) | 1.14 (1.06-1.24) | 0.001 | 0.001 | 0.504 | 0% | 0.689 | 1.00-1.30 | N |
|  | Chen G, 2013 [35] | | | | high versus low intake | Jun 2012 | 10.1-26.0 | NA | 5 | 239251 | 9593 | RR:1.09 (1.01-1.18) | 1.09 (1.01-1.18) | 0.030 | 0.030 | 0.920 | 0% | 0.624 | 0.96-1.27 | N |
|  | Kaluza J, 2012 [38] | | | | per 1 serving/day | May 2012 | 11.0-26.0 | NA | 5 | 289146 | 9168 | RR:1.11 (1.06-1.16) | 1.11 (1.06-1.16) | <0.001 | <0.001 | 0.592 | 0% | 0.097 | 1.03-1.19 | N |
|  | Micha R, 2010 [36] | | | | per 100 g/day | Mar 2009 | 3.0-18.0 | Micha scoring | 2 | 108898 | 1700 | RR:1.21 (1.10-1.33) | 1.21 (1.10-1.33) | <0.001 | <0.001 | 0.408 | 0% | NA | NA | N |
|  | Bechthold A, 2019 [19] | | | | high versus low intake | Mar 2017 | 7.6-26.0 | NutriGrade | 7 | 341767 | 10541 | RR:1.16 (1.08-1.25) | 1.16 (1.08-1.25) | <0.001 | <0.001 | 0.690 | 0% | 0.59 | 1.05-1.28 | N |
|  | Bechthold A, 2019 [19] | | | | per 100 g/day | Mar 2017 | 7.6-26.0 | NutriGrade | 7 | 341767 | 10541 | RR:1.12 (1.06-1.18) | 1.12 (1.06-1.18) | <0.001 | <0.001 | 0.479 | 0% | 0.422 | 1.04-1.20 | Y |
| processed meat | | | | | | | | | | | | | | | | | | | | |
|  | Kim K, 2017 [34] | | | | high versus low intake | Oct 2016 | 5.5-26.0 | NOS | 8 | 254742 | 9522 | RR:1.17 (1.08-1.25) | 1.17 (1.08-1.25) | <0.001 | <0.001 | 0.510 | 0% | 0.301 | 1.07-1.28 | N |
|  | Chen G, 2013 [35] | | | | high versus low intake | Jun 2012 | 10.1-26.0 | NA | 5 | 239251 | 9593 | RR:1.14 (1.05-1.25) | 1.15 (1.06-1.24) | 0.004 | <0.001 | 0.267 | 23% | 0.498 | 0.92-1.41 | N |
|  | Kaluza J, 2012 [38] | | | | per 1 serving/day | May 2012 | 11.0-26.0 | NA | 5 | 242470 | 9581 | RR:1.13 (1.03-1.24) | 1.10 (1.04-1.16) | 0.010 | 0.001 | 0.169 | 38% | 0.259 | 0.88-1.45 | N |
|  | Micha R, 2010 [36] | | | | per 50 g/day | Mar 2009 | 3.0-18.0 | Micha scoring | 2 | 108898 | 1434 | RR:1.14 (0.94-1.39) | 1.14 (0.94-1.39) | 0.183 | 0.183 | 0.902 | 0% | NA | NA | N |
|  | Bechthold A, 2019 [19] | | | | high versus low intake | Mar 2017 | 10.1-26.0 | NutriGrade | 6 | 254742 | 9492 | RR:1.16 (1.07-1.26) | 1.16 (1.08-1.25) | <0.001 | <0.001 | 0.332 | 13% | 0.538 | 0.99-1.35 | N |
|  | Bechthold A, 2019 [19] | | | | per 50 g/day | Mar 2017 | 10.1-26.0 | NutriGrade | 6 | 254742 | 9492 | RR:1.17 (1.02-1.34) | 1.14 (1.06-1.23) | 0.024 | <0.001 | 0.044 | 56% | 0.349 | 0.80-1.71 | Y |
| processed red meat | | | | | | | | | | | | | | | | | | | | |
|  | Yang C, 2016 [37] | | | | high versus low intake | Apr 2013 | NA | NOS | 5 | 242470 | 9581 | RR:1.17 (1.09-1.27) | 1.17 (1.09-1.27) | <0.001 | <0.001 | 0.464 | 0% | 0.362 | 1.03-1.33 | Y |
| fresh red meat | | | | | | | | | | | | | | | | | | | | |
|  | Yang C, 2016 [37] | | | | high versus low intake | Apr 2013 | NA | NOS | 5 | 242470 | 9581 | RR:1.10 (1.02-1.19) | 1.10 (1.02-1.19) | 0.018 | 0.018 | 0.826 | 0% | 0.678 | 0.97-1.25 | N |
|  | Kaluza J, 2012 [38] | | | | per 1 serving/day | May 2012 | 3.0-18.0 | NA | 6 | 329495 | 10630 | RR:1.11 (1.03-1.20) | 1.11 (1.03-1.20) | 0.008 | 0.008 | 0.648 | 0% | 0.758 | 0.996-1.24 | Y |
| white meat (poultry) | | | | | | | | | | | | | | | | | | | | |
|  | Kim K, 2017 [34] | | | | high versus low intake | Oct 2016 | 5.5-26.0 | NOS | 4 | 138761 | 4759 | RR:0.87 (0.78-0.96) | 0.87 (0.78-0.96) | 0.008 | 0.008 | 0.512 | 0% | 0.903 | 0.69-1.09 | N |
|  | Mohammadi H, 2018 [39] | | | | high versus low intake | Sep 2017 | 5.5-26.0 | NOS | 7 | 354718 | 7705 | RR:0.96 (0.84-1.09) | 0.92 (0.84-1.01) | 0.503 | 0.084 | 0.142 | 38% | 0.129 | 0.70-1.31 | N |
|  | Mohammadi H, 2018 [39] | | | | per 1 serving/week | Sep 2017 | 5.5-26.0 | NOS | 7 | 354718 | 7705 | RR:1.00 (0.96-1.03) | 1.00 (0.98-1.02) | 0.831 | 0.734 | 0.004 | 69% | 0.921 | 0.90-1.11 | Y |
| eggs | | | | | | | | | | | | | | | | | | | | |
|  | Tang H, 2020 [40] | | | | high versus low intake | Jul 2020 | 8.8-32.0 | NutriGrade | 21 | 1387653 | NA | RR:0.92 (0.84-1.01) | 0.92 (0.88-0.95) | 0.095 | <0.001 | 0.000 | 64% | 0.927 | 0.69-1.23 | N |
|  | Xu L, 2019 [41] | | | | high versus low intake | Mar 2012 | average 9.8 | NA | 14 | 402022 | 13306 | HR:0.91 (0.85-0.98) | 0.91 (0.85-0.98) | 0.012 | 0.012 | 0.523 | 0% | 0.563 | 0.84-0.99 | N |
|  | Mazidi M, 2019 [42] | | | | high versus low intake | Dec 2017 | 8.8-22.80 | NA | 8 | 94597 | NA | HR:0.72 (0.54-0.96) | 0.80 (0.68-0.93) | 0.026 | 0.005 | 0.127 | 38% | 0.268 | 0.37-1.39 | N |
|  | Shin J, 2013 [43] | | | | high versus low intake | Mar 2012 | 6.0-20.0 | MOOSE | 5 | 241900 | 4189 | HR:0.93 (0.81-1.07) | 0.93 (0.81-1.07) | 0.306 | 0.306 | 0.890 | 0% | 0.089 | 0.74-1.167 | N |
|  | Krittanawong C, 2021 [44] | | | | high versus low intake | Jan 2020 | median12.3 | NOS | 11 | 879242 | 40864 | HR:0.92 (0.84-1.02) | 0.89 (0.86-0.93) | 0.127 | <0.001 | 0.005 | 61% | 0.261 | 0.71-1.20 | N |
|  | Rong Y, 2013 [45] | | | | per 1 egg/day | Jun 2012 | 8.8-22.0 | NOS | 8 | 217167 | 7579 | RR:0.95 (0.88-1.02) | 0.95 (0.88-1.02) | 0.149 | 0.149 | 0.581 | 0% | 0.854 | 0.87-1.04 | N |
|  | Bechthold A, 2019 [19] | | | | high versus low intake | Mar 2017 | 7.6-26.0 | NutriGrade | 10 | 352448 | 12735 | RR:0.99 (0.93-1.05) | 0.99 (0.93-1.05) | 0.695 | 0.733 | 0.425 | 2% | 0.326 | 0.92-1.07 | N |
|  | Bechthold A, 2019 [19] | | | | per 50 g/day | Mar 2017 | 7.6-26.0 | NutriGrade | 10 | 352448 | 12735 | RR:0.99 (0.93-1.05) | 0.99 (0.93-1.05) | 0.717 | 0.717 | 0.442 | 0% | 0.575 | 0.92-1.06 | Y |
| legumes | | | | | | | | | | | | | | | | | | | | |
|  | Becerra-Tomás N, 2019 [46] | | | | high versus low intake | Oct 2017 | 7.4-24.0 | NA | 8 | 342079 | 8570 | RR:0.98 (0.87-1.11) | 0.96 (0.90-1.03) | 0.767 | 0.227 | 0.010 | 62% | 0.597 | 0.69-1.39 | N |
|  | Marventano S, 2017 [47] | | | | high versus low intake | Dec 2016 | 10.6-26.0 | NOS | 8 | 266241 | 6336 | RR:1.01 (0.89-1.14) | 0.98 (0.91-1.04) | 0.909 | 0.474 | 0.018 | 59% | 0.530 | 0.72-1.41 | N |
|  | Afshin A, 2014 [48] | | | | per 4 servings/week | Dec 2013 | 10.6-26.0 | Micha scoring | 8 | 254628 | 6690 | RR:0.98 (0.84-1.14) | 1.00 (0.90-1.12) | 0.786 | 0.956 | 0.185 | 34% | 0.009 | 0.68-1.41 | Y |
|  | Shi Z, 2014 [49] | | | | high versus low intake | Jan 2014 | 6.3-26.0 | NOS | 8 | 173229 | 4030 | RR:0.95 (0.84-1.08) | 0.98 (0.90-1.07) | 0.437 | 0.649 | 0.091 | 43% | 0.424 | 0.70-1.30 | N |
|  | Bechthold A, 2019 [19] | | | | high versus low intake | Mar 2017 | 10.6-26.0 | NutriGrade | 6 | 206756 | 6336 | RR:0.98 (0.88-1.10) | 0.98 (0.91-1.05) | 0.774 | 0.502 | 0.046 | 56% | 0.555 | 0.40-2.40 | N |
|  | Bechthold A, 2019 [19] | | | | per 50 g/day | Mar 2017 | 10.6-26.0 | NutriGrade | 6 | 206756 | 6336 | RR:1.00 (0.88-1.13) | 0.97 (0.92-1.05) | 0.999 | 0.527 | 0.022 | 62% | 0.852 | 0.70-1.44 | N |
| soy | | | | | | | | | | | | | | | | | | | | |
|  | Yan Z, 2017 [50] | | | | high versus low intake | Feb 2016 | 5.4-16.0 | NOS | 9 | 351226 | 6453 | RR:1.00 (0.88-1.14) | 1.06 (0.98-1.15) | 0.961 | 0.173 | 0.050 | 48% | 0.042 | 0.71-1.42 | Y |
|  | Lou D, 2016 [51] | | | | high versus low intake | May 2015 | 6.3-14.7 | NOS | 4 | 119884 | 2032 | RR:0.92 (0.77-1.10) | 0.94 (0.82-1.08) | 0.371 | 0.374 | 0.236 | 29% | 0.517 | 0.63-1.34 | N |
| nut | | | | | | | | | | | | | | | | | | | | |
|  | Becerra-Tomás N, 2019 [52] | | | | high versus low intake | Jun 2018 | 4.3-28.7 | NOS | 7 | 302888 | 12646 | RR:1.00 (0.92-1.09) | 1.00 (0.92-1.09) | 0.954 | 0.954 | 0.967 | 0% | 0.408 | 0.90-1.12 | N |
|  | Chen G, 2017 [53] | | | | high versus low intake | Jun 2015 | 4.3-30.0 | NOS | 12 | 449293 | 4398 | RR:0.82 (0.73-0.91) | 0.82 (0.73-0.91) | <0.001 | <0.001 | 0.818 | 0% | 0.384 | 0.72-0.90 | N |
|  | Shao C, 2016 [54] | | | | high versus low intake | Feb 2016 | 4.4-30.0 | NOS | 16 | 671301 | 7665 | RR:0.88 (0.81-0.97) | 0.88 (0.81-0.97) | 0.009 | 0.009 | 0.529 | 0% | 0.932 | 0.80-0.97 | N |
|  | Mayhew A, 2016 [55] | | | | high versus low intake | Jul 2015 | 4.6-30.0 | NOS | 2 | 153445 | 4318 | RR:1.05 (0.69-1.61) | 0.91 (0.82-1.01) | 0.819 | 0.065 | 0.037 | 77% | NA | NA | N |
|  | Aune D, 2016 [56] | | | | high versus low intake | Jul 2016 | 4.3-30.0 | NOS | 10 | 396768 | 9272 | RR:0.89 (0.82-0.97) | 0.89 (0.82-0.97) | 0.008 | 0.008 | 0.901 | 0% | 0.832 | 0.81-0.98 | N |
|  | Zhang Z, 2015 [57] | | | | high versus low intake | Jun 2014 | 7.6-30.0 | NOS | 9 | 476181 | NA | RR:0.90 (0.83-0.98) | 0.90 (0.83-0.98) | 0.011 | 0.011 | 0.751 | 0% | 0.397 | 0.81-0.995 | N |
|  | Zhou D, 2014 [58] | | | | high versus low intake | Oct 2013 | 3.8-26.0 | NA | 4 | 182730 | 5669 | RR:0.87 (0.73-1.05) | 0.87 (0.74-1.03) | 0.138 | 0.112 | 0.317 | 15% | 0.550 | 0.52-1.45 | N |
|  | Zhou D, 2014 [58] | | | | per 1 serving/day | Oct 2013 | 3.8-26.0 | NA | 4 | 182730 | 5669 | RR:0.90 (0.71-1.14) | 0.94 (0.82-1.09) | 0.381 | 0.437 | 0.114 | 50% | 0.273 | 0.51-1.58 | N |
|  | Shi Z, 2013 [49] | | | | high versus low intake | Jan 2014 | 6.3-26.0 | NOS | 4 | 228799 | 5669 | RR:0.90 (0.81-0.99) | 0.90 (0.81-0.99) | 0.027 | 0.027 | 0.527 | 0% | 0.858 | 0.72-1.12 | N |
|  | Luo C, 2014 [59] | | | | high versus low intake | Mar 2013 | 4.0-30.0 | MOOSE | 5 | 235263 | 6487 | RR:0.91 (0.81-1.02) | 0.90 (0.82-0.99) | 0.117 | 0.029 | 0.285 | 20% | 0.109 | 0.69-1.19 | N |
|  | Chen G, 2017 [53] | | | | per 1 serving/week | Jun 2015 | 4.3-30.0 | NOS | 12 | 432352 | 4831 | RR:0.95 (0.91-0.999) | 0.98 (0.96-1.00) | 0.044 | 0.055 | 0.005 | 59% | 0.005 | 0.84-1.08 | Y |
|  | Bechthold A, 2019 [19] | | | | high versus low intake | Mar 2017 | 7.6-26.0 | NutriGrade | 6 | 273149 | 7490 | RR:0.94 (0.85-1.05) | 0.93 (0.85-1.02) | 0.266 | 0.115 | 0.3 | 18% | 0.033 | 0.76-1.16 | N |
|  | Bechthold A, 2019 [19] | | | | per 28 g/day | Mar 2017 | 7.6-26.0 | NutriGrade | 6 | 273149 | 7490 | RR:0.99 (0.84-1.17) | 1.00 (0.88-1.12) | 0.886 | 0.965 | 0.107 | 45% | 0.865 | 0.63-1.55 | N |
| peanut | | | | | | | | | | | | | | | | | | | | |
|  | Becerra-Tomás N, 2019 [52] | | | | high versus low intake | Jun 2018 | 4.3-28.7 | NOS | 3 | 210836 | 5910 | RR:0.89 (0.79-1.00） | 0.89 (0.80-0.995) | 0.005 | 0.039 | 0.322 | 12% | 0.162 | 0.36-2.19 | N |
|  | Chen G, 2017 [53] | | | | high versus low intake | Jun 2015 | 4.3-30.0 | NOS | 5 | 265252 | 3315 | RR:0.83 (0.71-0.97) | 0.82 (0.73-0.92) | 0.019 | 0.001 | 0.115 | 46% | 0.351 | 0.52-1.32 | N |
|  | Aune D, 2016 [56] | | | | high versus low intake | Jul 2016 | 4.3-30.0 | NOS | 5 | 265252 | 3315 | RR:0.83 (0.69-0.995) | 0.83 (0.73-0.95) | 0.044 | 0.005 | 0.116 | 46% | 0.945 | 0.49-1.41 | N |
|  | Aune D, 2016 [56] | | | | per 10 g/day | Jul 2016 | 4.3-30.0 | NOS | 5 | 265252 | 3315 | RR:0.63 (0.42-0.96) | 0.72 (0.60-0.87) | 0.030 | 0.001 | 0.002 | 77% | 0.232 | 0.15-2.67 | N |
|  | Chen G, 2017 [53] | | | | per 1 serving/week | Jun 2015 | 4.3-30.0 | NOS | 5 | 265252 | 3315 | RR:0.84 (0.73-0.96) | 0.88 (0.82-0.94) | 0.012 | <0.001 | 0.007 | 72% | 0.189 | 0.53-1.33 | Y |
| tree nut | | | | | | | | | | | | | | | | | | | | |
|  | Becerra-Tomás N, 2019 [52] | | | | high versus low intake | Jun 2018 | 4.3-28.7 | NOS | 3 | 210836 | 5910 | RR:1.00 (0.89-1.11) | 1.00 (0.89-1.11) | 0.963 | 0.963 | 0.924 | 0% | 0.570 | 0.49-2.04 | N |
|  | Chen G, 2017 [53] | | | | high versus low intake | Jun 2015 | 4.3-30.0 | NOS | 3 | 130987 | 2130 | RR:0.93 (0.77-1.13) | 0.93 (0.77-1.13) | 0.469 | 0.469 | 0.443 | 0% | 0.748 | 0.27-3.22 | N |
|  | Aune D, 2016 [56] | | | | high versus low intake | Jul 2016 | 4.3-30.0 | NOS | 3 | 130987 | 2130 | RR:0.93 (0.77-1.13) | 0.93 (0.77-1.13) | 0.469 | 0.469 | 0.443 | 0% | 0.748 | 0.27-3.22 | N |
|  | Aune D, 2016 [56] | | | | per 10 g/day | Jul 2016 | 4.3-30.0 | NOS | 3 | 130987 | 2130 | RR:0.89 (0.70-1.14) | 0.89 (0.70-1.14) | 0.361 | 0.361 | 0.574 | 0% | 0.513 | 0.18-4.33 | N |
|  | Chen G, 2017 [53] | | | | per 1 serving/week | Jun 2015 | 4.3-30.0 | NOS | 3 | 130987 | 2130 | RR:0.96 (0.87-1.06) | 0.96 (0.87-1.06) | 0.374 | 0.374 | 0.566 | 0% | 0.529 | 0.51-1.82 | Y |
| walnut | | | | | | | | | | | | | | | | | | | | |
|  | Becerra-Tomás N, 2019 [52] | | | | high versus low intake | Jun 2018 | 4.3-28.7 | NOS | 3 | 144021 | 5910 | RR:0.86 (0.68-1.09) | 0.85 (0.71-1.02) | 0.211 | 0.082 | 0.192 | 40% | 0.536 | 0.09-8.20 | Y |
| peanut butter | | | | | | | | | | | | | | | | | | | | |
|  | Becerra-Tomás N, 2019 [52] | | | | high versus low intake | Jun 2018 | 4.3-28.7 | NOS | 3 | 210836 | 5910 | RR:0.90 (0.73-1.12) | 0.94 (0.87-1.02) | 0.357 | 0.117 | 0.001 | 86% | 0.269 | 0.07-12.40 | Y |
| nut plus peanut butter | | | | | | | | | | | | | | | | | | | | |
|  | Chen G, 2017 [53] | | | | high versus low intake | Jun 2015 | 4.3-28.7 | NOS | 3 | 104531 | 924 | RR:0.84 (0.70-1.01) | 0.84 (0.70-1.01) | 0.066 | 0.066 | 0.659 | 0% | 0.609 | 0.26-2.76 | Y |
| dairy products | | | | | | | | | | | | | | | | | | | | |
|  | Gholami F, 2017 [60] | | | | high versus low intake | Sep 2014 | 10.0-65.0 | STROBE | 19 | 764917 | 29300 | RR:0.88 (0.82-0.95) | 0.87 (0.84-0.89) | 0.001 | <0.001 | 0.000 | 63% | 0.692 | 0.70-1.11 | N |
|  | Alexander D, 2016 [61] | | | | high versus low intake | Mar 2015 | 5.0-26.0 | NA | 9 | 286474 | NA | RR:0.91 (0.83-0.99） | 0.92 (0.87-0.98) | 0.037 | 0.006 | 0.072 | 45% | 0.364 | 0.73-1.14 | N |
|  | Qin L, 2015 [62] | | | | NA | Feb 2014 | 8.0-26.0 | MOOSE | 12 | 504803 | 21801 | RR:0.87 (0.77-0.99) | 0.84 (0.81-0.88) | 0.032 | <0.001 | 0.000 | 70% | 0.558 | 0.61-1.25 | N |
|  | Elwood P, 2008 [63] | | | | high versus low intake | Jun 2008 | 10.0-68.0 | NA | 7 | 414097 | 14358 | RR:0.80 (0.73-0.86) | 0.79 (0.76-0.83) | <0.001 | <0.001 | 0.300 | 16% | 0.575 | 0.68-0.94 | N |
|  | Bechthold A, 2019 [19] | | | | high versus low intake | Mar 2017 | 7.6-26.0 | NutriGrade | 12 | 503792 | 16887 | RR:0.96 (0.90-1.02) | 0.96 (0.92-1.00) | 0.146 | 0.031 | 0.051 | 44% | 0.534 | 0.82-1.12 | N |
|  | Bechthold A, 2019 [19] | | | | per 200 g/day | Mar 2017 | 7.6-26.0 | NutriGrade | 11 | 544881 | 19450 | RR:0.98 (0.96-1.02) | 0.99 (0.98-1.00) | 0.074 | 0.17 | 0.029 | 50% | 0.166 | 0.92-1.04 | Y |
| milk | | | | | | | | | | | | | | | | | | | | |
|  | Gholami F, 2017 [64] | | | | high versus low intake | Sep 2014 | 10.0-65.0 | STROBE | 11 | 463318 | 22946 | RR:0.91 (0.81-1.01) | 0.86 (0.83-0.90) | 0.066 | <0.001 | 0.000 | 71% | 0.454 | 0.67-1.23 | N |
|  | Soedamah-Muthu, 2018 [65] | | | | per 200 g/day | Jul 2018 | median 18 | NA | 17 | 4381604 | 25377 | RR:0.92 (0.88-0.97) | 0.98 (0.97-0.998) | 0.003 | 0.024 | 0.000 | 85% | 0.025 | 0.77-1.11 | Y |
|  | Mullie P, 2016 [66] | | | | per 200 ml/day | Jun 2015 | median 16 | NA | 10 | 564717 | 39352 | RR:0.92 (0.85-0.99) | 0.99 (0.98-1.01) | 0.027 | 0.190 | 0.000 | 92% | 0.157 | 0.71-1.19 | N |
|  | de Goede J, 2016 [67] | | | | per 200 g/day | Oct 2015 | 8.0-26.0 | NOS | 14 | 603920 | 25269 | RR:0.93 (0.88-0.98) | 0.98 (0.97-0.998) | 0.004 | 0.025 | 0.000 | 86% | 0.029 | 0.77-1.12 | N |
|  | Soedamah-Muthu, 2011 [68] | | | | per 200ml/day | Feb 2010 | median 18 | NA | 6 | 375381 | 15554 | RR:0.88 (0.71-1.07) | 0.98 (0.95-1.00) | 0.202 | 0.065 | 0.000 | 95% | 0.323 | 0.43-1.82 | N |
| cheese | | | | | | | | | | | | | | | | | | | | |
|  | Gholami F, 2017 [64] | | | | high versus low intake | Sep 2014 | 10.0-65.0 | STROBE | 7 | 224101 | 10483 | RR:0.94 (0.88-0.99) | 0.94 (0.88-0.99) | 0.019 | 0.019 | 0.751 | 0% | 0.647 | 0.87-1.02 | N |
|  | Chen G, 2017 [69] | | | | high versus low intake | Dec 2015 | 10.0-15.0 | NOS | 6 | 257069 | 10449 | RR:0.90 (0.84-0.97) | 0.90 (0.84-0.97) | 0.003 | 0.003 | 0.418 | 0% | 0.117 | 0.81-0.997 | N |
|  | Chen G, 2017 [69] | | | | per 50 g/day | Dec 2015 | 10.0-15.0 | NOS | 5 | 171305 | 9759 | RR:0.94 (0.84-1.04) | 0.96 (0.92-0.998) | 0.212 | 0.038 | 0.026 | 64% | 0.179 | 0.68-1.29 | Y |
| cream | | | | | | | | | | | | | | | | | | | | |
|  | Gholami F, 2017 [64] | | | | high versus low intake | Sep 2014 | 10.0-65.0 | STROBE | 3 | 127962 | 8546 | RR:0.97 (0.88-1.06) | 0.97 (0.88-1.06) | 0.443 | 0.443 | 0.399 | 0% | 0.303 | 0.53-1.77 | Y |
| butter | | | | | | | | | | | | | | | | | | | | |
|  | Gholami F, 2017 [64] | | | | high versus low intake | Sep 2014 | 10.0-65.0 | STROBE | 4 | 111280 | 5299 | RR:0.95 (0.85-1.08) | 0.95 (0.85-1.08) | 0.439 | 0.439 | 0.571 | 0% | 0.891 | 0.73-1.24 | N |
|  | Pimpin L, 2016 [70] | | | | per 14 g/day | May 2015 | 10.0-13.6 | NOS | 3 | 173853 | 5229 | RR:1.01 (0.98-1.03) | 1.01 (0.98-1.03) | 0.620 | 0.620 | 0.754 | 0% | 0.396 | 0.86-1.19 | Y |
| yogurt | | | | | | | | | | | | | | | | | | | | |
|  | Wu L, 2017 [71] | | | | high versus low intake | Jan 2017 | 10.2-17.3 | NOS | 7 | 225141 | 7875 | RR:1.02 (0.92-1.14) | 0.99 (0.94-1.05) | 0.704 | 0.842 | 0.025 | 58% | 0.535 | 0.77-1.36 | Y |
| chocolate | | | | | | | | | | | | | | | | | | | | |
|  | Morze J, 2020 [72] | | | | high versus low intake | Jul 2018 | 8.7-16.0 | NutriGrade | 8 | 309559 | 11089 | RR:0.86 (076-0.96) | 0.90 (0.84-0.96) | 0.010 | 0.001 | 0.013 | 61% | 0.033 | 0.62-1.20 | N |
|  | Morze J, 2020 [72] | | | | per 10 g/day | Jul 2018 | 8.7-16.0 | NutriGrade | 7 | 275070 | 10620 | RR:0.90 (0.82-0.98) | 0.96 (0.92-0.998) | 0.019 | 0.040 | 0.024 | 59% | 0.002 | 0.71-1.15 | Y |
|  | Ren Y, 2019 [73] | | | | high versus low intake | Jun 2018 | median 11 | NOS | 8 | 231038 | 6595 | RR:0.84 (0.78-0.90) | 0.84 (0.78-0.90) | <0.001 | <0.001 | 0.490 | 0% | 0.008 | 0.77-0.92 | N |
|  | Gianfredi V, 2018 [74] | | | | high versus low intake | Sep 2016 | 8.0-16.0 | Buitrago-Lopez | 5 | 123482 | NA | RR:0.73 (0.63-0.86) | 0.76 (0.69-0.83) | <0.001 | <0.001 | 0.034 | 59% | 0.324 | 0.46-1.15 | N |
|  | Yuan S, 2017 [75] | | | | high versus low intake | Mar 2017 | 5.0-16.0 | NOS | 8 | 231038 | 8197 | RR:0.84 (0.78-0.90) | 0.84 (0.78-0.90) | <0.001 | <0.001 | 0.485 | 0% | 0.008 | 0.77-0.92 | N |
|  | Larsson S, 2012 [76] | | | | high versus low intake | Jan 2012 | 8.0-16.0 | NA | 5 | 131345 | 4260 | RR:0.81 (0.73-0.90) | 0.81 (0.73-0.90) | <0.001 | <0.001 | 0.467 | 0% | 0.025 | 0.68-0.96 | N |
|  | Buitrago-Lopez A, 2011 [77] | | | | high versus low intake | Oct 2010 | 8.0-16.0 | Buitrago-Lopez | 3 | 36128 | NA | RR:0.71 (0.52-0.98) | 0.79 (0.66-0.94) | 0.038 | 0.008 | 0.183 | 41% | 0.22 | 0.03-16.69 | N |
| coffee | | | | | | | | | | | | | | | | | | | | |
|  | Shao C, 2021 [78] | | | | high versus low intake | Aug 2020 | 3.5-28.0 | NOS | 27 | 2488086 | NA | RR:0.87 (0.80-0.94） | 0.84 (0.79-0.90) | 0.001 | <0.001 | 0.058 | 32% | 0.006 | 0.68-1.12 | Y |
|  | Kim B, 2012 [79] | | | | high versus low intake | Jul 2011 | 3.5-24.0 | NOS | 9 | 206437 | 8110 | RR:0.83 (0.76-0.91) | 0.83 (0.76-0.91) | <0.001 | <0.001 | 0.434 | 0% | 0.213 | 0.75-0.93 | N |
|  | Zhang R, 2012 [80] | | | | high versus low intake | Apr 2011 | 2.0-25.0 | NOS | 13 | 492760 | 12414 | RR:0.89 (0.81-0.97） | 0.86 (0.83-0.89) | 0.006 | <0.001 | 0.000 | 69% | 0.234 | 0.68-1.16 | N |
| tea | | | | | | | | | | | | | | | | | | | | |
|  | Zhang C, 2015 [81] | | | | high versus low intake | Jun 2014 | 3.8-24.0 | NOS | 9 | 307968 | 11329 | RR:0.67 (0.53-0.85) | 0.78 (0.72-0.85) | 0.001 | <0.001 | 0.000 | 73% | 0.134 | 0.33-1.38 | N |
|  | Chung M, 2020 [82] | | | | per 1 cup/day | Nov 2019 | 5.0-24.0 | NOS | 7 | 525478 | NA | RR:0.96 (0.94-0.99） | 0.96 (0.95-0.97) | 0.003 | <0.001 | 0.001 | 64% | 0.553 | 0.89-1.04 | Y |
|  | Zhang C, 2015 [81] | | | | per 3 cups/day | Jun 2014 | 3.8-24.0 | NOS | 9 | 307968 | 11329 | RR:0.82 (0.73-0.93) | 0.89 (0.85-0.93) | 0.001 | <0.001 | 0.000 | 78% | 0.177 | 0.57-1.19 | N |
|  | Shen L, 2012 [83] | | | | per 3 cups/day | Mar 2012 | median 12 | NA | 16 | 513804 | 10192 | RR:0.87 (0.81-0.94) | 0.87 (0.83-0.92) | 0.001 | <0.001 | 0.006 | 54% | 0.853 | 0.68-1.11 | N |
|  | Arab L, 2009 [84] | | | | high versus low intake | Oct 2007 | 4.0-15.0 | NA | 10 | 212632 | 5005 | RR:0.79 (0.72-0.86) | 0.79 (0.73-0.85) | <0.001 | <0.001 | 0.224 | 24% | 0.448 | 0.66-0.95 | N |
| sugar-sweetened beverages | | | | | | | | | | | | | | | | | | | | |
|  | Xi B, 2015 [85] | | | | high versus low intake | May 2014 | 10.0-28.0 | NOS | 4 | 259176 | 10011 | RR:1.10 (0.98-1.24) | 1.12 (1.03-1.22) | 0.113 | 0.012 | 0.190 | 37% | 0.396 | 0.73-1.65 | N |
|  | Narain A, 2016 [86] | | | | high versus low intake | Jul 2015 | 9.8-24.0 | Narain scoring | 6 | 235701 | NA | RR:1.02 (0.97-1.25) | 1.12 (1.02-1.23) | 0.145 | 0.014 | 0.082 | 49% | 0.328 | 0.71-1.46 | N |
|  | Narain A, 2016 [86] | | | | per 1 serving/day | Jul 2015 | 9.8-24.0 | Narain scoring | 2 | 127456 | NA | RR:1.13 (1.02-1.24) | 1.13 (1.02-1.24) | 0.016 | 0.016 | 0.638 | 0% | NA | NA | N |
|  | Bechthold A, 2019 [19] | | | | high versus low intake | Mar 2017 | 10.0-26.0 | NutriGrade | 7 | 264709 | 11187 | RR:1.09 (1.01-1.18) | 1.09 (1.01-1.18) | 0.034 | 0.34 | 0.425 | 0% | 0.831 | 0.98-1.20 | N |
|  | Bechthold A, 2019 [19] | | | | per 250 ml/day | Mar 2017 | 10.0-26.0 | NutriGrade | 6 | 238264 | 10011 | RR:1.07 (1.02-1.12) | 1.07 (1.02-1.12) | 0.003 | 0.003 | 0.586 | 0% | 0.453 | 1.00-1.14 | Y |
| artificially sweetened beverage | | | | | | | | | | | | | | | | | | | | |
|  | Narain A, 2016 [86] | | | | high versus low intake | Jul 2015 | 9.8-24.0 | Narain scoring | 2 | 127456 | NA | RR:1.14 (1.04-1.26) | 1.14 (1.04-1.26) | 0.007 | 0.007 | 0.463 | 0% | NA | NA | N |
|  | Narain A, 2016 [86] | | | | per 1 serving/day | Jul 2015 | 9.8-24.0 | Narain scoring | 2 | 127456 | NA | RR:1.08 (1.03-1.15) | 1.08 (1.03-1.15) | 0.004 | 0.004 | 0.764 | 0% | NA | NA | Y |

PI: prediction interval; NA: not available; Y: yes; N: no; NOS:Newcastle-Ottawa Scale; STROBE: Strengthening the Reporting of Observational Studies in Epidemiology; MOOSE: Meta-analysis Of Observational Studies in Epidemiology.
