# Supplementary material for: Role of diet in stroke incidence: an umbrella review of meta-analyses of prospective observational studies
Source: BMC Med. 2022 May 24;20:194. doi: 10.1186/s12916-022-02381-6 (PMC9128224; doi:10.1186/s12916-022-02381-6)
Supplement: Supplementary file 3 — Additional file 3: Table S3. Characteristics of included meta-analyses evaluating associations between food nutrients and stroke risk. [file 12916_2022_2381_MOESM3_ESM.docx]

|  | |  | |  | | |  | | | | | | | | | | | | |
| --- | --- | --- | --- | --- | --- | --- | --- | --- | --- | --- | --- | --- | --- | --- | --- | --- | --- | --- | --- |
|  | |  | |  | | |  | | | | | | | | | | | | |
|  | |  | |  | | |  | | | | | | | | | | | | |
| **Table S3.** **Characteristics of included meta-analyses evaluating associations between food nutrients and stroke risk.** | | | | | | | | | | | | | | | | | | | |
| **Dietary factor** | **Author, year** | **Comparison** | **Article retrieval time** | | **Duration of follow up (years)** | **Assessment tool of original study** | | **Studies** | **Subjects** | **Cases** | **Relative risk and 95% CIs** | | **Random *P* value** | **Fixed *P* value** | **Heterogeneity** | | **Small-study**  **effects** | **95%PI** | **Includedin main analysis** |
|  |  |  |  |  |  |  |  | **（n）** | **（n）** | **（n）** | **Random effects** | **Fixed effects** |  |  | ***P-value*** | ***I²*** |  |  |  |
| protein | | | | | | | | | | | | | | | | | | | |
|  | Zhang X, 2016 [87] | high versus low intake | Jul 2016 | | 5.0-26.0 | NOS | | 12 | 528982 | NA | RR:0.98 (0.89-1.07) | 1.03 (0.99-1.06) | 0.583 | 0.144 | 0.000 | 67% | 0.106 | 0.75-1.28 | Y |
|  | Zhang Z, 2014 [88] | high versus low intake | Nov 2013 | | 10.4-18.0 | NOS | | 7 | 254489 | NA | RR:0.81 (0.66-0.99) | 0.86 (0.79-0.94) | 0.038 | 0.001 | 0.017 | 61% | 0.236 | 0.42-1.39 | N |
| animal protein | | | | | | | | | | | | | | | | | | | |
|  | Zhang Z, 2014 [88] | high versus low intake | Nov 2013 | | 10.4-18.0 | NOS | | 5 | 172900 | NA | RR:0.71 (0.50-1.00) | 0.81 (0.70-0.94) | 0.049 | 0.006 | 0.011 | 69% | 0.297 | 0.24-2.14 | Y |
| plant protein | | | | | | | | | | | | | | | | | | | |
|  | Zhang Z, 2014 [88] | high versus low intake | Nov 2013 | | 10.4-18.0 | NOS | | 5 | 172900 | NA | RR:0.88 (0.76-1.02) | 0.88 (0.76-1.02) | 0.096 | 0.096 | 0.884 | 0% | 0.379 | 0.69-1.12 | Y |
| saturated fat | | | | | | | | | | | | | | | | | | | |
|  | Kang Z, 2020 [89] | high versus low intake | Dec 2018 | | 7.4-20.0 | NOS | | 14 | 598435 | 12074 | RR:0.87 (0.78-0.96) | 0.86 (0.80-0.93) | 0.005 | <0.001 | 0.075 | 38% | 0.358 | 0.67-1.14 | Y |
|  | Cheng P, 2016 [90] | high versus low intake | NA | | 7.6-23.0 | NOS | | 16 | 476569 | 11074 | RR:0.90 (0.81-1.00) | 0.88 (0.82-0.95) | 0.045 | 0.001 | 0.060 | 38% | 0.849 | 0.68-1.20 | N |
|  | Siri-Tarino P, 2010 [91] | NA | NA | | 5.0-23.0 | Siri-Tarino scoring | | 8 | 179436 | 2362 | RR:0.81 (0.62-1.05) | 0.84 (0.73-0.98) | 0.115 | 0.023 | 0.010 | 62% | 0.483 | 0.38-1.74 | N |
| MUFA | | | | | | | | | | | | | | | | | | | |
|  | Cheng P, 2016 [92] | high versus low intake | Jan 2016 | | 7.6-20.0 | NOS | | 10 | 314511 | 5827 | RR:0.86 (0.74-1.00) | 0.85 (0.77-0.93) | 0.047 | 0.001 | 0.029 | 49% | 0.721 | 0.57-1.31 | Y |
| n-3 PUFA | | | | | | | | | | | | | | | | | | | |
|  | Cheng P, 2015 [93] | high versus low intake | May 2015 | | 4.0-28.0 | NOS | | 17 | 514483 | 9065 | RR:0.87 (0.80-0.95) | 0.87 (0.81-0.94) | 0.002 | <0.001 | 0.276 | 15% | 0.253 | 0.73-1.03 | Y |
|  | Larsson S, 2012 [94] | high versus low intake | Nov 2012 | | 4.0-28.0 | NOS | | 10 | 242076 | 5238 | RR:0.90 (0.81-1.01) | 0.90 (0.82-0.99) | 0.061 | 0.023 | 0.302 | 15% | 0.897 | 0.73-1.10 | N |
|  | Chen C, 2021 [32] | high versus low intake | May 2019 | | 3.0-20.0 | NOS | | 15 | 400634 | 11709 | RR:0.86 (0.78-0.95) | 0.86 (0.80-0.93) | 0.002 | <0.001 | 0.094 | 34% | 0.449 | 0.66-1.13 | N |
| cholesterol | | | | | | | | | | | | | | | | | | | |
|  | Cheng P, 2018 [95] | high versus low intake | Sep 2017 | | 7.6-15.5 | NOS | | 16 | 269777 | 4604 | RR:0.95 (0.84-1.07) | 0.98 (0.90-1.07) | 0.386 | 0.713 | 0.063 | 38% | 0.100 | 0.68-1.32 | Y |
| α-linolenic acid | | | | | | | | | | | | | | | | | | | |
|  | Pan A, 2012 [96] | high versus low intake | Jan 2012 | | 10.4-13.0 | NOS | | 3 | 98410 | NA | RR:0.95 (0.77-1.18) | 1.00 (0.88-1.13) | 0.642 | 0.999 | 0.121 | 53% | 0.277 | 0.10-8.76 | Y |
| carbohydrate | | | | | | | | | | | | | | | | | | | |
|  | Cai X, 2015 [97] | high versus low intake | Mar 2014 | | 5.0-18.0 | NOS | | 6 | 170348 | 1851 | RR:1.12 (0.92-1.36) | 1.12 (0.93-1.35) | 0.258 | 0.236 | 0.376 | 6% | 0.819 | 0.81-1.56 | N |
|  | Mohammadifard, 2021 [98] | high versus low intake | Jun 2019 | | 7.0-28.0 | NOS | | 8 | 423049 | 6829 | RR:1.13 (1.01-1.27) | 1.13 (1.01-1.27) | 0.040 | 0.040 | 0.617 | 0% | 0.818 | 0.93-1.37 | Y |
| total fiber | | | | | | | | | | | | | | | | | | | |
|  | Zhang Z, 2013 [99] | high versus low intake | Feb 2013 | | 8.0-18.0 | NOS | | 14 | 325627 | 9676 | RR:0.83 (0.74-0.93) | 0.85 (0.79-0.92) | 0.001 | <0.001 | 0.069 | 39% | 0.064 | 0.62-1.12 | N |
|  | Chen G, 2013 [100] | high versus low intake | Jul 2012 | | 8.0-18.0 | NOS | | 9 | 314864 | 8920 | RR:0.88 (0.78-0.99) | 0.90 (0.82-0.98) | 0.032 | 0.017 | 0.132 | 36% | 0.285 | 0.66-1.18 | N |
|  | Threapleton D, 2013 [101] | per 7 g/day | May 2012 | | 8.0-19.0 | NA | | 7 | 324640 | 9836 | RR:0.93 (0.88-0.98) | 0.97 (0.94-0.99) | 0.006 | 0.013 | 0.02 | 60% | 0.001 | 0.80-1.08 | Y |
| soluble dietary fiber | | | | | | | | | | | | | | | | | | | |
|  | Zhang Z, 2013 [99] | high versus low intake | Feb 2013 | | 8.0-18.0 | NOS | | 5 | 173320 | 6978 | RR:0.79 (0.61-1.01) | 0.85 (0.75-0.95) | 0.062 | 0.005 | 0.011 | 69% | 0.326 | 0.34-1.84 | N |
|  | Threapleton D, 2013 [101] | per 4 g/day | May 2012 | | 8.0-19.0 | NA | | 3 | 95062 | 5192 | RR:0.94 (0.88-1.01) | 0.95 (0.90-0.999) | 0.076 | 0.046 | 0.306 | 16% | 0.793 | 0.54-1.64 | Y |
| insoluble dietary fiber | | | | | | | | | | | | | | | | | | | |
|  | Zhang Z, 2013 [99] | high versus low intake | Feb 2013 | | 8.0-18.0 | NOS | | 5 | 173320 | 6978 | RR:0.76 (0.57-1.03) | 0.88 (0.78-0.99) | 0.076 | 0.039 | 0.002 | 76% | 0.151 | 0.27-2.13 | Y |
| cereal fiber | | | | | | | | | | | | | | | | | | | |
|  | Zhang Z, 2013 [99] | high versus low intake | Feb 2013 | | 8.0-18.0 | NOS | | 4 | 111820 | 4824 | RR:0.76 (0.58-1.00) | 0.87 (0.78-0.96) | 0.049 | 0.005 | 0.001 | 82% | 0.133 | 0.23-2.50 | Y |
| fruit fiber | | | | | | | | | | | | | | | | | | | |
|  | Zhang Z, 2013 [99] | high versus low intake | Feb 2013 | | 8.0-18.0 | NOS | | 2 | 105335 | 4385 | RR:0.92 (0.83-1.02) | 0.92 (0.83-1.02) | 0.102 | 0.102 | 0.603 | 0% | NA | NA | Y |
| vegetable fiber | | | | | | | | | | | | | | | | | | | |
|  | Zhang Z, 2013 [99] | high versus low intake | Feb 2013 | | 8.0-18.0 | NOS | | 2 | 105335 | 4385 | RR:0.86 (0.77-0.95) | 0.86 (0.77-0.95) | 0.004 | 0.004 | 0.47 | 0% | NA | NA | Y |
| vitamin B6 | | | | | | | | | | | | | | | | | | | |
|  | Chen L, 2020 [102] | high versus low intake | Feb 20 20 | | 4.2-19.0 | NOS | | 10 | 264253 | 7334 | RR:0.84 (0.73-0.97) | 0.84 (0.78-0.92) | 0.018 | <0.001 | 0.041 | 49% | 0.969 | 0.58-1.21 | N |
|  | Chen L, 2020 [102] | per 0.5 mg/day | Feb 20 20 | | 4.2-19.0 | NOS | | 9 | 263498 | 7264 | RR:0.94 (0.89-0.99) | 0.996 (0.98-1.01) | 0.022 | 0.604 | 0.000 | 77% | 0.021 | 0.80-1.10 | Y |
| vitamin B12 | | | | | | | | | | | | | | | | | | | |
|  | Chen L, 2020 [102] | high versus low intake | Feb 20 20 | | 4.2-19.0 | NOS | | 10 | 130965 | 5458 | RR:1.02 (0.93-1.12) | 1.02 (0.93-1.12) | 0.655 | 0.655 | 0.455 | 0% | 0.686 | 0.91-1.14 | N |
|  | Chen L, 2020 [102] | per 3 μg/day | Feb 20 20 | | 4.2-19.0 | NOS | | 7 | 129018 | 4992 | RR:1.01 (0.98-1.06) | 1.00 (0.98-1.03) | 0.477 | 0.880 | 0.117 | 41% | 0.114 | 0.92-1.11 | Y |
| vitamin C | | | | | | | | | | | | | | | | | | | |
|  | Chen G, 2013 [103] | high versus low intake | Apr 2013 | | 9.5-20.0 | NA | | 11 | 138051 | 3612 | RR:0.82 (0.74-0.90) | 0.82 (0.74-0.90) | <0.001 | <0.001 | 0.707 | 0% | 0.387 | 0.73-0.92 | N |
|  | Chen G, 2013 [103] | per 100 mg/day | Apr 2013 | | 9.5-20.0 | NA | | 10 | 132892 | 3385 | RR:0.84 (0.75-0.93) | 0.83 (0.76-0.91) | 0.001 | <0.001 | 0.320 | 13% | 0.945 | 0.70-1.01 | Y |
|  | Aune D, 2018 [104] | high versus low intake | Feb 2018 | | 5.8-30.0 | NOS | | 13 | 298066 | 7294 | RR:0.84 (0.77-0.91) | 0.84 (0.78-0.91) | <0.001 | <0.001 | 0.390 | 6% | 0.945 | 0.75-0.95 | N |
| vitamin D | | | | | | | | | | | | | | | | | | | |
|  | Shi H, 2020 [105] | high versus low intake | 2018 | | 1.0-34.0 | NOS | | 4 | 67238 | 2616 | RR:0.75 (0.57-0.98) | 0.77 (0.66-0.89) | 0.037 | 0.001 | 0.118 | 49% | 0.879 | 0.28-2.03 | Y |
| vitamin E | | | | | | | | | | | | | | | | | | | |
|  | Cheng P, 2018 [106] | high versus low intake | Jun 2018 | | 6.1-16.5 | NOS | | 11 | 186183 | 2956 | RR:0.82 (0.71-0.95) | 0.83 (0.73-0.94) | 0.009 | 0.003 | 0.279 | 17% | 0.675 | 0.62-1.09 | N |
|  | Aune D, 2018 [104] | high versus low intake | Feb 2018 | | 5.8-30.0 | NOS | | 10 | 311965 | 7003 | RR:0.91 (0.78-1.07) | 0.92 (0.83-1.01) | 0.244 | 0.079 | 0.086 | 44% | 0.851 | 0.63-1.32 | N |
|  | Aune D, 2018 [104] | per 5 µg/day | Feb 2018 | | 5.8-30.0 | NOS | | 8 | 292966 | 6688 | RR:0.97 (0.93-1.01) | 0.999 (0.99-1.00) | 0.184 | 0.746 | 0.049 | 51% | 0.191 | 0.88-1.07 | Y |
| vitamin K | | | | | | | | | | | | | | | | | | | |
|  | Chen H, 2019 [107] | high versus low intake | Feb 20 20 | | 1.9-16.8 | NOS | | 3 | 148437 | 2206 | HR:1.04(0.92-1.17) | 1.04 (0.92-1.17) | 0.525 | 0.525 | 0.907 | 0% | 0.884 | 0.49-2.19 | Y |
| folate acid | | | | | | | | | | | | | | | | | | | |
|  | Chen L, 2020 [102] | high versus low intake | Feb 2018 | | 4.2-19.0 | NOS | | 16 | 255458 | 8477 | RR:0.86 (0.78-0.94) | 0.85 (0.78-0.92) | 0.001 | <0.001 | 0.323 | 11% | 0.761 | 0.73-1.02 | N |
|  | Chen L, 2020 [102] | per 100 μg/day | Feb 2018 | | 4.2-19.0 | NOS | | 13 | 253511 | 8011 | RR:0.94 (0.90-0.98) | 0.95 (0.93-0.98) | 0.003 | <0.001 | 0.031 | 47% | 0.365 | 0.84-1.05 | Y |
| β-carotene | | | | | | | | | | | | | | | | | | | |
|  | Aune D, 2018 [104] | high versus low intake | Oct 2016 | | 5.8-30.0 | NOS | | 7 | 201587 | 5468 | RR:0.84 (0.75-0.94) | 0.86 (0.79-0.94) | 0.003 | 0.001 | 0.288 | 19% | 0.226 | 0.67-1.05 | Y |
| lycopene | | | | | | | | | | | | | | | | | | | |
|  | Song B, 2017 [108] | NA | Feb 2018 | | 4.8-15.9 | NOS | | 4 | 93067 | 1803 | RR:0.87 (0.73-1.04) | 0.86 (0.73-1.00) | 0.116 | 0.050 | 0.313 | 16% | 0.184 | 053-1.44 | N |
|  | Aune D, 2018 [104] | high versus low intake | Jan 2019 | | 5.8-30.0 | NOS | | 3 | 108776 | 1371 | RR:0.80 (0.63-1.01) | 0.76 (0.65-0.90) | 0.056 | 0.001 | 0.214 | 35% | 0.360 | 0.09-7.33 | N |
|  | Aune D, 2018 [104] | per 12000 µg/day | Jan 2019 | | 5.8-30.0 | NOS | | 3 | 108776 | 1371 | RR:0.76 (0.42-1.37) | 0.96 (0.80-1.15) | 0.358 | 0.650 | 0.004 | 82% | 0.313 | 0.00-582.74 | Y |
| dietary choline | | | | | | | | | | | | | | | | | | | |
|  | Mazidi M, 2019 [109] | high versus low intake | Jan 2018 | | 16.0-32.0 | NOS | | 3 | 49404 | NA | HR:1.18(0.97-1.43) | 1.18 (0.97-1.43) | 0.092 | 0.092 | 0.44 | 0% | 0.046 | 0.34-4.15 | N |
|  | Meyer K, 2017 [110] | per 100 mg/day | Mar 2019 | | 11.0 | NOS | | 2 | 20089 | 299 | RR:0.94 (0.80-1.09) | 0.94 (0.80-1.09) | 0.380 | 0.380 | 0.749 | 0% | NA | NA | Y |
| magnesium | | | | | | | | | | | | | | | | | | | |
|  | Zhao B, 2020 [111] | high versus low intake | Aug 2011 | | 5.0-30.0 | NOS | | 18 | 692998 | 20138 | RR:0.89 (0.83-0.94) | 0.89 (0.83-0.94) | <0.001 | <0.001 | 0.529 | 0% | 0.032 | 0.83-0.95 | N |
|  | Fang X, 2016 [112] | high versus low intake | May 2016 | | 4.0-30.0 | NOS | | 18 | 692887 | 14755 | RR:0.88 (0.82-0.95) | 0.87 (0.82-0.93) | 0.001 | <0.001 | 0.19 | 22% | 0.493 | 0.74-1.04 | N |
|  | Nie Z, 2013 [113] | high versus low intake | Aug 2011 | | 8.0-15.0 | Downs and Black | | 9 | 304551 | 8367 | RR:0.89 (0.82-0.97) | 0.89 (0.82-0.97) | 0.006 | 0.006 | 0.631 | 0% | 0.644 | 0.80-0.99 | N |
|  | Fang X, 2016 [112] | per 100 mg/day | Sep 2011 | | 4.0-30.0 | NOS | | 17 | 690826 | 14632 | RR:0.93 (0.89-0.97) | 0.93 (0.90-0.96) | 0.001 | <0.001 | 0.178 | 24% | 0.345 | 0.85-1.02 | Y |
|  | Nie Z, 2013 [113] | per 100 mg/day | Feb 20 20 | | 8.0-15.0 | Downs and Black | | 9 | 304551 | 8367 | RR:0.98 (0.95-1.00) | 0.97 (0.96-0.99) | 0.042 | 0.004 | 0.121 | 37% | 0.988 | 0.92-1.04 | N |
|  | Larsson S, 2012 [114] | per 100 mg/day | Feb 20 20 | | 8.0-15.0 | NA | | 7 | 241378 | 6477 | RR:0.92 (0.88-0.97) | 0.92 (0.88-0.97) | 0.001 | 0.001 | 0.705 | 0% | 0.539 | 0.86-0.98 | N |
| calcium | | | | | | | | | | | | | | | | | | | |
|  | Yang C, 2020 [115] | high versus low intake | Mar 2019 | | 1.0-7.0 | NOS | | 20 | 984562 | 9498 | RR:0.98 (0.90-1.06) | 1.01 (0.96-1.05) | 0.601 | 0.809 | 0.010 | 48% | 0.434 | 0.77-1.24 | Y |
|  | Tian D, 2015 [116] | high versus low intake | Jul 2014 | | 8.0-22.0 | NA | | 9 | 297250 | 8552 | RR:0.89 (0.76-1.04) | 0.96 (0.89-1.04) | 0.133 | 0.329 | 0.003 | 66% | 0.062 | 0.55-1.44 | N |
|  | Larsson S, 2013 [117] | <700 mg/day | Dec 2012 | | 8.0-22.0 | NOS | | 6 | 185599 | 2634 | RR:0.82 (0.76-0.89) | 0.82 (0.76-0.89) | <0.001 | <0.001 | 0.522 | 0% | 0.050 | 0.73-0.92 | N |
|  | Larsson S, 2013 [117] | ≥700 mg/day | Dec 2012 | | 8.0-22.0 | NOS | | 7 | 250551 | 6461 | RR:1.04 (1.01-1.07) | 1.04 (1.01-1.07) | 0.012 | 0.012 | 0.524 | 0% | 0.353 | 1.00-1.08 | N |
|  | Feng X, 2016 [118] | high versus low intake | Jan 2013 | | 8.0-11.0 | MOOSE | | 12 | 492755 | 9329 | RR:0.89 (0.77-1.02) | 0.98 (0.91-1.05) | 0.101 | 0.535 | 0.003 | 61% | 0.034 | 0.58-1.37 | N |
| potassium | | | | | | | | | | | | | | | | | | | |
|  | Vinceti M, 2016 [119] | high versus low intake | Aug 2016 | | 3.7-25.8 | NOS | | 19 | 639440 | 19522 | RR:0.87 (0.80-0.94) | 0.88 (0.83-0.93) | 0.001 | <0.001 | 0.017 | 46% | 0.663 | 0.68-1.12 | N |
|  | D'Elia L, 2014 [120] | high versus low intake | NA | | 5.0-19.0 | NA | | 14 | 333250 | 10659 | RR:0.81 (0.73-0.90) | 0.85 (0.79-0.91) | <0.001 | <0.001 | 0.023 | 48% | 0.062 | 0.60-1.10 | N |
|  | Aburto N, 2013 [121] | high versus low intake | Sep 2011 | | NA | GRADE | | 9 | NA | NA | RR:0.76 (0.66-0.89) | 0.80 (0.74-0.87) | <0.001 | <0.001 | 0.013 | 59% | 0.305 | 0.49-1.17 | N |
|  | D'Elia L, 2011 [122] | high versus low intake | Dec 2009 | | 5.0-19.0 | NA | | 11 | 233606 | 7066 | RR:0.79 (0.69-0.91) | 0.84 (0.77-0.91) | 0.001 | <0.001 | 0.015 | 54% | 0.108 | 0.53-1.18 | N |
|  | Larsson S, 2011 [123] | per 1000 mg/day | Mar 2011 | | 4.0-19.0 | NA | | 11 | 268276 | 8695 | RR:0.89 (0.83-0.97) | 0.91 (0.87-0.95) | 0.004 | <0.001 | 0.026 | 51% | 0.150 | 0.72-1.10 | Y |
| sodium | | | | | | | | | | | | | | | | | | | |
|  | Strazzullo P, 2009 [124] | high versus low intake | Dec 2008 | | 3.5-19.0 | Downs and Black | | 14 | 154282 | 5346 | RR:1.23 (1.06-1.42) | 1.15 (1.06-1.24) | 0.007 | <0.001 | 0.002 | 61% | 0.252 | 0.78-1.94 | N |
|  | Zhu Y, 2018 [125] | per 100 mmol/day | Oct 2017 | | 3.5-19.0 | NOS | | 8 | 77366 | NA | RR:1.10 (1.01-1.19) | 1.04 (1.01-1.08) | 0.029 | 0.022 | 0.035 | 54% | 0.105 | 0.88-1.34 | Y |
| flavonoid | | | | | | | | | | | | | | | | | | | |
|  | Tang Z, 2016 [126] | high versus low intake | Jan 2016 | | 6.1-28.0 | NOS | | 11 | 356627 | 5154 | RR:0.89 (0.82-0.97) | 0.89 (0.82-0.97) | 0.006 | 0.006 | 0.774 | 0% | 0.238 | 0.81-0.98 | N |
|  | Tang Z, 2016 [126] | per 100 mg/day | Jan 2016 | | 6.1-28.0 | NOS | | 3 | 104917 | 1547 | RR:0.91 (0.77-1.08) | 0.91 (0.77-1.08) | 0.285 | 0.285 | 0.974 | 0% | 0.640 | 0.30-2.73 | N |
|  | Wang Z, 2014 [127] | high versus low intake | Aug 2013 | | 6.1-28.0 | NA | | 10 | 280174 | 5228 | RR:0.86 (0.75-0.99) | 0.92 (0.84-1.00) | 0.035 | 0.051 | 0.035 | 50% | 0.005 | 0.59-1.25 | N |
|  | Wang Z, 2014 [127] | per 20 mg/day | Aug 2013 | | 6.1-28.0 | NA | | 10 | 280174 | 5228 | RR:0.86 (0.77-0.96) | 0.92 (0.88-0.97) | 0.005 | 0.001 | 0.000 | 70% | 0.056 | 0.63-1.18 | Y |
|  | Micek A, 2021 [128] | high versus low intake | Jan 2020 | | 4.9-25.0 | NOS | | 4 | 126085 | 2898 | RR:0.91 (0.78-1.06) | 0.93 (0.83-1.05) | 0.229 | 0.255 | 0.283 | 21% | 0.230 | 0.57-1.46 | N |
| anthocyanins | | | | | | | | | | | | | | | | | | | |
|  | Kimble R, 2019 [129] | high versus low intake | Jan 2018 | | 4.3-41.0 | NOS | | 8 | 306236 | NA | RR:1.03 (0.96-1.10) | 1.03 (0.96-1.10) | 0.411 | 0.411 | 0.484 | 0% | 0.964 | 0.95-1.12 | Y |
|  | Micek A, 2021 [128] | high versus low intake | Jan 2020 | | 4.9-25.0 | NOS | | 4 | 175754 | 3031 | RR:0.86 (0.69-1.05) | 0.90 (0.79-1.01) | 0.142 | 0.081 | 0.115 | 49% | 0.483 | 0.39-1.88 | N |

PI: prediction interval; NA: not available; MUFA：monounsaturated fatty acid; n-3 PUFA: long chain n-3 polyunsaturated fatty acid; Y: yes; N: no. NOS:Newcastle-Ottawa Scale; MOOSE: Meta-analysis Of Observational Studies in Epidemiology.
