# Supplementary material for: Role of diet in stroke incidence: an umbrella review of meta-analyses of prospective observational studies
Source: BMC Med. 2022 May 24;20:194. doi: 10.1186/s12916-022-02381-6 (PMC9128224; doi:10.1186/s12916-022-02381-6)
Supplement: Supplementary file 5 — Additional file 5: Table S5. Quality assessment of included meta-analyses using AMSTAR 2, without considering item 2 (sensitivity analysis). [file 12916_2022_2381_MOESM5_ESM.docx]

**Table S5. Quality assessment of included meta-analyses using AMSTAR 2, without considering item 2 (sensitivity analysis).**

| Author, year | 1 | 3 | 4 | 5 | 6 | 7 | 8 | 9 | 10 | 11 | 12 | 13 | 14 | 15 | 16 | Final rating |
| --- | --- | --- | --- | --- | --- | --- | --- | --- | --- | --- | --- | --- | --- | --- | --- | --- |
| Shao C, 2021 | N | Y | Y | Y | N | Y | Y | Y | N | Y | Y | Y | Y | Y | Y | moderate |
| Krittanawong C, 2021 | Y | Y | Y | Y | N | Y | Y | Y | N | Y | N | N | Y | N | N | critical low |
| Jakobsen M, 2021 | Y | Y | Y | Y | Y | Y | Y | Y | N | Y | Y | Y | Y | Y | Y | high |
| Micek A, 2021 | Y | Y | Y | Y | Y | Y | Y | Y | N | Y | Y | Y | Y | Y | Y | high |
| Chen C, 2021 | Y | Y | Y | N | Y | Y | Y | N | N | Y | Y | Y | Y | Y | Y | low |
| Mohammadifard N,2021 | Y | Y | Y | Y | N | Y | Y | Y | N | Y | Y | Y | Y | Y | Y | moderate |
| Wehrli F, 2021 | Y | Y | Y | N | Y | Y | Y | Y | N | Y | N | Y | N | Y | Y | moderate |
| Ojagbemi A, 2021 | Y | Y | Y | Y | Y | Y | Y | Y | N | Y | Y | Y | Y | Y | N | moderate |
| Zhao B, 2020 | Y | Y | Y | N | Y | Y | Y | Y | N | Y | N | Y | Y | Y | Y | moderate |
| Yang C, 2020 | Y | Y | Y | Y | N | Y | Y | Y | N | Y | N | Y | Y | Y | Y | moderate |
| Shi H,2020 | Y | Y | Y | Y | Y | Y | Y | Y | N | Y | Y | Y | Y | Y | Y | high |
| Chen L, 2020 | Y | Y | Y | Y | Y | Y | Y | Y | N | Y | N | Y | Y | Y | N | moderate |
| Morze J, 2020 | Y | Y | N | Y | Y | Y | Y | Y | N | Y | N | Y | Y | Y | Y | low |
| Mazidi M, 2020 | Y | Y | Y | Y | Y | N | N | Y | N | Y | N | Y | N | Y | Y | low |
| Chung M, 2020 | Y | Y | Y | N | N | Y | Y | Y | N | N | N | N | Y | N | N | critical low |
| Kang Z, 2020 | Y | Y | Y | Y | Y | Y | Y | Y | N | Y | Y | Y | Y | Y | Y | high |
| Tang H, 2020 | Y | Y | N | N | N | Y | Y | Y | N | Y | Y | Y | Y | Y | Y | low |
| Chen H, 2019 | Y | Y | Y | Y | Y | Y | Y | Y | N | Y | N | Y | N | N | Y | low |
| Ren Y, 2019 | Y | Y | N | Y | Y | Y | Y | Y | N | Y | Y | Y | Y | Y | Y | low |
| Schwingshackl L, 2019 | Y | Y | N | Y | N | Y | Y | Y | N | N | N | Y | Y | N | Y | critical low |
| Zhao W, 2019 | Y | Y | Y | N | N | Y | Y | Y | N | Y | Y | Y | Y | Y | Y | moderate |
| Becerra-Tomás N, 2019 | Y | Y | Y | N | Y | Y | Y | Y | Y | Y | Y | Y | Y | Y | Y | high |
| Becerra-Tomás N, 2019 | N | Y | N | N | N | Y | N | N | N | Y | N | N | N | N | N | critical low |
| Mazidi M, 2019 | Y | Y | Y | Y | Y | Y | Y | Y | N | Y | Y | Y | N | Y | N | moderate |
| Mazidi M, 2019 | N | Y | Y | Y | Y | Y | Y | N | N | Y | Y | Y | Y | Y | Y | low |
| Kimble R, 2019 | Y | Y | Y | Y | Y | Y | Y | Y | N | Y | Y | Y | Y | Y | N | moderate |
| Xu L, 2019 | Y | Y | N | N | N | Y | N | N | N | Y | N | N | N | Y | Y | critical low |
| Bechthold A, 2019 | Y | Y | Y | Y | Y | Y | Y | Y | N | Y | Y | Y | Y | Y | Y | high |
| Soedamah-Muthu S, 2018 | N | Y | N | N | N | N | N | N | N | N | N | N | Y | N | Y | critical low |
| Cheng P, 2018 | Y | Y | Y | N | Y | Y | Y | Y | N | Y | Y | Y | Y | Y | Y | moderate |
| Aune D, 2018 | Y | Y | N | N | Y | Y | N | Y | N | Y | Y | Y | Y | Y | Y | low |
| Gianfredi V, 2018 | Y | Y | Y | Y | Y | Y | Y | Y | N | Y | N | Y | Y | Y | Y | moderate |
| Qin Z, Z, 2018 | N | Y | N | Y | Y | Y | Y | Y | N | Y | Y | Y | Y | Y | Y | low |
| Aune D, 2018 | Y | Y | N | N | Y | Y | N | Y | N | Y | Y | Y | Y | Y | Y | low |
| Cheng P, 2018 | Y | Y | Y | Y | Y | Y | Y | Y | N | Y | Y | Y | Y | Y | Y | high |
| Zhu Y, 2018 | Y | Y | Y | Y | Y | Y | Y | Y | N | Y | Y | Y | Y | Y | Y | high |
| Mohammadi H, 2018 | Y | Y | Y | N | Y | Y | Y | Y | N | N | Y | Y | Y | Y | Y | low |
| Gholami F, 2017 | Y | Y | N | Y | Y | Y | Y | Y | N | Y | Y | Y | Y | Y | Y | low |
| Chen G, C, 2017 | Y | Y | Y | Y | N | Y | Y | Y | N | Y | Y | N | Y | Y | Y | low |
| Gholami F, 2017 | Y | Y | N | Y | Y | Y | Y | Y | N | Y | Y | Y | Y | Y | Y | low |
| Wu L, 2017 | Y | Y | Y | Y | Y | Y | Y | Y | N | Y | Y | Y | Y | Y | Y | high |
| Yuan S, 2017 | Y | Y | N | Y | Y | Y | Y | Y | N | Y | Y | Y | Y | Y | Y | low |
| Kim K, 2017 | Y | Y | Y | Y | Y | Y | Y | Y | N | Y | N | Y | Y | Y | Y | moderate |
| Chen G, 2017 | Y | Y | Y | Y | N | Y | Y | Y | N | Y | Y | N | Y | Y | Y | low |
| Marventano S, 2017 | Y | Y | Y | Y | N | Y | Y | Y | N | Y | Y | Y | Y | Y | Y | moderate |
| Yan Z, 2017 | Y | Y | Y | Y | Y | Y | Y | Y | N | Y | Y | Y | Y | Y | Y | high |
| Aune D, 2017 | Y | Y | N | N | N | Y | Y | N | N | Y | Y | Y | Y | Y | Y | critical low |
| Song B, 2017 | Y | Y | Y | N | Y | Y | Y | Y | N | Y | Y | Y | Y | Y | Y | moderate |
| Meyer K, 2017 | N | Y | N | N | N | N | Y | Y | N | N | N | Y | Y | Y | Y | critical low |
| Li M, 2017 | Y | Y | Y | Y | Y | Y | Y | N | N | Y | Y | Y | Y | Y | Y | low |
| Fang X, 2016 | Y | Y | Y | Y | Y | Y | Y | Y | N | Y | N | Y | Y | Y | Y | moderate |
| Vinceti M, 2016 | Y | N | N | N | Y | Y | Y | Y | N | N | N | Y | Y | Y | Y | critical low |
| Mullie P, 2016 | Y | Y | N | Y | Y | Y | Y | N | N | Y | N | Y | Y | Y | Y | critical low |
| de Goede J, 2016 | N | Y | Y | N | N | Y | Y | Y | N | N | Y | Y | Y | Y | Y | low |
| Alexander D, 2016 | Y | Y | N | Y | Y | Y | Y | N | N | Y | Y | N | Y | Y | Y | critical low |
| Pimpin L, 2016 | Y | Y | Y | Y | Y | Y | Y | Y | N | Y | N | Y | Y | Y | N | moderate |
| Narain A, 2016 | Y | Y | Y | Y | Y | Y | N | Y | N | Y | N | N | Y | N | Y | critical low |
| Yang C, 2016 | Y | Y | N | Y | Y | Y | Y | Y | N | Y | N | Y | Y | Y | N | low |
| Shao C, 2016 | Y | Y | N | N | Y | Y | Y | Y | N | Y | Y | Y | Y | Y | Y | low |
| Mayhew A, J, | Y | Y | Y | N | Y | Y | Y | Y | N | Y | Y | Y | Y | Y | N | moderate |
| Aune D, 2016 | Y | Y | N | N | N | Y | Y | Y | N | Y | Y | Y | Y | Y | Y | low |
| Aune D, 2016 | Y | N | N | N | N | Y | Y | Y | N | Y | Y | Y | Y | Y | Y | low |
| Lou D, 2016 | Y | Y | Y | Y | N | Y | Y | Y | N | Y | Y | Y | Y | Y | Y | moderate |
| Zhang X, 2016 | Y | Y | N | N | N | N | N | Y | N | N | N | Y | Y | Y | Y | critical low |
| Chen J, 2016 | Y | Y | Y | Y | Y | Y | Y | Y | N | Y | Y | Y | Y | Y | Y | high |
| Chen G, 2016 | Y | Y | Y | Y | Y | Y | Y | Y | N | Y | Y | Y | Y | Y | N | moderate |
| Li B, 2016 | N | Y | Y | Y | Y | Y | Y | Y | N | N | Y | Y | Y | Y | N | low |
| Cheng P, 2016 | Y | Y | N | N | Y | Y | Y | Y | N | Y | Y | Y | Y | Y | Y | low |
| Cheng P, 2016 | Y | Y | N | Y | N | Y | Y | Y | N | N | Y | Y | Y | Y | N | critical low |
| Tang Z, 2016 | Y | Y | Y | N | Y | Y | Y | Y | N | Y | Y | Y | Y | Y | Y | moderate |
| Feng X, 2016 | Y | Y | N | Y | Y | Y | Y | N | N | Y | Y | Y | Y | Y | Y | critical low |
| Tian D, 2015 | Y | Y | Y | N | N | N | Y | N | N | Y | Y | Y | Y | Y | Y | critical low |
| Qin L, 2016 | Y | Y | Y | Y | Y | Y | Y | N | N | Y | N | Y | Y | Y | Y | low |
| Zhang C, 2015 | Y | Y | Y | Y | Y | Y | Y | Y | N | Y | Y | Y | Y | Y | Y | high |
| Xi B, 2015 | Y | Y | Y | Y | N | Y | Y | Y | N | Y | Y | Y | Y | Y | Y | moderate |
| Zhang Z, 2015 | Y | Y | Y | Y | N | Y | Y | Y | N | Y | Y | Y | Y | Y | Y | moderate |
| Wu D, 2015 | Y | Y | Y | Y | Y | Y | Y | Y | N | Y | Y | Y | Y | Y | N | moderate |
| Cai X, 2015 | Y | Y | Y | N | Y | Y | Y | Y | N | Y | N | Y | Y | Y | Y | moderate |
| Cheng P, 2015 | Y | Y | N | N | Y | Y | Y | Y | N | N | Y | Y | Y | Y | Y | critical low |
| de Souza R, 2015 | Y | Y | Y | Y | N | Y | Y | Y | N | Y | Y | Y | Y | Y | N | moderate |
| Fang L, 2015 | Y | Y | N | Y | Y | Y | Y | N | N | Y | Y | Y | Y | Y | Y | critical low |
| D'Elia L, 2014 | Y | Y | Y | Y | N | Y | Y | Y | N | Y | Y | Y | Y | Y | N | moderate |
| Zhou D, 2014 | Y | Y | Y | Y | Y | Y | Y | N | N | Y | N | Y | Y | Y | Y | low |
| Shi Z, Q, 2014 | Y | Y | Y | Y | Y | Y | Y | Y | N | Y | Y | Y | Y | Y | Y | high |
| Luo C, 2014 | Y | Y | Y | N | Y | Y | Y | Y | N | Y | N | Y | Y | Y | Y | moderate |
| Afshin A, 2014 | Y | Y | N | Y | Y | Y | Y | Y | N | N | Y | Y | Y | Y | Y | critical low |
| Hu D, 2014 | Y | Y | N | N | N | Y | Y | Y | N | Y | Y | Y | Y | Y | N | low |
| Zhang Z, 2014 | Y | Y | N | Y | N | Y | Y | Y | N | Y | Y | Y | Y | Y | Y | low |
| Wang Z, 2014 | Y | Y | Y | Y | Y | Y | Y | N | N | Y | N | Y | Y | Y | N | low |
| Nie Z, 2013 | Y | Y | Y | Y | Y | Y | Y | Y | N | Y | N | Y | Y | N | Y | low |
| Larsson S, 2013 | Y | Y | Y | N | Y | Y | Y | Y | N | Y | N | Y | Y | Y | N | moderate |
| Aburto N, 2013 | Y | Y | Y | N | Y | Y | N | Y | N | Y | Y | Y | Y | Y | Y | moderate |
| Zhang Z, 2013 | Y | Y | Y | Y | Y | Y | Y | Y | N | Y | Y | Y | Y | Y | N | moderate |
| Threapleton D, 2013 | N | Y | Y | N | N | Y | Y | N | N | N | N | Y | Y | Y | Y | critical low |
| Chen G, 2013 | Y | Y | N | Y | Y | Y | Y | Y | N | Y | N | Y | Y | Y | Y | low |
| Chen G, 2013 | Y | Y | N | N | Y | N | Y | N | N | Y | N | Y | N | Y | Y | critical low |
| Chen G, 2013 | Y | Y | Y | N | Y | Y | Y | N | N | Y | N | Y | Y | N | Y | critical low |
| Rong Y, 2013 | Y | Y | Y | N | Y | Y | Y | Y | N | Y | Y | Y | Y | Y | Y | moderate |
| Shin J, 2013 | Y | Y | Y | N | Y | Y | Y | Y | N | Y | Y | Y | Y | Y | Y | moderate |
| Kim B, 2012 | Y | Y | Y | N | N | Y | Y | Y | N | Y | Y | Y | Y | Y | Y | moderate |
| Shen L, 2012 | Y | Y | N | N | Y | Y | Y | N | N | Y | Y | Y | Y | Y | N | critical low |
| Xun P, 2012 | Y | Y | Y | Y | Y | N | Y | N | N | Y | Y | Y | Y | Y | N | critical low |
| Kaluza J, 2012 | Y | Y | N | N | Y | Y | Y | N | N | Y | Y | Y | Y | Y | Y | critical low |
| Larsson S, 2012 | Y | Y | Y | Y | N | Y | Y | Y | N | Y | N | Y | Y | Y | N | moderate |
| Larsson S, 2012 | N | Y | Y | N | N | N | Y | N | N | N | N | Y | N | Y | N | critical low |
| Larsson S, 2012 | Y | Y | Y | N | Y | Y | Y | N | N | Y | N | Y | Y | Y | Y | low |
| Pan A, 2012 | Y | Y | Y | Y | Y | Y | Y | Y | N | Y | Y | Y | Y | Y | Y | high |
| Zhang R, 2012 | Y | Y | Y | N | Y | N | Y | Y | N | Y | N | Y | Y | Y | Y | low |
| D'Elia L, 2011 | Y | Y | Y | Y | N | Y | Y | Y | N | Y | Y | Y | Y | Y | N | moderate |
| Soedamah-Muthu S, 2011 | N | Y | N | N | Y | Y | Y | N | N | Y | N | Y | Y | Y | Y | critical low |
| Buitrago-Lopez A, 2011 | Y | Y | Y | Y | Y | Y | Y | Y | N | Y | N | Y | Y | Y | Y | moderate |
| Larsson S, 2011 | Y | Y | N | N | N | Y | N | N | N | N | N | Y | N | N | Y | critical low |
| Larsson S, 2011 | Y | Y | N | N | Y | Y | Y | N | N | Y | N | Y | Y | Y | N | critical low |
| Micha R, 2010 | Y | Y | Y | Y | N | Y | Y | Y | N | Y | N | Y | Y | N | Y | low |
| Siri-Tarino P, 2010 | Y | Y | N | Y | Y | Y | Y | Y | N | Y | N | N | Y | Y | Y | critical low |
| Elwood P, 2010 | N | Y | N | N | N | N | N | N | N | Y | N | Y | Y | N | Y | critical low |
| Arab L, 2009 | Y | Y | Y | Y | N | N | Y | N | N | Y | N | N | Y | Y | Y | critical low |
| Strazzullo P, 2009 | Y | Y | N | N | Y | N | Y | Y | N | Y | Y | Y | Y | Y | Y | critical low |
| Elwood P, 2008 | N | N | N | N | N | N | Y | N | N | N | N | N | N | N | N | critical low |
| He F, 2006 | Y | Y | Y | N | Y | Y | Y | N | N | Y | N | Y | Y | Y | Y | low |
| Dauchet L, 2005 | Y | Y | Y | N | Y | N | Y | N | N | Y | N | Y | N | Y | N | critical low |
| He K, 2004 | Y | Y | Y | Y | N | N | Y | N | N | Y | N | Y | Y | N | N | critical low |

Y: yes; N: no. AMSTAR 2: a Measurement Tool to Assess the Methodological Quality of Systematic Reviews. AMSTAR 2 evaluation items (the items in bold are considered critical):1, PICO description; **2, a priori protocol registered**; 3, research design; **4, literature search strategy;** 5, study selection in duplicate; 6, data extraction in duplicate; **7, a list of excluded individual studies;** 8, a detailed description of the included studies; **9, a satisfactory technique to assess risk of bias for the original studies;** 10, source of funding for individual studies; **11, an appropriate statistical method;** 12, effect of risk of bias on the results of single studies; **13, account for the risk of bias when interpreting the results;** 14, consideration the observed heterogeneity; **15, status of publication;** 16, conflict of interest declared. AMSTAR 2 rating:High: no or one 1 non-critical weakness. Moderate: more than 1 non-critical weakness. Low: 1 critical flaw, with or without non-critical weaknesses. Critically low: more than 1 critical flaw, with or without non-critical weaknesses.
