# Supplementary material for: Role of diet in stroke incidence: an umbrella review of meta-analyses of prospective observational studies
Source: BMC Med. 2022 May 24;20:194. doi: 10.1186/s12916-022-02381-6 (PMC9128224; doi:10.1186/s12916-022-02381-6)
Supplement: Supplementary file 6 — Additional file 6: Fig. S1 Funnel plots for the association between A) total grains, B) whole grain, C) refined grain, D) whole grain bread, E) whole grain breakfast cereals, F) rice, G) oat and incidence of stroke. Fig. S2 Funnel plots for the association between A) fruits and vegetables, B) fruits, C) vegetables, D) potato and incidence of stroke. Fig. S3 Funnel plots for the association between A) fish, B) fatty fish, C) lean fish and incidence of stroke. Fig. S4 Funnel plots for the association between A) meat, B) red meat, C) processed meat, D) processed red meat, E) fresh red meat, F) white meat (poultry) and incidence of stroke. Fig. S5 Funnel plots for the association between eggs and incidence of stroke. Fig. S6 Funnel plots for the association between A) legumes, B) soy, C) nut, D) peanuts, E) tree nuts, F) walnuts, G) peanut butter, H) nut plus peanut butter and incidence of stroke. Fig. S7 Funnel plots for the association between A) dairy products, B) milk, C) cheese, D) cream, E) butter, F) yogurt and incidence of stroke. Fig. S8 Funnel plots for the association between chocolate and incidence of stroke. Fig. S9 Funnel plots for the association between A) coffee, B) tea, C) sugar-sweetened beverages, D) artificially sweetened beverage and incidence of stroke. Fig. S10 Funnel plots for the association between A) protein, B) animal protein, C) plant protein and incidence of stroke. Fig. S11 Funnel plots for the association between A) saturated fat, B) MUFA, C) n-3 PUFA, D) cholesterol, E) α-linolenic acid and incidence of stroke. Fig. S12 Funnel plots for the association between carbohydrate and incidence of stroke. Fig. S13 Funnel plots for the association between A) total fiber, B) soluble dietary fiber, C) insoluble dietary fiber, D) cereal fiber, E) fruit fiber, F) vegetable fiber and incidence of stroke. Fig. S14 Funnel plots for the association between A) vitamin B6, B) vitamin B12, C) vitamin C, D) vitamin D, E) vitamin E, F) vitamin K, G) folate a [file 12916_2022_2381_MOESM6_ESM.pdf]

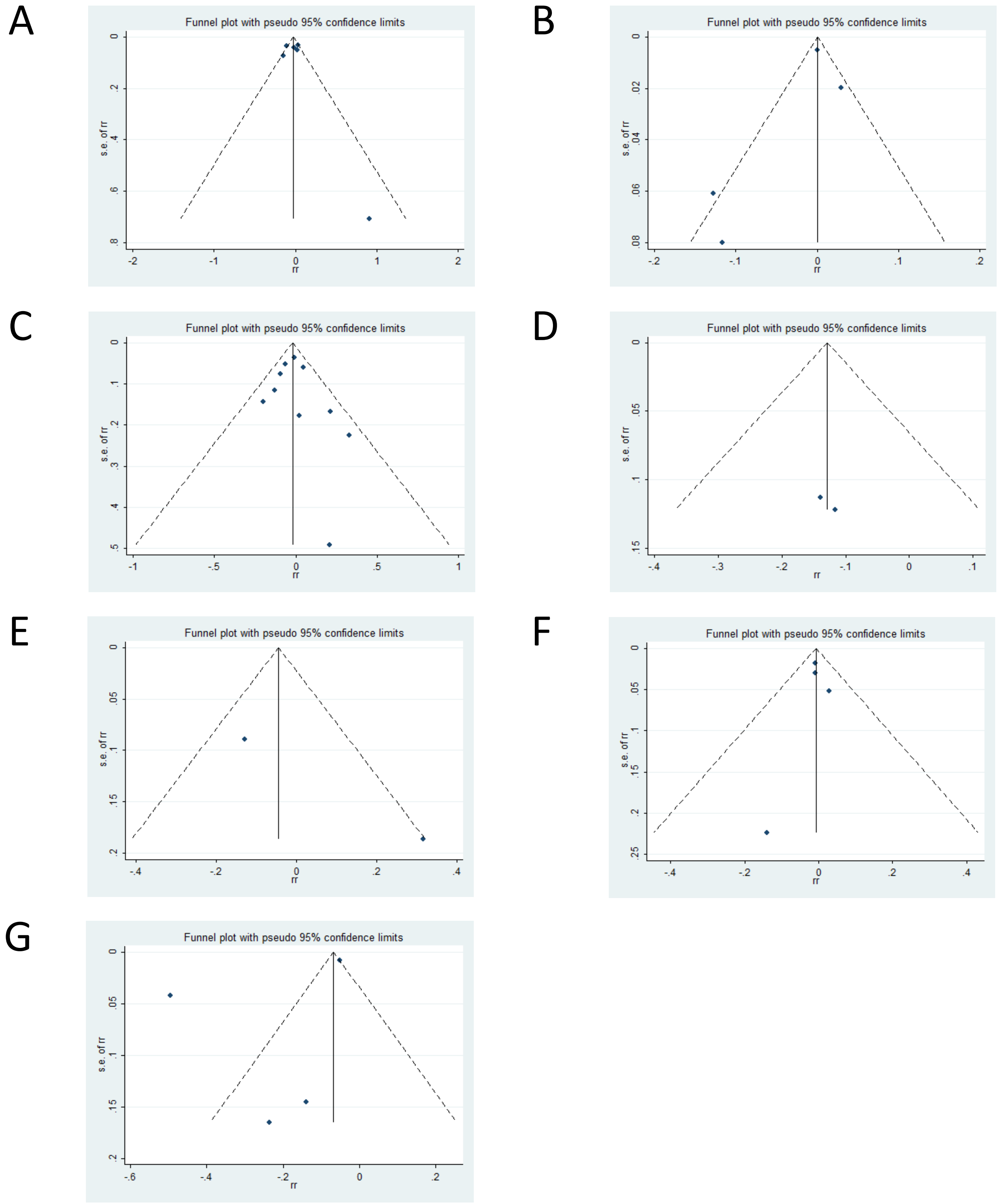

Fig. S1 Funnel plots for the association between A) total grains, B) whole grain, C) refined grain, D) whole grain bread, E) whole grain breakfast cereals, F) rice, G) oat and incidence of stroke.

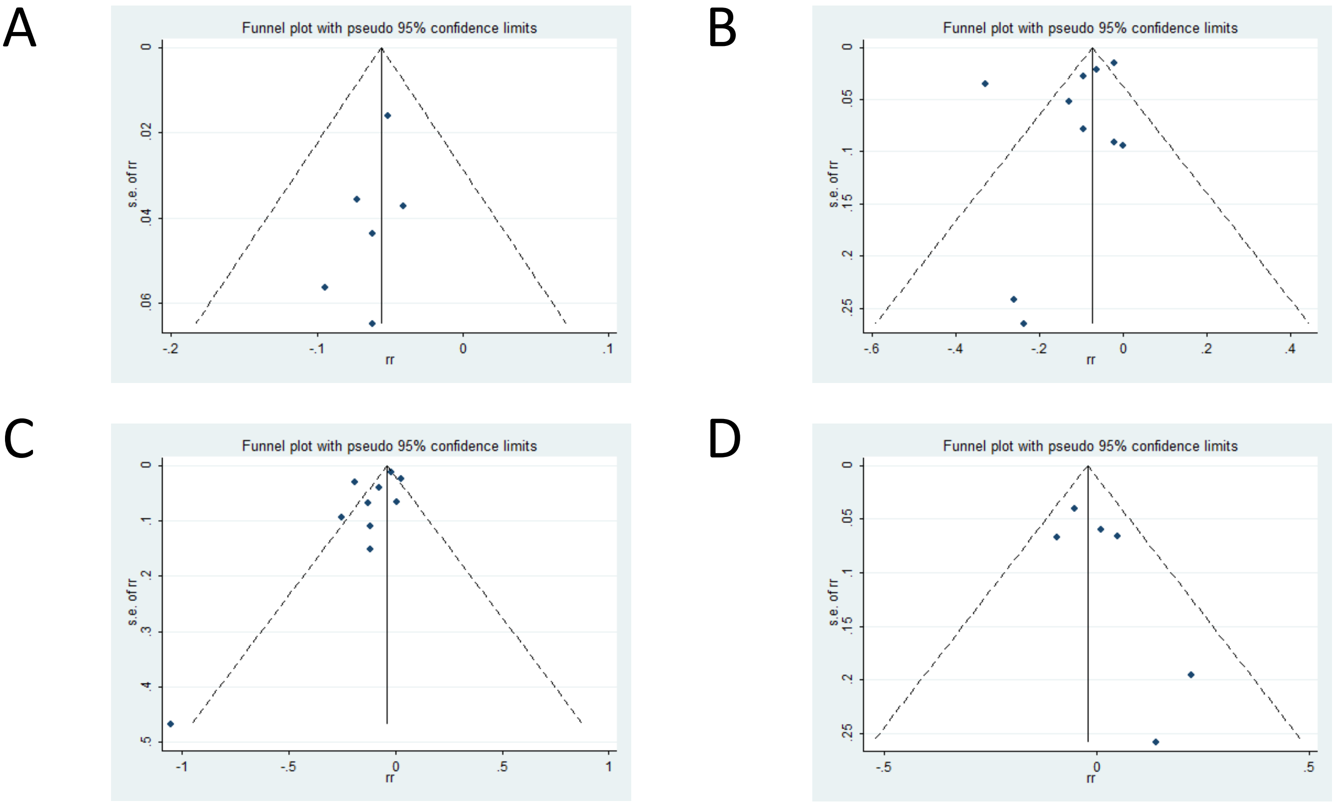

Fig. S2 Funnel plots for the association between A) fruits and vegetables, B) fruits, C) vegetables, D) potato and incidence of stroke.

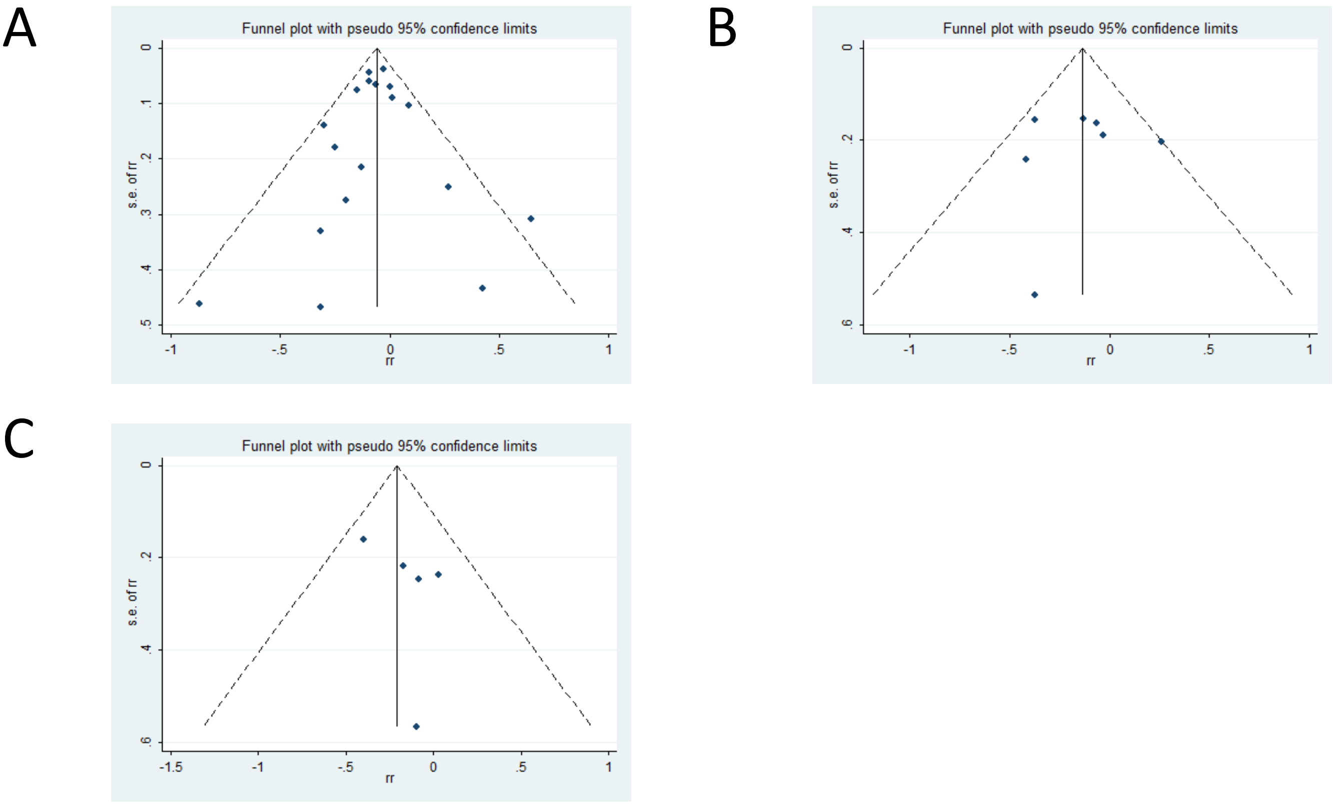

Fig. S3 Funnel plots for the association between A) fish, B) fatty fish, C) lean fish and incidence of stroke.

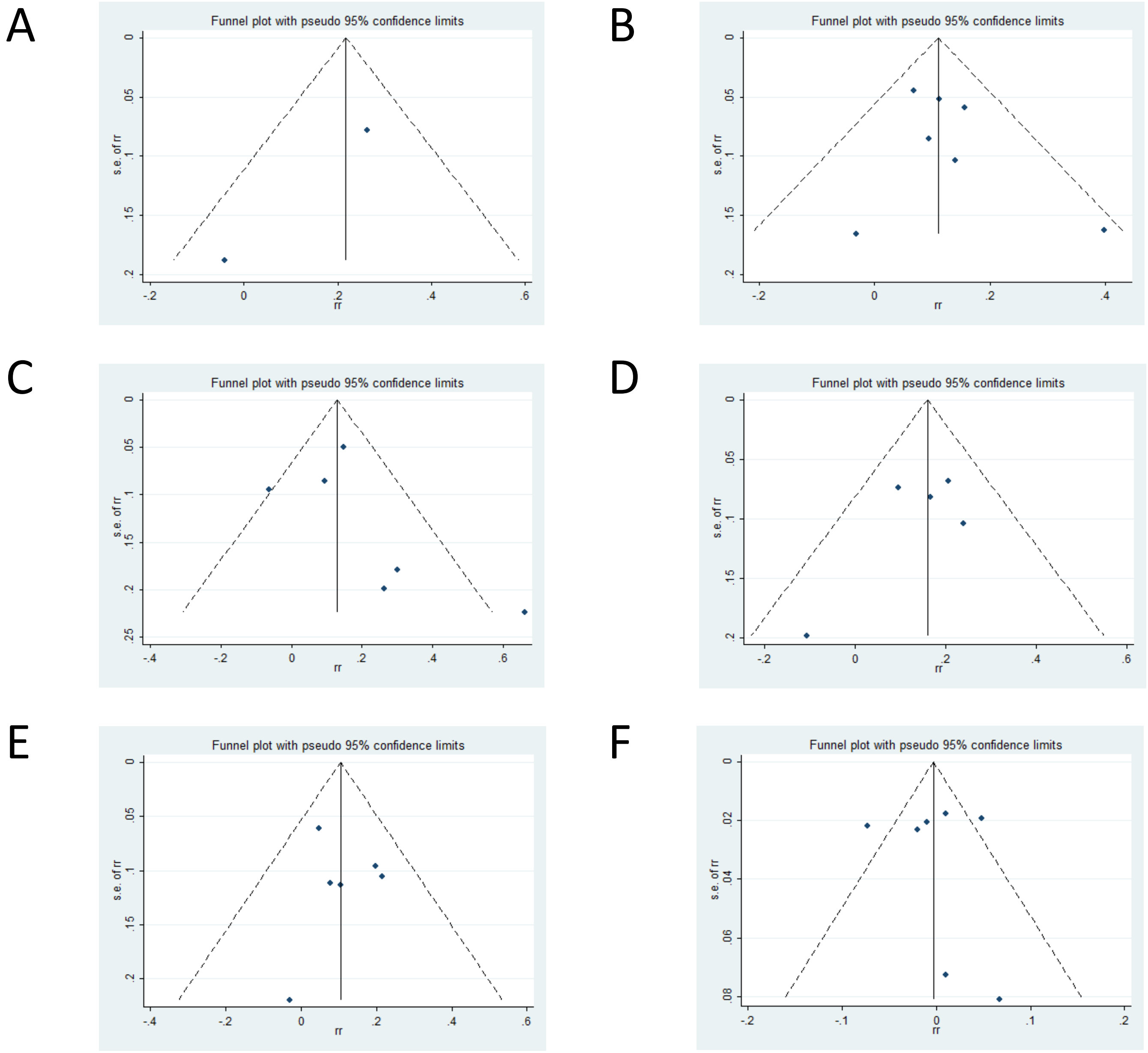

Fig. S4 Funnel plots for the association between A) meat, B) red meat, C) processed meat, D) processed red meat, E) fresh red meat, F) white meat (poultry) and incidence of stroke.

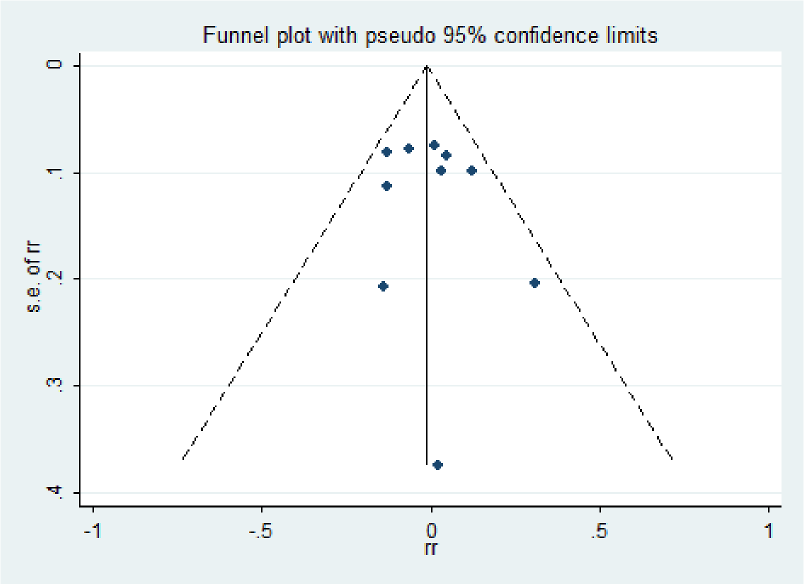

Fig. S5 Funnel plots for the association between eggs and incidence of stroke.

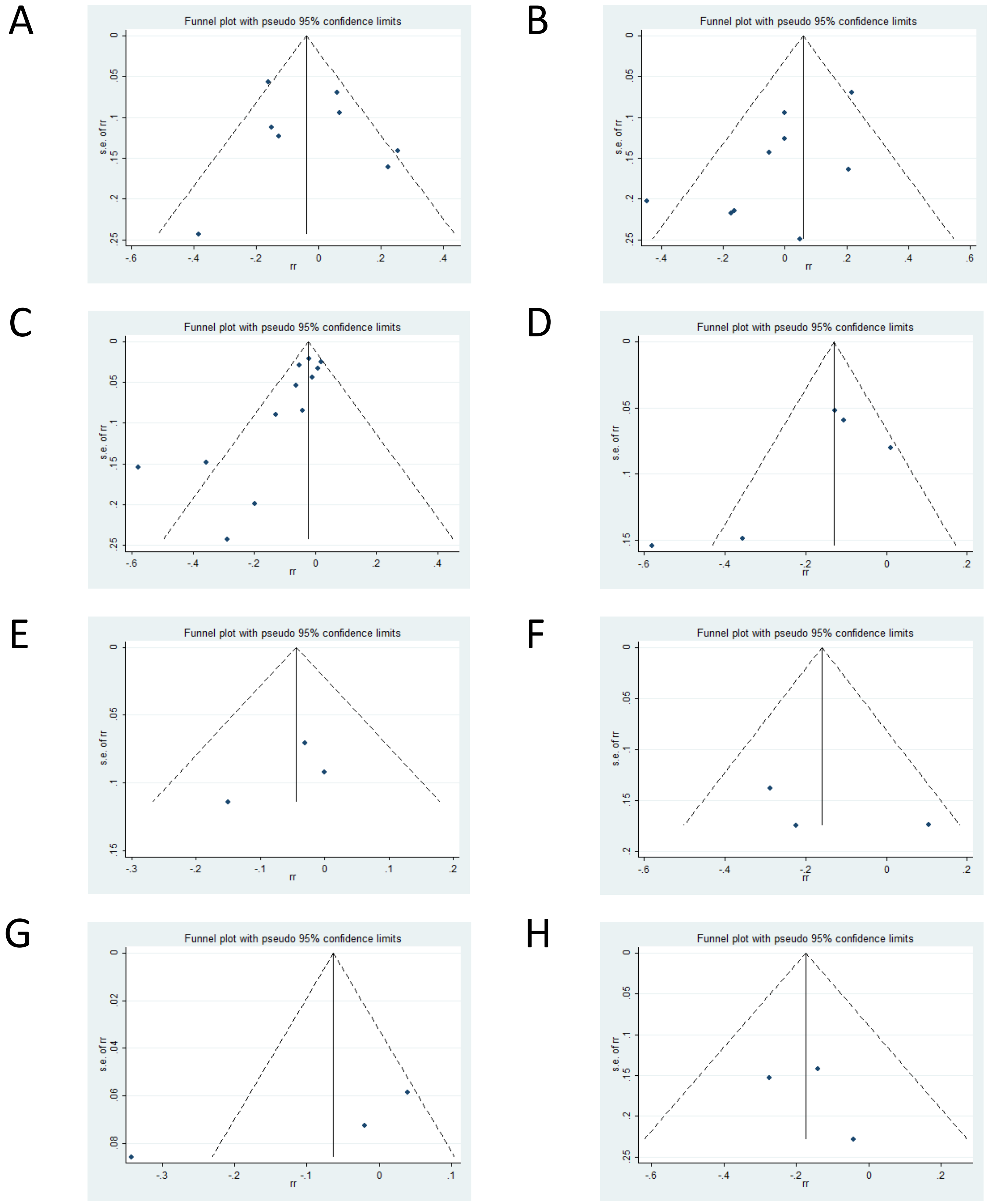

Fig. S6 Funnel plots for the association between A) legumes, B) soy, C) nut, D) peanuts, E) tree nuts, F) walnuts, G) peanut butter, H) nut plus peanut butter and incidence of stroke.

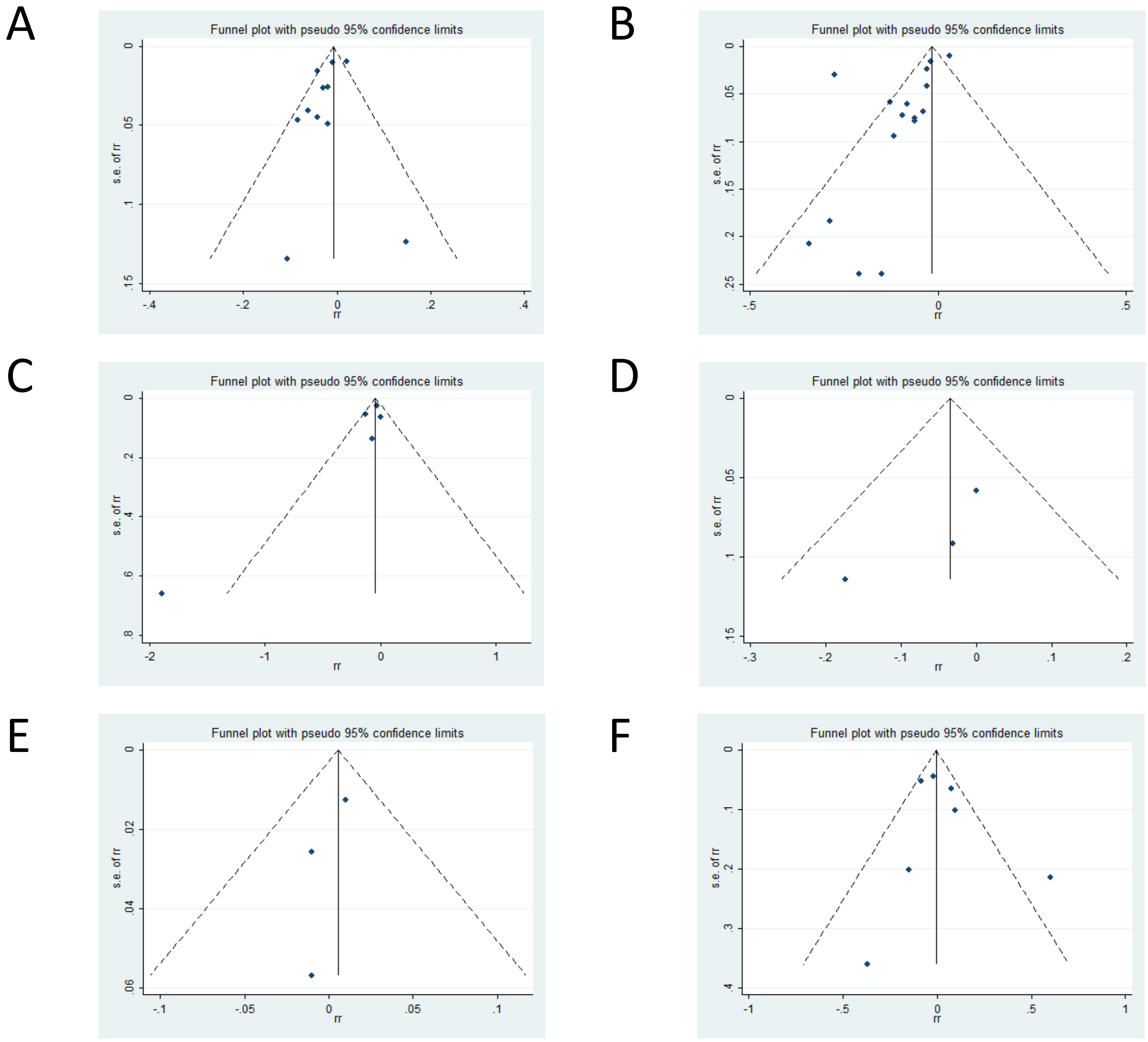

Fig. S7 Funnel plots for the association between A) dairy products, B) milk, C) cheese, D) cream, E) butter, F) yogurt and incidence of stroke.

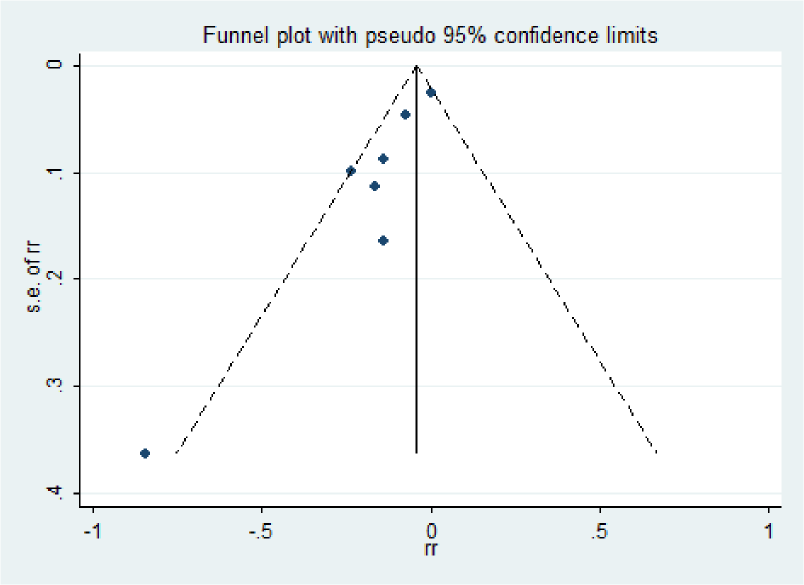

Fig. S8 Funnel plots for the association between chocolate and incidence of stroke.

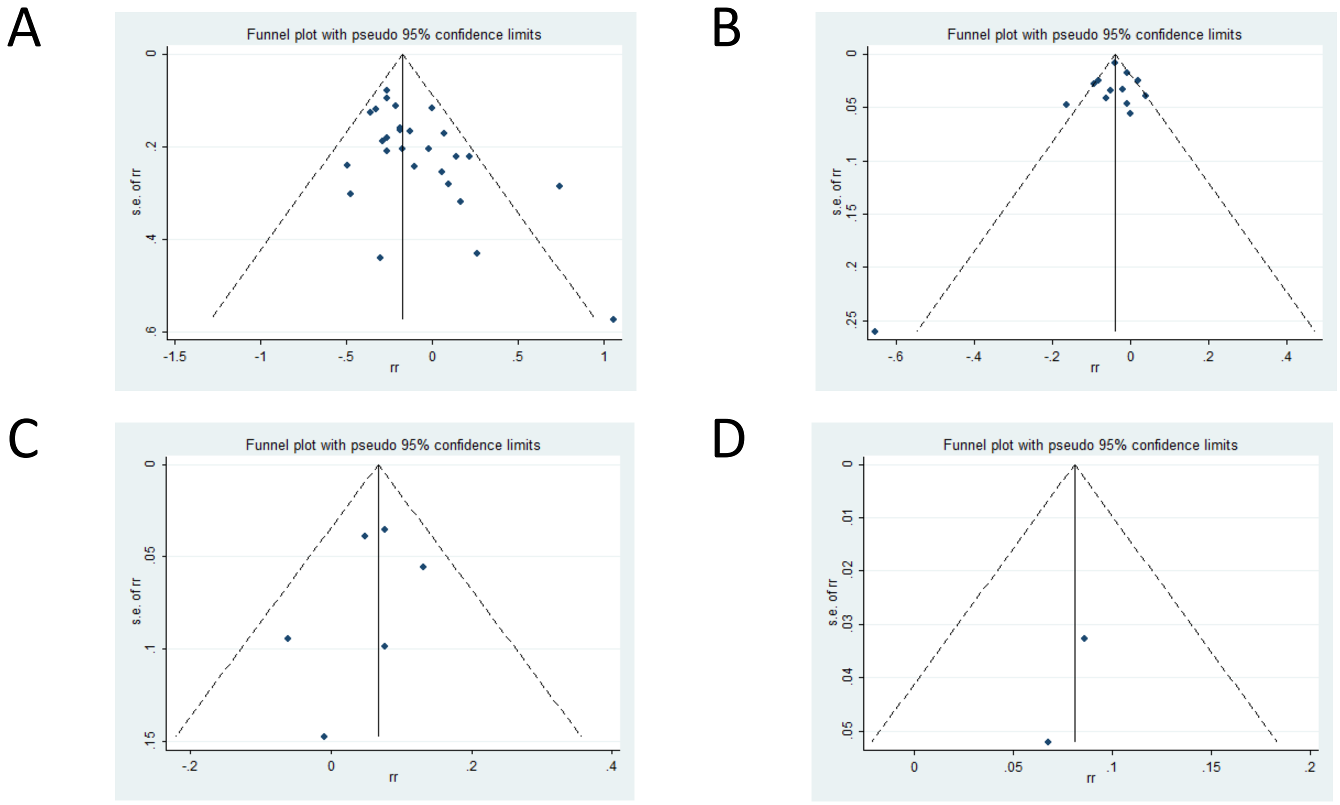

Fig. S9 Funnel plots for the association between A) coffee, B) tea, C) sugar-sweetened beverages, D) artificially sweetened beverage and incidence of stroke.

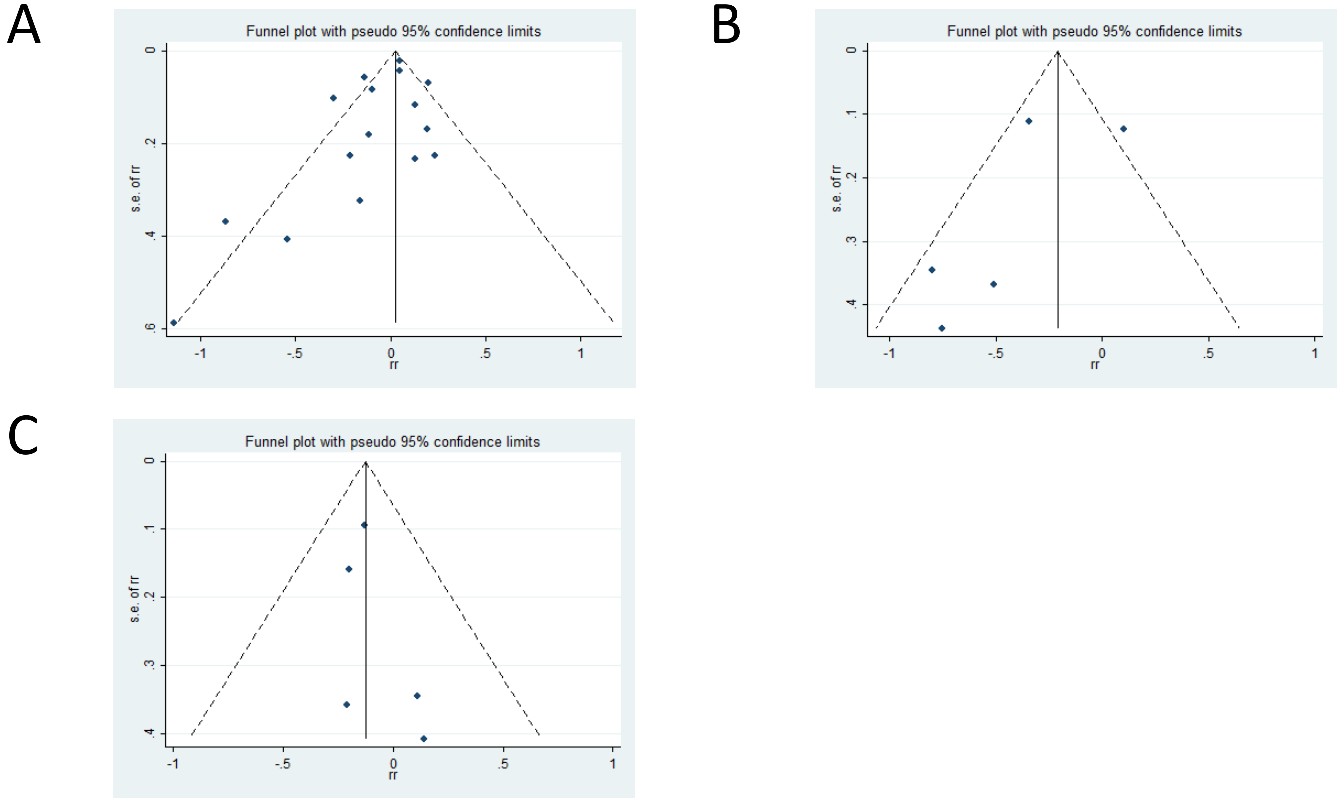

Fig. S10 Funnel plots for the association between A) protein, B) animal protein, C) plant protein and incidence of stroke.

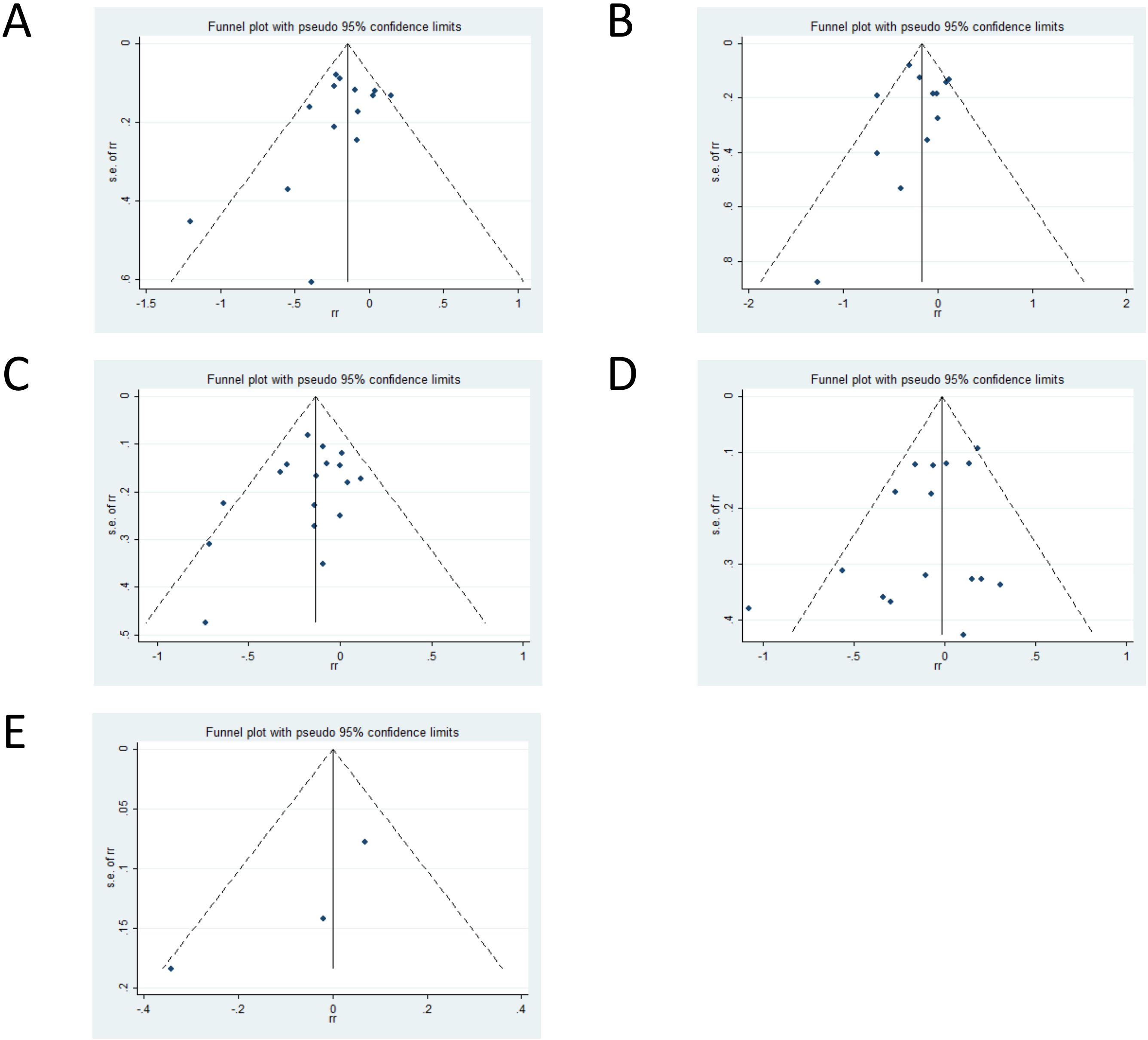

Fig. S11 Funnel plots for the association between A) saturated fat, B) MUFA, C) n-3 PUFA, D) cholesterol, E) α-linolenic acid and incidence of stroke.

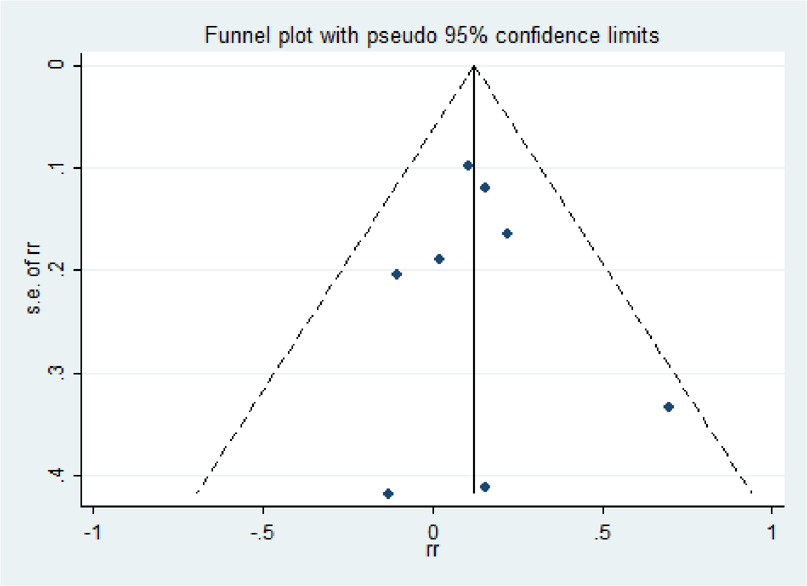

Fig. S12 Funnel plots for the association between carbohydrate and incidence of stroke.

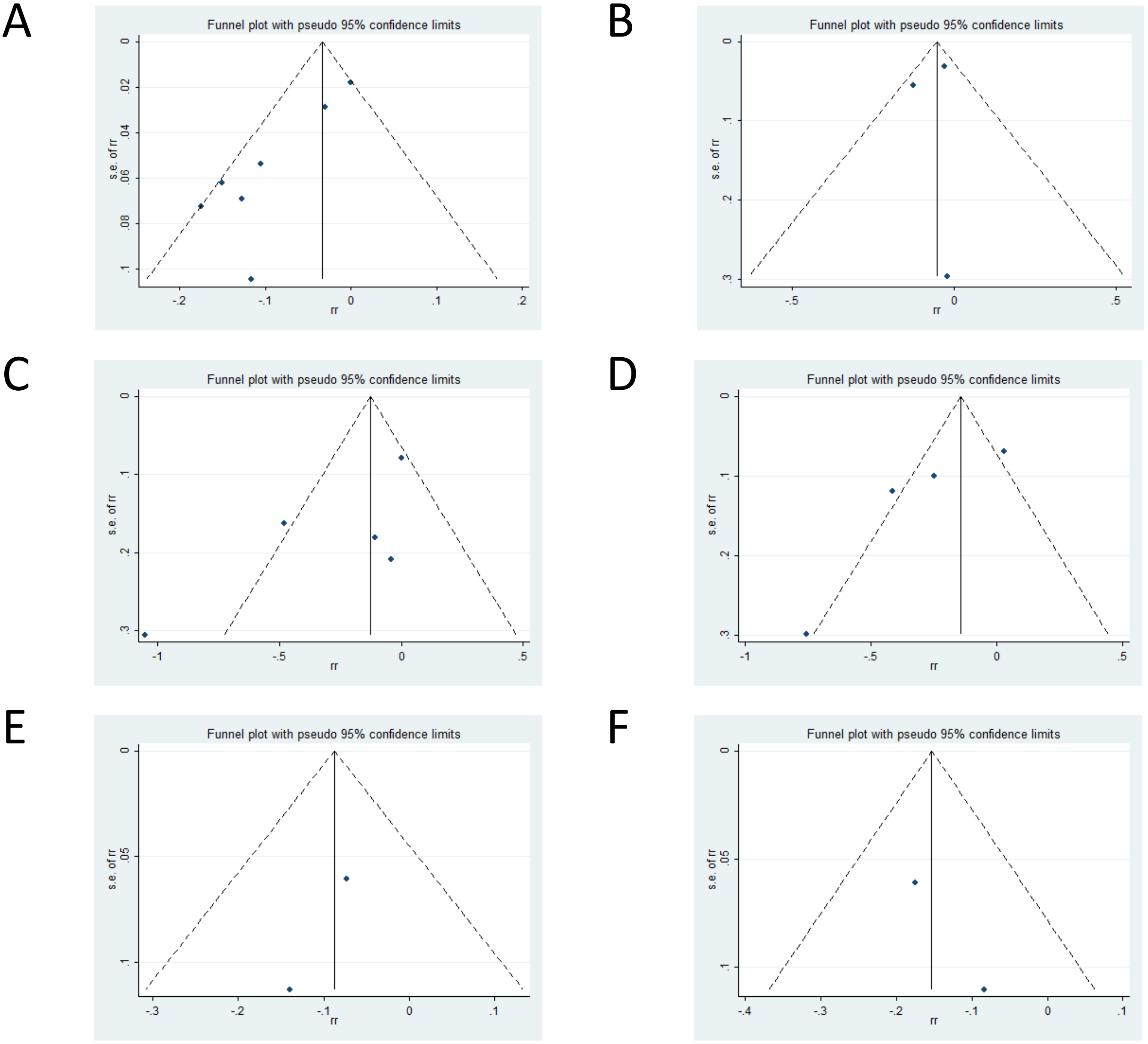

Fig. S13 Funnel plots for the association between A) total fiber, B) soluble dietary fiber, C) insoluble dietary fiber, D) cereal fiber, E) fruit fiber, F) vegetable fiber and incidence of stroke.

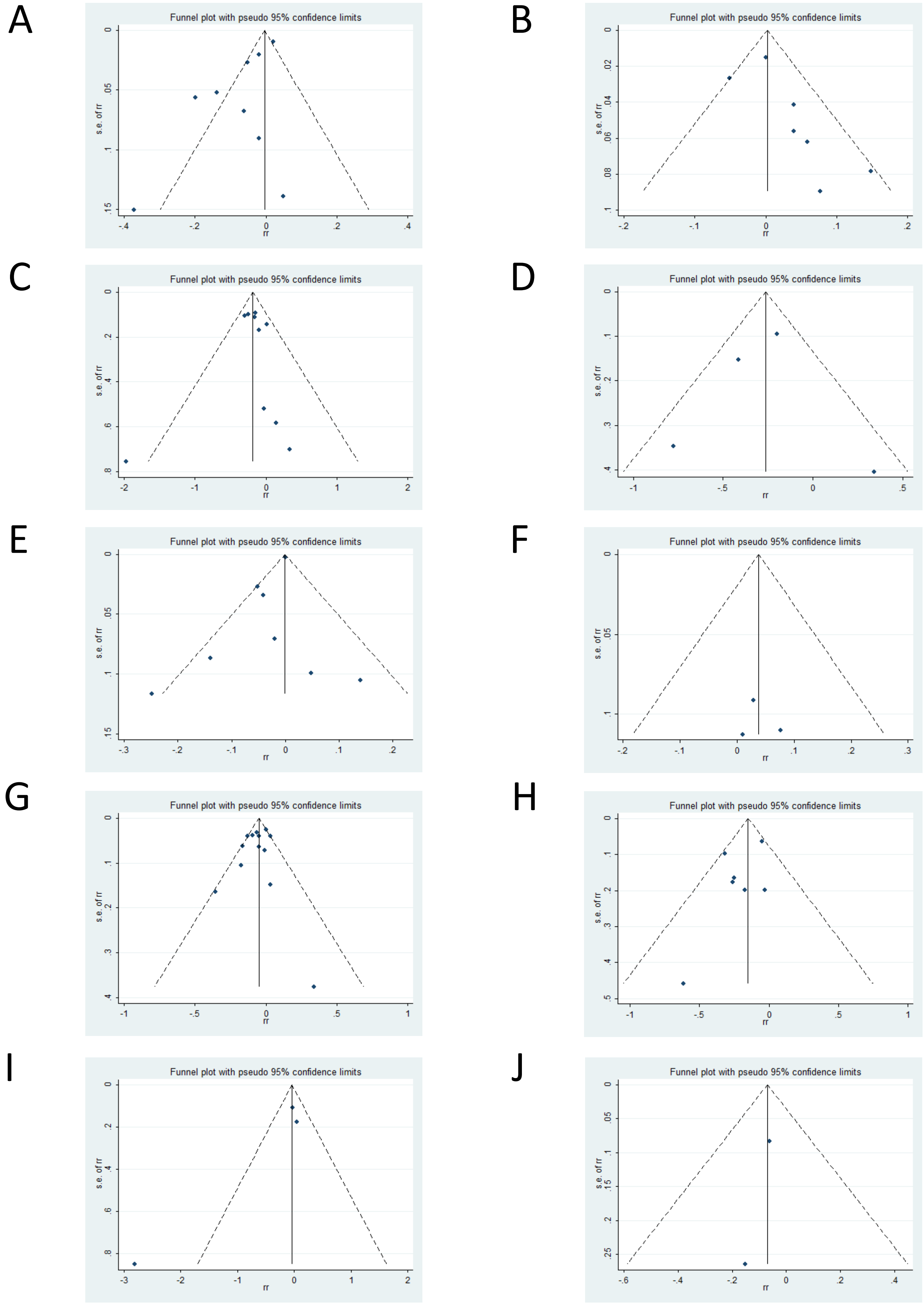

Fig. S14 Funnel plots for the association between A) vitamin B6, B) vitamin B12, C) vitamin C, D) vitamin D, E) vitamin E, F) vitamin K, G) folate acid, H) β-carotene, I) lycopene, J) dietary choline and incidence of stroke.

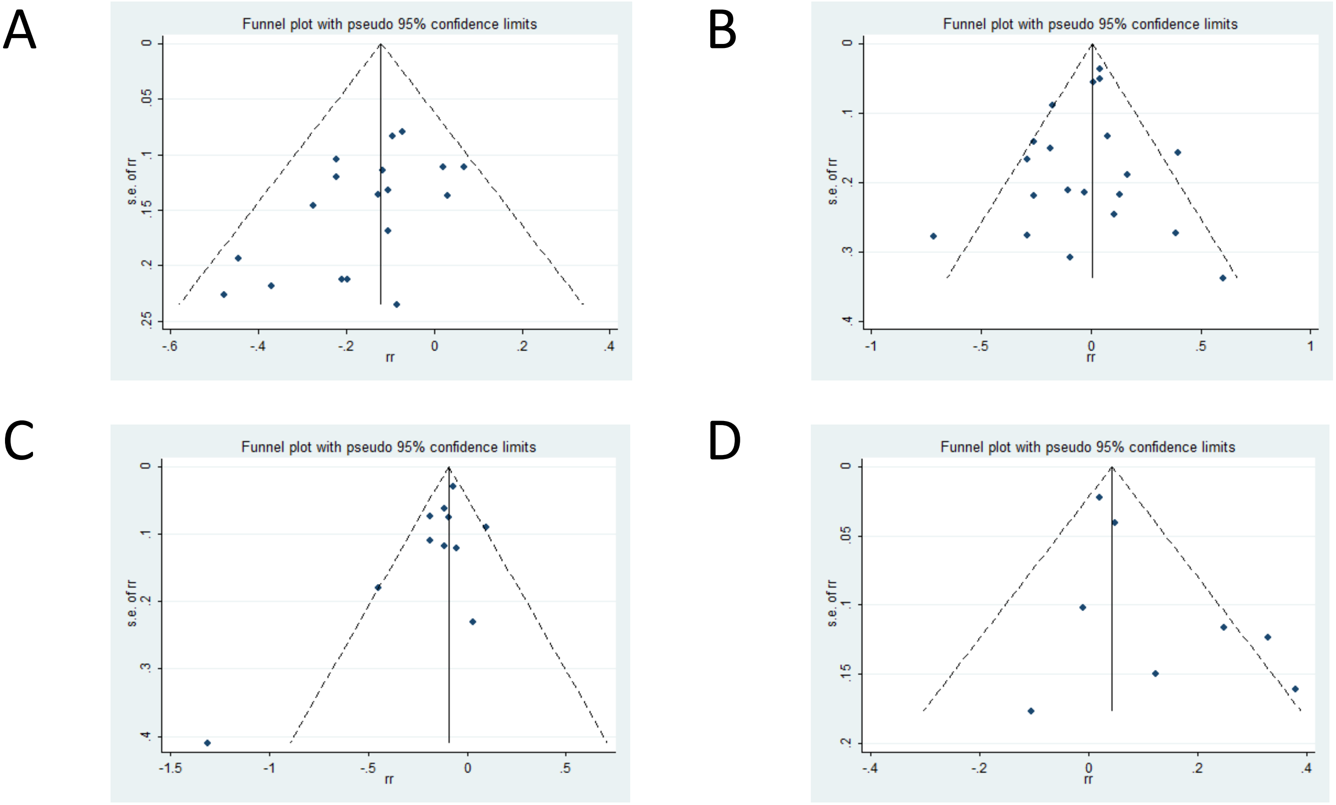

Fig. S15 Funnel plots for the association between A) magnesium, B) calcium, C) potassium, D) sodium and incidence of stroke.

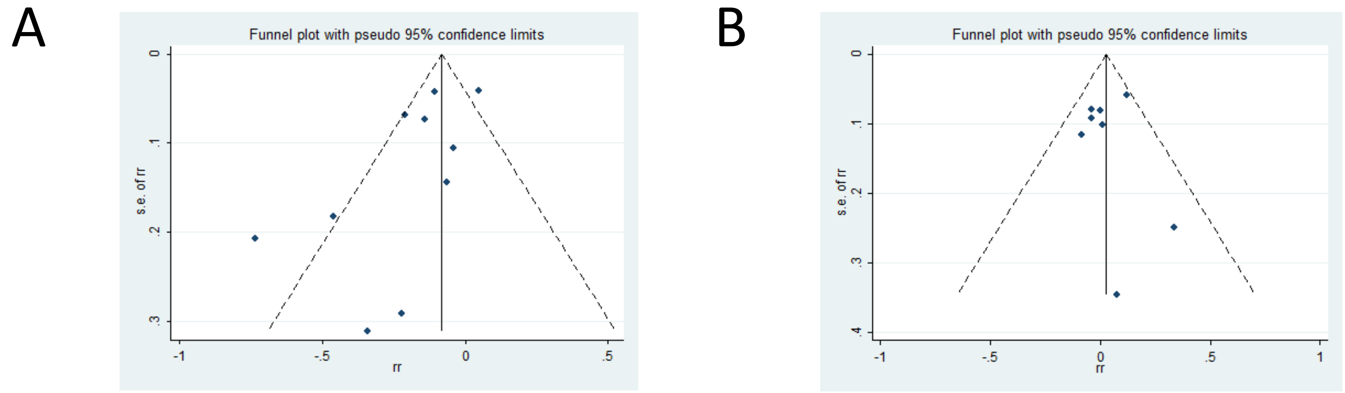

Fig. S16 Funnel plots for the association between A) flavonoid, B) anthocyanins and incidence of stroke.
